# Sub-millimeter quantification of alveolar bone loss using automated 40 MHz high-frequency ultrasound: A proof-of-concept *ex vivo* validation study

**DOI:** 10.1371/journal.pone.0349815

**Published:** 2026-06-08

**Authors:** Tamer Abdelrehim, Baiyan Qi, Bryant Tran, Mirali Pandya, Jesse V. Jokerst, Casey Chen

**Affiliations:** 1 Aiiso Yufeng Li Family Department of Chemical and Nanoengineering, University of California San Diego, La Jolla, California, United States of America; 2 Herman Ostrow School of Dentistry, University of Southern California, Los Angeles, California, United States of America; 3 Materials Science and Engineering Program, University of California San Diego, La Jolla, California, United States of America; Danube Private University, AUSTRIA

## Abstract

This proof-of-concept study evaluated the performance of high-frequency ultrasound (HFUS, 40 MHz) for monitoring changes in alveolar bone loss and determining its detection capability and measurement precision. The study aimed to quantify system error, minimum detectable change, and agreement with a photographic ground truth measurement. An *ex vivo* model was used in which controlled bone loss increments were created and measured using both manual and automated HFUS methods. Measurements were compared to calibrated photographic measurements as the reference standard. System error was calculated using repeated baseline measurements, and detection capability was assessed using sensitivity analysis across incremental bone loss values. Agreement between methods was evaluated using Bland–Altman analysis and intraclass correlation coefficients (ICC). The system error ranged from 77 µm for automated image-registration measurements to 113 µm for manual measurements. The minimum detectable bone loss increment was experimentally determined to be approximately 138 µm. Detection sensitivity ranged from 79% to 100%, depending on the measurement method and increment size. Automated ultrasound measurements showed strong agreement for longitudinal change tracking, with a best-case bias of −18 µm, corresponds to less than 10% error relative to a typical 200 µm bone change increment and excellent reliability (ICC = 0.89–0.99). No significant systematic bias was observed compared with photographic measurements (*P* > 0.05), indicating good agreement between methods. High-frequency ultrasound demonstrated the ability to detect small longitudinal changes in alveolar bone loss and to track bone loss progression over time with high measurement precision. The main contribution of this study is demonstrating that HFUS can be used as a radiation-free method for monitoring longitudinal changes in alveolar bone loss rather than relying solely on absolute measurement accuracy.

## Introduction

Periodontitis is one of the two leading diseases in dentistry, along with dental caries [1], and is associated with tooth loss, reduced quality of life, and systemic conditions such as diabetes [2]. In patients suffering from periodontitis, bacteria-induced inflammatory responses destroy soft and hard tissues supporting the teeth (i.e., the periodontium) [3]. Conventional periodontal assessment combines clinical probing to evaluate soft tissue attachment loss with radiography to assess bone levels. While simple and universally accepted, probing cannot directly evaluate the underlying bone and is imprecise. Radiography, particularly plain films, remains the standard for bone assessment but possesses fundamental limitations that hinder early and accurate diagnosis. These include its two-dimensional projection of a three-dimensional anatomy, which obscures critical details through anatomical superimposition [4], and a well-documented insensitivity that requires substantial bone mineral loss before defects become visible [5]. While three-dimensional imaging such as CBCT can overcome some geometric limitations, its use of ionizing radiation and higher cost render it unsuitable for routine monitoring [6]. Furthermore, the subjective interpretation of radiographs can lead to underestimation or overestimation of bone levels, resulting in delayed diagnoses or inappropriate treatment decisions [7].

Significant progress has been made in automated three-dimensional diagnostic workflows, particularly with artificial intelligence systems applied to cone-beam CT (CBCT) and dental radiographic imaging. Recent studies have demonstrated AI-based detection, segmentation, and analysis of dental structures and alveolar bone from CBCT and radiographic images with high accuracy and reliability [8–12]. These systems can delineate complex anatomical structures and automate diagnostic workflows, establishing a new standard for volumetric assessment and digital dentistry. However, these AI-based systems are primarily designed for anatomical segmentation, structural detection, and treatment planning rather than for detecting small longitudinal bone changes with defined measurement precision and detection thresholds. As a result, a fundamental diagnostic gap remains in the quantitative monitoring of small bone-level changes over time. This gap between the dynamic progression of periodontitis and the static, insensitive nature of radiographic assessment highlights the need for a high-precision, radiation-free method capable of detecting small longitudinal bone changes, which is the focus of the present study.

A tool capable of precisely measuring subtle bone-level changes with a defined minimum detectable threshold and low system error is still lacking, which may delay detection of early bone loss and progression of periodontal disease. High-frequency ultrasound (HFUS) is a promising, radiation-free imaging modality capable of imaging both hard and soft tissues and identifying key anatomical landmarks relevant to periodontal assessment [13,14]. The use of a 40 MHz transducer provides high axial resolution (~40 µm) [15], suggesting the potential to detect small bone-level changes beyond the detection capability of conventional radiography. Previous ultrasound studies for periodontal assessment have demonstrated the feasibility of ultrasonography for imaging periodontal and peri-implant structures [16 23]. Early studies using lower-frequency ultrasound systems (e.g., around 20 MHz) were able to visualize periodontal tissues but were limited by insufficient spatial resolution for detecting small bone-level changes [17]. More recent intraoral ultrasound studies have evaluated periodontal structures, crestal bone dimensions, and peri-implant tissues and have demonstrated good repeatability and clinical feasibility [18–23]. Photoacoustic imaging has also been explored for periodontal assessment [24]. However, many of these studies focused primarily on qualitative visualization, anatomical imaging, or single time-point measurements rather than quantitative validation of measurement precision, system error, and minimum detectable bone loss. In addition, earlier ultrasound systems often operated at lower frequencies with lower spatial resolution compared to modern high-frequency ultrasound systems [15,16,25]. Therefore, the present study differs from previous work by using a 40 MHz high-frequency ultrasound system and by focusing on quantitative validation of measurement performance, including system error, agreement analysis, and detection capability for small incremental bone loss.

Periodontitis is a dynamic disease characterized by progressive tissue destruction over time, whereas most current diagnostic tools, particularly radiography, provide static assessments and are relatively insensitive to small longitudinal changes. The diagnostic window for early intervention is frequently missed because conventional radiographs require substantial mineral loss before defects become visible. Therefore, a measurement method capable of detecting small longitudinal changes with high precision may be more clinically valuable for monitoring disease progression than methods focused solely on absolute bone level measurements.

Therefore, the purpose of this study was to quantify the measurement performance of high-frequency ultrasound for detecting and monitoring alveolar bone loss using a controlled *ex vivo* model. Specifically, we aimed to (i) quantify the system error of ultrasound measurements and compare them to photographic reference measurements, and (ii) determine the minimum amount of bone loss that can be reliably detected.

The null hypothesis of this study was that high-frequency ultrasound measurements would not differ significantly from photographic reference measurements for quantifying alveolar bone loss and would not be capable of detecting small incremental bone loss changes.

Therefore, this study does not aim to replace radiography for absolute bone level measurement, but rather to determine whether high-frequency ultrasound can detect small longitudinal changes in alveolar bone loss with high precision and a defined detection threshold.

## Materials and methods

### *Ex vivo* model and defect creation

A mandible from a 6-month-old male Yorkshire swine was procured from a USDA-approved abattoir. Three teeth (M2, M3, and PM4) were analyzed across multiple standardized bone-loss stages (baseline + incremental bone removal), each measured by three blinded examiners using multiple independent frames per stage in addition to the automatic measurement methods. This design generated a robust dataset suitable for quantitative analysis and inter-observer validation.

The specimen was transported on ice and stored at −20 °C immediately upon receipt. Twenty-four hours before experimentation, it was thawed at 4 °C and sectioned along the sagittal plane to isolate segments containing the selected teeth. The teeth were chosen based on consistent anatomical morphology, intact buccal bone plates, and accessibility for standardized defect creation.

An intrasulcular incision was made from the distobuccal area of the third mandibular molar (M3) to the mesiobuccal area of the fourth mandibular premolar (PM4) using a #15 surgical blade (Henry Schein Inc., Melville, NY, USA). A full-thickness mucoperiosteal flap was elevated with a periosteal elevator (#9 Molt double-ended periosteal elevator; Henry Schein Inc., Melville, NY, USA) to expose the underlying alveolar bone. Small holes were drilled on the buccal surface at the central region of each tooth (M3, M2, and PM4), approximately 3–4 mm above the initial alveolar bone level and within the lateral FOV for ultrasound frame. Stainless steel ball markers (1 mm diameter) were fixed in the drilled holes using cyanoacrylate adhesive (Gorilla Super Glue; Gorilla Glue Company, Cincinnati, OH, USA) to serve as stable fiducial reference markers for calibration, motion tracking, and bone level measurements [14]. Two steel balls were positioned above and below the initial alveolar bone level to provide a two-point reference for pixel-to-micron calibration. Under ultrasound scan, these reference steel balls are a clear ultrasound ring marker that ensure comparison of the same frame between different bone loss stages at different times. The mandibles were then secured on a C-clamp holder with articulating platforms (Thorlabs, Inc., Newton, NJ, USA) [26]. The distance between steel balls on each tooth was measured using a digital caliper with 0.01-mm resolution (Neiko Tools USA, CA, USA). The markers were placed in the central buccal region to minimize mesial and distal anatomical variations and to ensure consistent vertical reference measurements. This method was used only for the *ex vivo* experimental model and is not intended for clinical application. For clinical ultrasound measurements, the cementoenamel junction (CEJ) was used as the anatomical reference point. The use of fixed reference markers in this study is conceptually similar to our previous studies [14] in which use of fixed structures, such as the steel balls, were used as reference points for measuring the position of CEJ using imaging HFUS.

The buccal bone was removed incrementally using a double-ended bone chisel (13K/13KL; Henry Schein Inc., Melville, NY, USA) and a #2 Ochsenbein single chisel (Henry Schein; Henry Schein Inc., Melville, NY, USA). Six stages of bone removal were prepared for teeth M3 and PM4, and four stages for tooth M2. The amount of bone removed at each stage was targeted to ~200 µm increments by an experienced operator. The precise, final amount of bone loss for each stage was subsequently determined by post hoc photographic ground truth measurement, and these values were used for all subsequent analyses. While the manual creation process introduced some variability in defect morphology, the subsequent post hoc quantification provided the robust ground truth. The removal was then quantified by comparing the photographic ground truth measurements between stages.

### Study design and sample considerations

This study was designed as a methodological proof-of-concept and system validation study rather than a clinical hypothesis-testing study; therefore, a formal a priori sample size calculation was not performed. A post hoc power analysis was conducted (see Supporting Information S1 Text) to assess the statistical sensitivity of the experimental design [27–29].

The experimental design focused on repeated measurements across controlled bone loss stages to quantify system error, agreement, and detection capability, which were the primary outcomes of this validation study. The use of a single mandible specimen represents convenience sampling, and no randomization was performed. However, the large number of repeated measurements across stages and independent observers allowed robust estimation of measurement variability, system error, and agreement statistics, which were the main objectives of this methodological validation study.

The post hoc power analysis, based on the observed detection threshold (138 µm), system error range (77–113 µm), and repeated-measurement design, indicated a large, standardized effect size (Cohen’s d ≈ 1.22–1.79) and an estimated statistical power exceeding 95%, supporting the adequacy of the study design for detecting clinically relevant bone loss changes.

### Specimen selection criteria

A single porcine mandible specimen with intact teeth and surrounding alveolar bone was selected for the *ex vivo* model. Teeth with visible fractures, severe anatomical defects, or existing bone loss were excluded. The specimen was selected to provide stable anatomical landmarks and intact alveolar bone for controlled defect creation and measurement validation.

### Image acquisition

#### Photographic ground truth setup.

Throughout all bone removal stages, the jaw segment was consistently fixed at a predefined marked position using the same C-clamp holder with articulating platforms to ensure identical alignment and precise repositioning. High-resolution photographs were acquired using a 12-megapixel CMOS camera (EOS Rebel T3; Canon Inc., Tokyo, Japan) under flash and standard room lighting conditions (ISO-800, f/5.6, and exposure time: 1/60 sec) equipped with a 100-mm macro lens (Canon Inc., Tokyo, Japan). The camera was mounted on a stand to maintain a consistent orthogonal view and a fixed focal distance of 120 ± 1 mm, minimizing parallax error. The setup included controlled lighting, and images were captured using the camera’s built-in software connected to a PC to eliminate hand movement during capture. At each bone loss stage, the separated gingiva was held with tweezers to expose the profile of enamel and alveolar bone with fixed steel ball fiducial markers, and four frames were recorded.

The known, pre-measured distance between the fixed fiducial markers (steel balls) was used to calibrate a pixel-to-micron ratio for each image, providing a consistent scale. This calibration yielded an average photographic pixel size of 18.0 ± 0.5 µm. This photographic setup provided the reference (“gold standard”) measurement for bone level changes.

### Ultrasound imaging setup

Ultrasound imaging was performed using a high-frequency ultrasound imaging system (Vevo F2; FUJIFILM VisualSonics, Toronto, ON, Canada) [30] equipped with a linear-array transducer (UHF57x; FUJIFILM VisualSonics, Toronto, ON, Canada) having a center frequency of 40 MHz. The transducer’s specified axial and lateral resolutions were 40 µm and 90 µm, respectively. Acoustic coupling was achieved using a hydrogel pad [31] fixed and covered by a transparent sheet (Tegaderm™ Pad Film Dressing; 3M Company, St. Paul, MN, USA), with a small amount of Ultrasound transmission gel (Aquasonic® Clear Ultrasound Gel; Parker Laboratories, Inc., Fairfield, NJ, USA) applied between the sheet and the surface of the target tooth to ensure proper acoustic coupling. The transducer was hand-held to manually adjust the B-scan orientation, simulating a clinical scenario; the operator’s hand was braced against the fixed C-clamp holder to enhance stability. While this approach introduces potential operator-dependent variability in transducer positioning and contact pressure, it represents a necessary step towards clinical realism. The scanning protocol focused on capturing a consistent profile that included the two reference steel balls, the enamel surface, the gingiva, and the underlying alveolar bone crest. This approach ensured that the same anatomical cross-section could be compared across different bone loss stages. A voice-command acquisition function integrated into the ultrasound system (Vevo Scan; FUJIFILM VisualSonics, Toronto, ON, Canada) was used to trigger each acquisition to minimize examiner movement. For each scan position, one hundred frames with dimensions of 14 by 14 mm were collected at a frame rate of 51 frames per second. A single, representative frame was then selected for analysis based on objective criteria, including a adequate frame clarity within a defined region of interest (ROI) over the bone crest, and the simultaneous, clear visualization of the CEJ, alveolar bone crest, and both fiducial markers, with a complete absence of motion artifacts. This selected frame was saved in.tiff format for subsequent analysis.

The system was configured in abdominal mode with the following parameters, which were standardized across all acquisitions to ensure comparability: a dynamic range of 58 dB, and an image depth and width of 14 mm to consistently encompass the entire region of interest, including the fiducial markers, enamel surface, and alveolar bone crest. A focal depth of 7 mm was selected to optimize lateral resolution at the estimated average depth of the alveolar bone crest. The gain was set to 0 dB, as this default setting provided optimal contrast for hard tissue interfaces without signal saturation during preliminary testing.

### Measurement techniques

To evaluate alveolar bone loss, we employed five distinct measurement strategies, which can be grouped into two primary categories: Manual measurements by blinded observers and Automated algorithms. These were applied to both the photographic ground truth and the ultrasound images, as summarized in Table 1.

**Table 1:** Summary of measurement methods.

Category	Method name	Acronym	Description	Modality
**Manual**	Manual Blinded Observers – Camera	Manual-BO-CA	Manual tracing by experts using photographic images.	Camera
	Manual Blinded Observers – Ultrasound	Manual-BO-US	Manual tracing by experts using ultrasound images.	Ultrasound
**Automated**	Automatic Camera Analysis	Auto-CA	Automated analysis of photographic images.	Camera
	Direct Tracing Superposition – Ultrasound	DTS-US	Automated, frame-registration-based measurement of bone crest displacement.	Ultrasound
	Auto-SVD-Compounded Ultrasound	Auto-SVD-US	Automated, SVD-enhanced measurement of CEJ-to-ABC distance.	Ultrasound

### Manual blinded observers (Manual-BO-US)

A manual measurement protocol was executed by three blinded examiners (Ex1, Ex2, Ex3) to ensure objective and reliable data collection for both the photographic ground truth and ultrasound images.

The alveolar bone loss for a given stage was calculated as the difference in the position of the ABC relative to a fixed marker between consecutive stages. Examiners measured the distance from a defined point on the superior steel ball to the bone crest. The specific reference point was chosen based on the most reliable and reproducible landmark identifiable in each imaging modality (the center of the steel ball for photography and the top tip for ultrasound):

To obtain measurement by photography, the center of the steel ball was used, determined by manual annotation in the orthogonal photographic view, as this point is unambiguous and invariant.

In ultrasound Analysis, the top tip (the most superficial hyperechoic point) of the steel ball’s cross-sectional profile in the B-scan was used. This point was selected because it represents the first and most consistent acoustic reflection from the steel ball, providing a stable and repeatable landmark in the ultrasound image, whereas the geometric center cannot be reliably determined due to acoustic artifacts like reverberation (false echoes appearing deeper than the true reflector) and shadowing (reduced signal intensity behind highly reflective structures). This modality-specific adaptation is a necessary consideration in multimodal imaging comparisons.

This difference in reference points introduces a fixed, constant offset between the two modalities, equal to the radius of the steel ball (500 µm). Since all analyses are based on the change in distance between consecutive stages, this constant offset is canceled out and does not contribute to the measured bone loss values or the bias reported in the Bland-Altman analysis.

To prevent bias, the examiners were blinded to the bone loss stage and each other’s results. This was achieved by providing each examiner with a fully randomized and anonymized set of images. Images from all teeth (M2, M3, PM4) and all stages were pooled and assigned random codes, ensuring that consecutive stages of the same tooth were not analyzed sequentially. The examiners had no knowledge of the amount of bone removed or previous measurement data.

For photographic analysis, measurements were performed using image analysis software (ImageJ; National Institutes of Health, Bethesda, MD, USA). Examiners manually annotated the defined points (ball center and bone crest) to calculate the distance between the ball and bone crest. Each examiner analyzed four distinct frames per stage, repeating the measurement process three times per frame. For ultrasound image analysis, the same protocol was followed using ultrasound analysis software (Vevo-lab; FUJIFILM VisualSonics, Toronto, ON, Canada) [32]. Examiners were trained to use the software’s built-in digital caliper tool for all linear distance measurements. Examiners were instructed to zoom in on the region of interest for precise point placement, targeting the most apical point of the hyperechoic ABC and the top tip of the superior steel ball’s profile for each measurement. Examiners were given explicit guidelines for identifying the reference steel ball tip and the bone crest signal, which appears as a distinct, strong, and spatially coherent reflection. They were instructed to disregard isolated speckle artifacts, which present as transient, less organized, and typically weaker point-like reflections.

**Fig 1:**
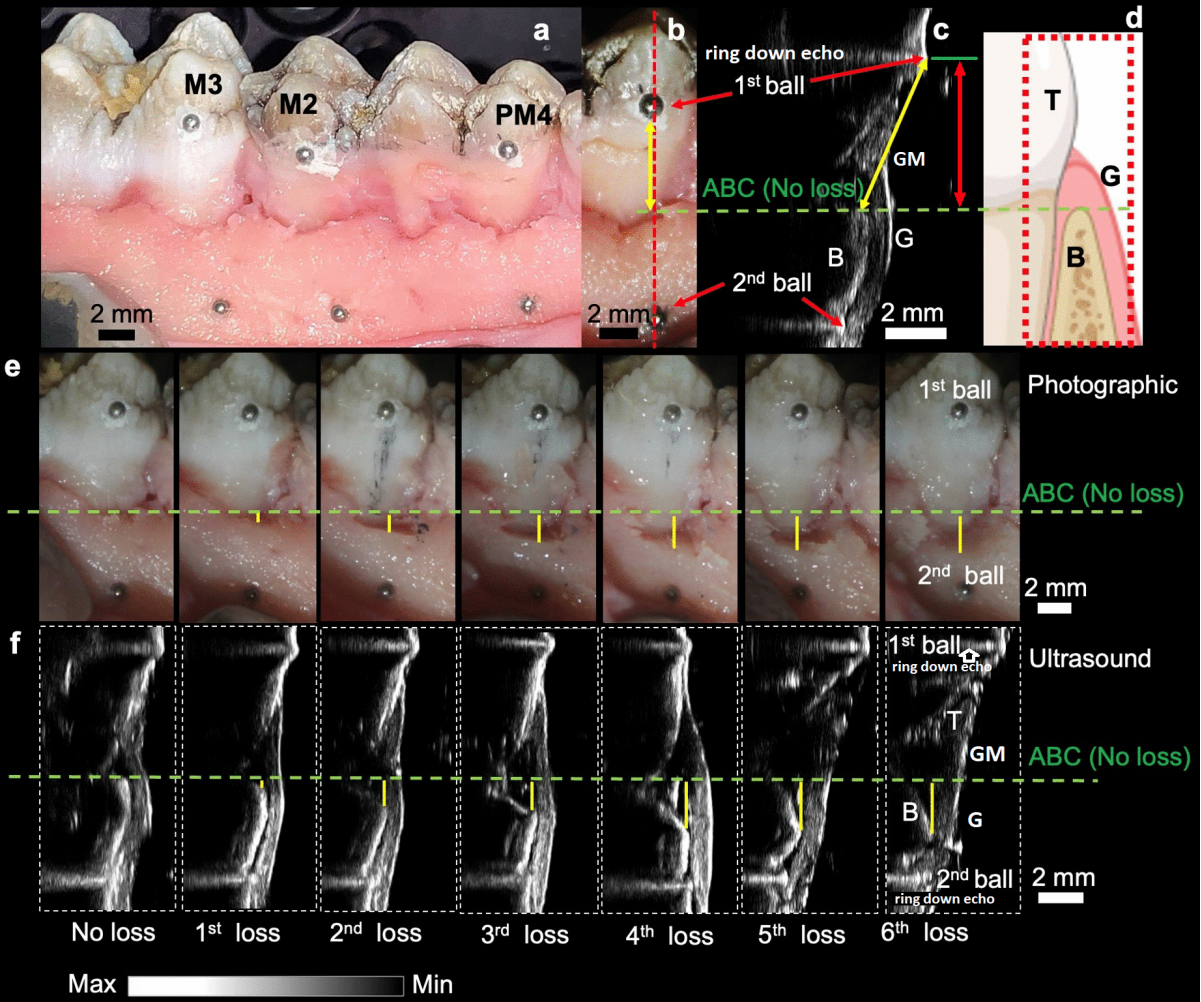
Visualization of alveolar bone crest using photography and ultrasound. **(a)** A normal view of the Canon camera captures a fixed pig jaw. The alveolar bone was exposed by reflecting the overlying gingiva. Two steel balls were fixed on each tooth site as fiducial markers. **(b)** A cropped image section frame of tooth PM4 under photographic camera showing the yellow double arrow between the 1^st^ steel ball and the bone crest aligned with the dashed red line which is the tested comparable plane between the two fixed balls, **(c)** The corresponding ultrasound frame at B-mode section frame scanned by Vevo F2 with a 40 MHz ultrasound transducer, the distance of yellow double arrow between the 1^st^ steel ball and the bone crest ABC was measured. The distance of the red double arrow in **(c)** is corresponding with the distance of the yellow double arrow in **(b)**. The fixed steel ball can be distinguished by a ring down echo as shown clearly at the ultrasound scan. **(d)** is a schematic diagram of a cross-section profile with no bone loss. Panels **(e)** and **(f)** represent the alveolar bone crest of tooth M3 following six stages of alveolar bone mechanical removal. **(e)** Photographic frames captured by a Canon camera. **(f)** Ultrasound scanning using a 40 MHz ultrasound array transducer, where the green dashed line is the alveolar bone crest level before bone removal. ABC (No loss): alveolar bone crest level with no bone loss, T: tooth enamel, B: alveolar bone, gingiva margin GM, and G: gingiva. The length of the yellow lines at panels **(e)** and **(f)** is the change in the amount of bone level detected at different alveolar bone loss stages. The fixed steel balls can be distinguished in panel **(f)** by a ring down echo as shown clearly at the ultrasound scan.

This replication resulted in 36 measurements per stage per modality (4 frames × 3 repetitions × 3 examiners). The values from all replicates were averaged per examiner for subsequent analysis. Representative examples at tooth PM4 and M3 of the ABC visualization and the manual measurement protocol for both photography and ultrasound are provided in Fig 1.

### Automated measurement algorithms

**Fig 2:**
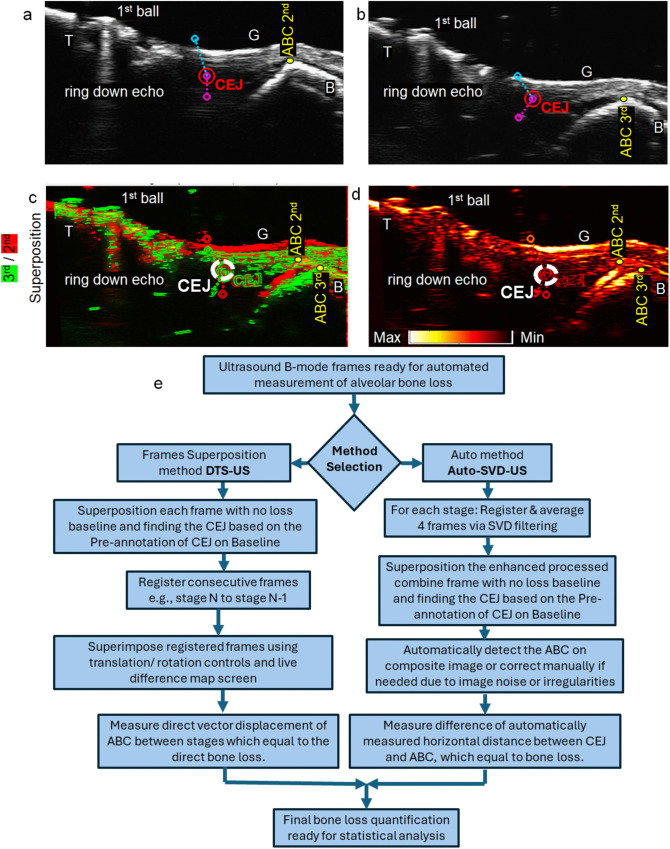
Ultrasound-based assessment of alveolar bone loss using DTS-US method (at molar M2). Panels **(a)** and **(b)** represent the raw ultrasound frames at the 2nd and 3rd loss stages, the fixed steel ball can be distinguished by a ring down echo as shown clearly at the ultrasound scan. Panels **(c)** and **(d)** represent the frame superposition method. Consecutive frames are overlaid to measure the displacement of the alveolar bone crest (ABC) between both frames. In **(c)**, red/green overlays demonstrate frame registration and displacement tracking across stages (2nd and 3rd loss stages). The tooth enamel surface and cemento-enamel junction “CEJ” coincide, while the shift between ABCs directly quantifies bone loss measurement. In **(d)**, the raw intensity difference map between consecutive frames confirms optimal overlay adjustment and highlights the ABC displacement. The gingiva surface shows high intensity due to different separated gingiva positions at different stages and the pressure force difference of the handheld transducer on the gingiva surface. Panel **(e)** shows a flowchart of the two quantification methods for bone loss measurement: DTS-US and Auto-SVD-US. Anatomical landmarks: gingiva “G”, gingiva margin tooth “GM”, enamel “T”, alveolar bone “B”, cemento-enamel junction “CEJ”, in red at raw images **(a)** and **(b)**, and in white at superposition images **(c)** and **(d)**, alveolar bone crest “ABC” in yellow.

Two complementary automated algorithms were developed using technical computing software (MATLAB R2023a; MathWorks, Natick, MA, USA) [33] to provide objective and precise quantification of alveolar bone loss (S1 Code). Both methods utilize the CEJ as a stable anatomical reference point, offering a more clinically relevant framework than external markers such as the steel balls used in this study for the blinded observers’ method. Rather than relying on a deep learning-based approach, which requires many annotated image datasets, we developed a distinct methodology by optimizing traditional image processing techniques for 40 MHz HFUS data. This approach offers transparency and ease of use, making it suitable for our proof-of-concept study. This included the application of Singular Value Decomposition (SVD) filtering for noise reduction [34,35] and intensity-based registration for alignment, which are particularly suited to address the challenges of speckle noise and subtle hard-tissue interfaces characteristic of this modality. All algorithms were implemented using technical computing software and its image processing toolbox (MATLAB R2023a with Image Processing Toolbox; MathWorks, Natick, MA, USA) [33]. Fig 2 e shows a flowchart of the two algorithms for measuring the alveolar bone by HFUS, the methods are described in detail in the following sections.

**Method 1: Direct tracing superposition (DTS-US)**

This method quantified bone loss by directly measuring the displacement of the ABC between two consecutively acquired stages through image registration and subtraction. Fig 2 a-d shows an example of applying DTS-US on a molar M2 between 2^nd^ and 3^rd^ bone loss stages for measuring the direct shift of ABCs, which is considered as a bone loss between 2^nd^ and 3^rd^ stages.

The method as shown in (S1 Code) has the following processing steps for (DTS-US):

### One-Time CEJ Annotation

On the baseline image (No bone loss stage), the CEJ point was manually annotated by an expert observer (TA) to serve as a fixed anatomical reference. This CEJ and enamel surface are used as landmarks for the superposition of approximately the same position frames, ensuring a reliable starting point.

### Interactive Alignment and Manual Measurement

A semi-automated workflow began with initial automatic alignment using edge-based registration to optimize enamel surface matching across translation and rotation parameters [36–38]. Following this computational alignment, expert user (TA) interactively refined the registration through real-time sliders for precise translational (±500 pixels) and rotational (±90°) adjustments, supported by live visual feedback from composite overlays and difference maps.

### Superposition and direct ABC displacement vector measurement

After precise registration, the frames were superimposed. The bone loss was quantified by automatically computing the displacement vector of the ABC. This was achieved by first generating a difference map from the superimposed images. The ABC in the previous stage (N-1) was used as a reference point, and its new position in the current stage (N) was identified as the peak of the intensity difference profile along a direction normal to the enamel surface. The magnitude of this vector represented the bone change between the two stages.

**Method 2: Auto-SVD-compounded ultrasound measurement (Auto-SVD-US)**

**Fig 3:**
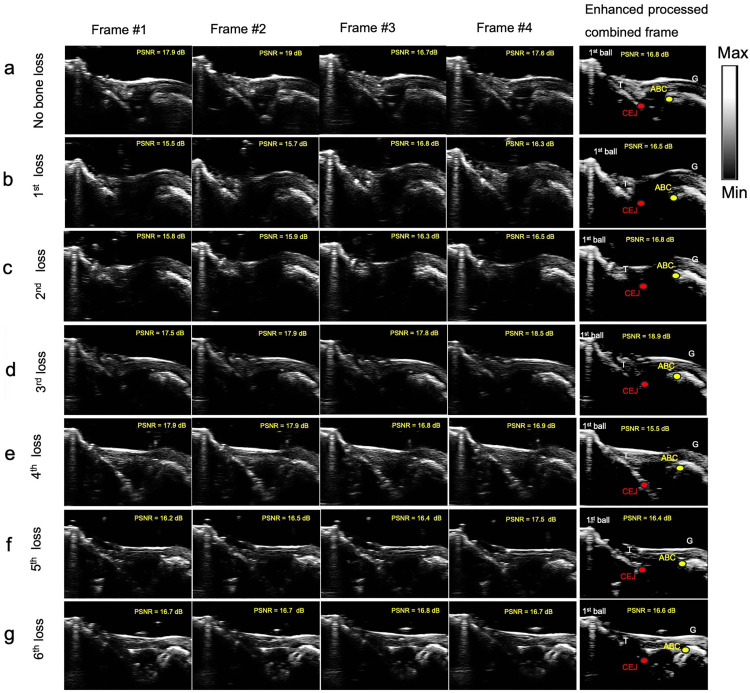
Ultrasound-based assessment of alveolar bone loss using image quantification method Auto-SVD-US at premolar PM4. Image rows at **(a-g)** representative application of the automated method across staged bone loss defects: (no bone loss to 6^th^ loss) respectively. Four sequential B-mode ultrasound frames were collected (Frame#1 to #4), with the last column showing the comparable enhanced combined frame after SVD filtering. CEJ coordinates were obtained by overlaying each stage with the baseline (no-loss) frame, and ABC was automatically detected. The horizontal CEJ-ABC distance was then measured at each stage to quantify bone loss. Anatomical landmarks: gingiva **(G)**, tooth enamel **(T)**, alveolar bone **(B)**, Cemento-enamel junction (CEJ, red), alveolar bone crest (ABC, yellow). Anatomical landmarks: gingiva **(G)**, tooth enamel **(T)**, alveolar bone **(B)**, Cemento-enamel junction (CEJ, red), alveolar bone crest (ABC, yellow).

The Auto-SVD-US method was developed to measure alveolar bone loss by leveraging multi-frame compounding and noise suppression. This method uses Singular Value Decomposition (SVD) [34,35] to create a single, enhanced image from four frames acquired at different time points for every bone loss stage. Bone loss was calculated as the change in the absolute distance from the CEJ to ABC between consecutive stages. Fig 3 shows an example of applying Auto-SVD-US on the premolar PM4, from no bone loss to 6^th^ bone loss stage and shows the comparably enhanced frame after the SVD combined filter at each stage over the raw collected frames.

The method as shown in (S1 Code) has the following processing steps for (Auto-SVD-US):

### Multi-frame acquisition and preprocessing

For each examination stage, four sequential ultrasound frames were acquired from an approximately identical region of interest (ROI) centered on the target tooth, acknowledging the practical constraint of perfect repositioning. All frames underwent manual ROI selection to ensure consistent anatomical coverage. The system standardized frame dimensions through an automated center-cropping and zero-padding algorithm to maintain spatial consistency. Each frame was converted to double-precision format, normalized, and any non-finite values were handled through zero-replacement to ensure mathematical integrity for subsequent processing.

### Rigid registration and frame alignment

To establish a common spatial framework, an intensity-based rigid registration was performed. The first frame served as the reference. Using a mean squares metric and a regular step gradient descent optimizer (300 maximum iterations), each subsequent frame was transformed to align with the reference. The selection of 300 iterations was empirically determined to balance computational efficiency and processing time with registration accuracy, ensuring robust convergence across diverse image qualities. This step corrected for global translational and rotational shifts, ensuring that the alveolar bone structures were spatially congruent across all four frames before compounding.

### SVD filter for noise suppression and image compounding

To suppress uncorrelated noise and enhance the coherent topographic signal, the stack of four registered frames was processed using Singular Value Decomposition (SVD) [34,35]. The procedure was as follows: First, the image stack was reshaped into a two-dimensional matrix A of dimensions (pixels) × (frames). Specifically, with an image resolution of h (height) × w (width) and four frames, the matrix A was constructed by vectorizing each frame, resulting in dimensions of (h × w) × 4. Each column of A represented a single frame vectorized into a one-dimensional array.

This matrix was then decomposed using an economy-size SVD, factorizing it into three constituent matrices:


$$\begin{array}{c} \text{A}=\text{U }\Sigma \;{\text{V}}^{\text{T}} \end{array}$$

Where: U is an (h × w) × 4 orthogonal matrix whose columns represent spatial components, or eigen images, of the input data. Σ is a 4 × 4 diagonal matrix containing the singular values ($\sigma_{\text{1}}$ to $\sigma_{\text{4}}$) in descending order. These values quantify the energy or significance of each corresponding spatial component in U. ${\text{V}}^{\text{T}}$ is a 4 × 4 orthogonal matrix whose rows represent the temporal components, describing how the spatial modes are weighted across the different frames.

A low-rank approximation was applied to filter the data. This was achieved by retaining only the most significant singular values, which correspond to the coherent bone topography, while discarding lower-value components associated with random noise and residual artifacts. For this analysis, the singular value matrix was truncated to retain only the first two components, preserving the primary spatial signal of the anatomy while effectively suppressing noise-dominated higher-order components.

The filtered matrix ${\text{A}}^{\prime }$ was reconstructed via ${\text{A}}^{\prime }=\text{U}*\Sigma^{\prime }*{\text{V}}^{\text{T}}$. where $\Sigma^{\prime }\;$ represents the truncated singular value matrix. Finally, this matrix was reshaped back into its original spatial dimensions (h × w). The final, SVD-compounded image for the stage was extracted as the first spatial component (the first column of U), which provides the purest representation of the bone topography with effectively suppressed noise.

In ultrasound imaging, speckle noise is largely uncorrelated between frames, whereas structural anatomical features such as bone and enamel interfaces remain spatially coherent; therefore, SVD allows separation of coherent anatomical structure from incoherent speckle clutter through low-rank approximation.

**Automated landmark identification and distance measurement**

The SVD-compounded image for each stage was used for quantitative analysis. Key anatomical landmarks, the CEJ, ABC, and a steel ball reference marker were automatically detected using intensity gradient analysis and surface fitting algorithms. The tooth surface was traced using adaptive thresholding. The absolute distance from the CEJ to the ABC was automatically computed in pixel coordinates and converted to physical dimensions (micrometers) using a calibrated pixel size of 18.24 µm.

**Longitudinal bone loss quantification**

The entire previous process (steps 1−4) as repeated independently for each consecutive bone removal stage (e.g., Stage N and Stage N-1), resulting in two stable, high-quality measurements. The bone loss between two consecutive stages was then computed as the difference in the CEJ-to-ABC distances:


$$\begin{array}{c} \text{Bone loss}(\Delta )=(\text{CEJ}-\text{to}-\text{ABC Distance at Stage N})-(\text{CEJ}-\text{to}-\text{ABC Distance Stage N}-\text{1}) \end{array}$$

A positive Δ value indicates bone loss, as the CEJ-to-ABC distance increases, while a negative value would suggest a measurement anomaly. This approach condenses the analysis from 4 potential cross-comparisons (4 frames vs. 4 frames) to a single, highly reliable comparison between two SVD-enhanced consensus images, dramatically improving efficiency and precision.

**Interactive visualization and data export**

The system provided multi-modal visualization with overlays of tissue boundaries, landmarks, and measurement lines. All data, including processed images, measurement results, and analysis parameters, were exported to standardized formats, including spreadsheet files (Microsoft Excel; Microsoft Corporation, Redmond, WA, USA), MATLAB files (MathWorks, Natick, MA, USA), and high-resolution image files for documentation and further analysis.

In the Auto-SVD-US algorithm, several parameters were fixed across all datasets, including the SVD threshold selection range, filtering window size, and CEJ detection parameters. User-defined inputs were limited to region-of-interest selection and image cropping. Parameter values were selected based on preliminary testing to maximize signal stability and boundary detection accuracy. The MATLAB implementation and processing workflow will be made available upon request to support reproducibility (S1 Code at supporting Information).

In summary, Method 1 (DTS-US) provided a direct measurement of bone crest displacement through consecutive image subtraction, ideal for visualizing incremental changes. Method 2 (Auto-SVD-US) offered a highly efficient and automated workflow by leveraging multi-frame averaging and absolute distance measurements. The congruence of results from these two independents, CEJ-based methods provided robust validation for the bone loss measurements using high-frequency ultrasound.

### Quantitative image quality assessment and landmark identification

To objectively evaluate the enhancement provided by the SVD-compounding algorithm, the Signal-to-Noise Ratio (SNR) was calculated for each individual frame and the final compounded result. The SNR was defined as 20·log10 (Mean_Signal/ Std_Noise). The signal region was defined as the central area of the image, spanning from 25% to 75% of its height and width to encompass the alveolar bone. To ensure robustness, pixels with intensities below 1% of the frame’s maximum value were excluded from the calculations. For the automated measurement of bone loss, the Cemento-Enamel Junction (CEJ) was established as a fixed reference point on the baseline (no bone loss) SVD-compounded image. This coordinate was then propagated to all subsequent bone loss stages via intensity-based rigid registration, which aligned the enamel surfaces across time points. This strategy ensured a stable and consistent anatomical reference, making the measurement robust to the intermittent invisibility of the CEJ in individual B-mode frames due to acoustic shadowing.

### Data and statistical analysis

#### Definition of system error.

The inherent precision of each measurement system, defined as the system error, was determined from repeated measurements under unchanged conditions at the baseline (“no loss”) stage. For each method, the apparent “bone loss” was calculated between replicate measurements where no anatomical changes occurred. The system error was calculated as the mean of these apparent changes plus two standard deviations (Mean + 2SD), establishing a one-sided upper threshold for measurement noise corresponding to the 97.5th percentile under a normal distribution [39]. Baseline measurement error distributions were visually inspected using histograms and were found to be approximately normally distributed; therefore, the Mean + 2SD threshold criterion was considered appropriate. Since bone loss is a unidirectional process, this one-sided threshold was used as the statistical criterion, and any measured change in subsequent stages exceeding this threshold was considered a true-positive detection of bone loss.

For clarity, three related statistical concepts were defined in this study. System error represents the inherent variability of a measurement method under unchanged conditions and defines the noise floor of the measurement system. The minimum detectable change (MDC) represents the smallest change that can be distinguished from measurement variability with statistical confidence and is a theoretical statistical threshold derived from system variability. In contrast, the empirical detection threshold was determined experimentally as the smallest actual bone loss increment that was consistently detected above the system error threshold across incremental bone loss stages. Any measured change in subsequent stages exceeding this threshold was considered a true-positive detection of bone loss.

The number of measurements (N) used to calculate the system error differed by method, reflecting their distinct operational workflows:

Manual-BO-US: N = 180. This represents the total number of manual annotations performed by the three blinded examiners on the baseline frames. The apparent change was calculated as the difference between each individual measurement and the shortest measurement at baseline.

DTS-US: N = 20. This method quantifies the change between two frames. The system error was calculated from all possible pairwise comparisons between the four ultrasound scans acquired at each of the five independent baseline events.

Auto-SVD-US: N = 20. This method generates a single denoised composite image per independent baseline acquisition event. The system error was calculated as the Mean + 2SD of the distribution of 20 absolute CEJ-to-ABC distance values measured from these baseline composites.

Baseline measurement error distributions were visually inspected using histograms and were found to be approximately normally distributed; therefore, the Mean + 2SD threshold criterion was considered appropriate. Confidence intervals for sensitivity and specificity were calculated where applicable; however, due to the limited number of independent bone loss events, these intervals may be wide and should be interpreted cautiously.

### Statistical analysis

Agreement between ultrasound measurements and photographic measurements was evaluated using Bland–Altman analysis, including mean bias and limits of agreement. Reliability between measurement methods and observers was assessed using intraclass correlation coefficients (ICC). Linear regression analysis was performed to evaluate the relationship between ultrasound and photographic measurements.

Detection capability was evaluated using sensitivity analysis at each bone loss increment stage. A measurement was considered a true-positive detection when the measured bone loss exceeded the system error threshold. Detection sensitivity was calculated as the proportion of true-positive detections at each bone loss increment stage.

Statistical significance was defined as *P* < 0.05. All statistical analyses were performed using MATLAB (MathWorks, Natick, MA, USA).

### Agreement and reliability analysis

Bland-Altman analysis was employed to assess the agreement between measurement methods through a series of pairwise comparisons. The limits of agreement (LOA) for each comparison were calculated as the mean difference (bias) ± 1.96 times the standard deviation of the differences.

Reliability was assessed using intraclass correlation coefficients (ICC), calculated from a two-way random-effects analysis of variance (ANOVA). The analyses were performed separately for each tooth (M3, M2, PM4). To evaluate the consistency between different measurement techniques, the ICC(2,1) model for absolute agreement among single measures was applied to two sets of comparisons: first, among all methods [Auto-SVD-US, DTS-US, Manual-BO-US, Auto-CA, Manual-BO-CA], and second, among the ultrasound methods exclusively [Auto-SVD-US, DTS-US, Manual-BO-US]. To quantify inter-observer variability for the manual measurements, the ICC(2,1) model for absolute agreement among single measures was also used separately for the manual photographic data [Manual-BO-CA_Ex1, Manual-BO-CA _Ex2, Manual-BO-CA_Ex3] and the manual ultrasound data [Manual-BO-US_Ex1, Manual-BO-US_Ex2, Manual-BO-US_Ex3]. ICC values were interpreted as follows: < 0.50 poor, 0.50–0.75 moderate, 0.75–0.90 good, and >0.90 excellent reliability. Due to the limited number of independent experimental units inherent to this proof-of-concept design, formal normality testing was not systematically performed. Instead, baseline error distributions were visually inspected and found to be approximately normal. Accordingly, ANOVA- and ICC-based analyses were applied as measures of internal consistency and reliability within a repeated-measurement framework rather than for population-level inference.

### Detection of bone level changes and diagnostic accuracy

The diagnostic capability of ultrasound to detect the presence of bone loss was assessed by calculating sensitivity and specificity. A measured change was classified as a true positive only if it exceeded the method-specific system error threshold. Changes within this noise range were considered false positives.

Sensitivity was calculated as the proportion of actual bone loss stages (as defined by the ground truth) that were correctly identified. Specificity was calculated as the proportion of no-bone-loss stages (pairwise comparisons at baseline) that were correctly identified as such. The system error (Mean + 2SD of baseline noise) establishes the threshold for a single measurement to be considered a real change [40].

## Results

The *ex vivo* bone loss model was generated in sequential stages, comprising six stages of bone loss plus one control stage for teeth M3 and PM4 and four stages plus two control stages for tooth M2. No datasets were excluded from the analysis, and all acquired images and measurements were included in the statistical evaluation. The precision of detecting changes was verified using photographic ground truth images, yielding a mean incremental bone loss of 215 ± 35 µm (range: 180–250 µm) per stage.

Statistical comparisons between measurement methods were performed using regression analysis, intraclass correlation coefficient (ICC) analysis, and Bland–Altman agreement analysis. A significance level of *P* < 0.05 was used.

### Validation of the ground truth and model

**Fig 4:**
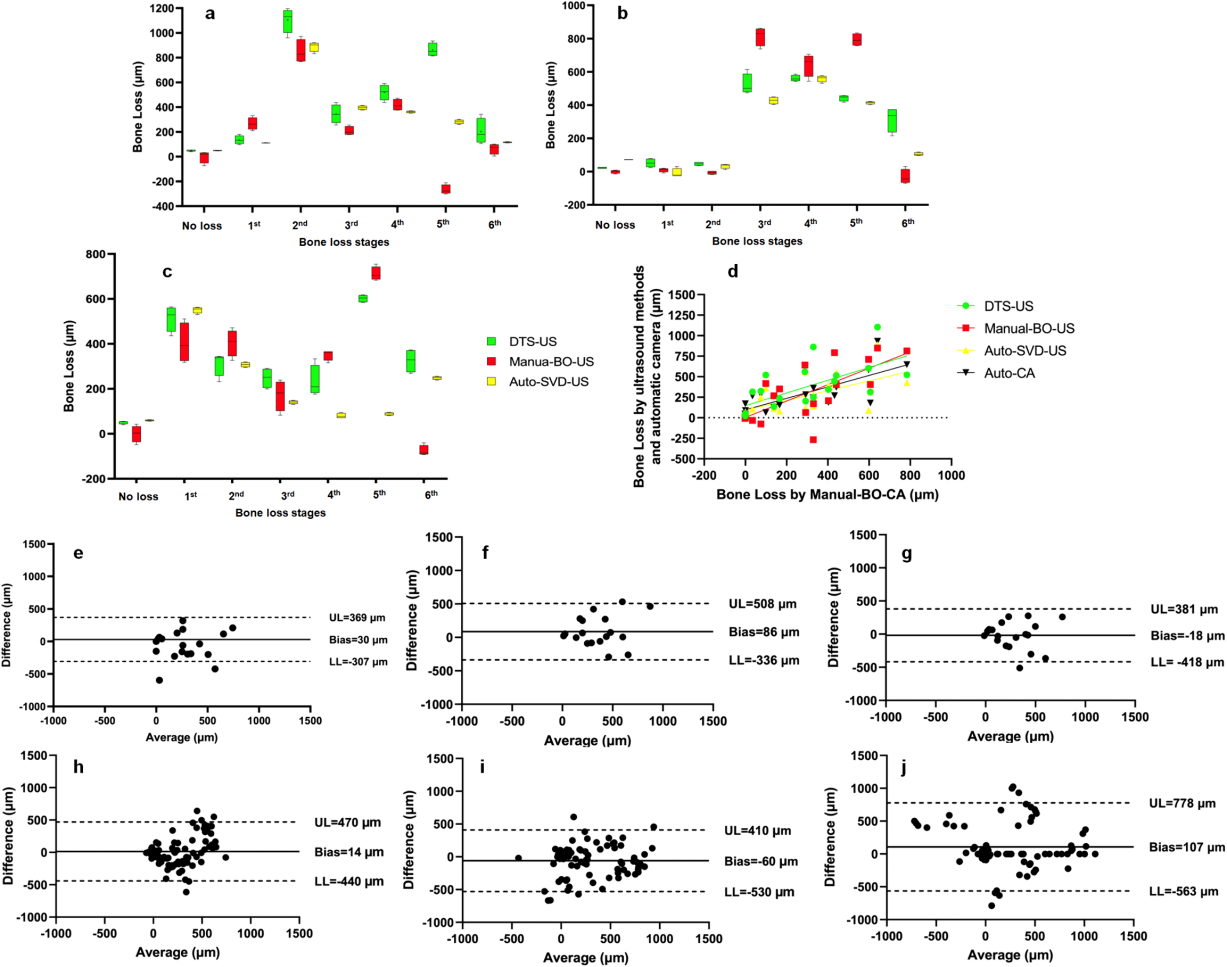
Comparisons between ultrasound measurement methods of alveolar bone loss and camera measurement methods. Panels **(a-c)** show scatter plots of different ultrasound methods used at teeth M3, M2, and PM4, respectively (DTS-US: direct tracing superposition; Manual-BO-US: manual measurement by blinded examiners; Auto-SVD-US: automatic method after SVD filter). Panel **(d)** shows regression fits for the ultrasound methods and the automated camera method (Auto-CA) plotted against the manual camera ground truth (Manual-BO-CA). Panels **(e-g)** show Bland-Altman analysis between the ultrasound measurement methods. compared with Manual-BO-CA. Panels **(h-j)** show Bland-Altman analysis for each manual ultrasound examiner (Ex1, Ex2, and Ex3 respectively) compared with Manual-BO-CA. UL: Upper LOA, LL: Lower LOA.

Fig 4, panels (a, b, and c) show scatter plots comparing three distinct ultrasound measurement methods for representative teeth (M3, M2, PM4) across all bone loss stages: manual measurements by blinded observers (Manual-BO-US), registered frame comparison (DTS-US), and the automated SVD-based method (Auto-SVD-US). Panel (d) shows regression lines comparing all ultrasound methods and the automated photographic method (Auto-CA) against the ground truth (Manual-BO-CA). The data plots reveal that all methods track progressive bone loss with high consistency, showing minimal divergence, clear separation between consecutive stages, and similar response patterns.

Regression analysis demonstrated significant correlations between ultrasound measurements and the photographic ground truth. Manual-BO-US showed the strongest correlation (R² = 0.55, *P* = 0.0001), followed by Auto-CA (R² = 0.53, *P* = 0.0002), DTS-US (R² = 0.46, *P* = 0.0008), and Auto-SVD-US (R² = 0.41, *P* = 0.0016). These results confirm that the ultrasound methods were able to track incremental bone loss consistently across all stages.

### Performance of measurement methods: system error and reliability

The precision and reliability of all five measurement methods were systematically evaluated. System error, representing the inherent measurement variability, was calculated from repeated baseline measurements, while intraclass correlation coefficients (ICC) assessed reliability across different comparisons.

As shown in Table 2, DTS-US showed the lowest system error (77 µm) among the ultrasound measurement methods, while Manual-BO-US exhibited the highest variability (113 µm).

**Table 2:** System error thresholds for different measurement methods (Mean + 2SD of baseline measurement noise).

Measurement method	System error (Mean + 2SD, µm)
**Manual-BO-CA**	81
**Auto-CA**	94
**Manual-BO-US**	113
**DTS-US**	77
**Auto-SVD-US**	111

ICC values revealed excellent reliability across all comparisons (Table 3). Inter-observer reliability was outstanding for both manual measurements by blind observers using Manual-BO-CA (ICC = 0.92) and Manual-BO-US (ICC = 0.98). Inter-method reliability among all five methods of ultrasound and camera ranged from ICC = 0.89 to 0.99 across different teeth. Inter-method reliability among all three ultrasound methods ranged from ICC = 0.86 to 0.98 across different teeth. The high ICC values across all comparisons, combined with low system errors, confirm that all measurement methods provide reliable and consistent results, with automated methods performing comparably to manual ground truth measurements.

**Table 3:** Intraclass correlation coefficient (ICC) for reliability analysis.

Comparison	ICC Value	95% Confidence interval	Interpretation
**Inter-Observer (**Manual-BO-CA)	0.92	[0.88, 0.95]	Excellent
**Inter-Observer (**Manual-BO-US)	0.98	[0.97, 0.98]	Excellent
**Inter-Method (All 5 Methods) at tooth (M3)**	0.89	[0.82, 0.95]	Good
**Inter-Method (All 5 Methods) at tooth (M2)**	0.99	[0.991, 0.997]	Excellent
**Inter-Method (All 5 Methods) at tooth (PM4)**	0.92	[0.89, 0.96]	Excellent
**Inter-Method (Ultrasound Methods) at tooth (M3)**	0.86	[0.75, 0.96]	Good
**Inter-Method (Ultrasound Methods) at tooth (M2)**	0.98	[0.96, 0.99]	Excellent
**Inter-Method (Ultrasound Methods) at tooth (PM4)**	0.90	[0.82, 0.97]	Excellent

### Agreement between ultrasound and photographic ground truth

The agreement between ultrasound-based measurements and the photographic ground truth was comprehensively assessed using Bland-Altman analysis across all measurement scenarios and examiner pairs.

### Primary method comparisons

Across all modalities, ultrasound-based methods showed agreement with camera-based measurements (S1 Table), as illustrated in Fig 4. Panels (e-g) show Bland-Altman plots for the three primary ultrasound methods (Manual-BO-US, DTS-US, Auto-SVD-US) compared to the manual camera ground truth, while panels (h-j) show the same comparison broken down by individual examiner for the Manual-BO-US method. The Auto-SVD-US vs Manual-BO-CA comparison demonstrated the smallest bias was –18 µm, indicating no systematic over- or under-estimation. The best-case bias of −18 µm corresponds to less than 10% error relative to a typical 200 µm bone change increment, indicating clinically acceptable agreement for longitudinal monitoring applications. However, the limits of agreement were −418 µm to 381 µm, representing a range of 799 µm. This wide range indicates that for any single measurement, the difference between this automated ultrasound method and the photographic standard could be substantial. The DTS-US vs Manual-BO-CA comparison showed a slightly larger bias of 86 µm, with limits of agreement between –336 µm to 508 µm, with a range of 844 µm. The Manual-BO-US vs Manual-BO-CA comparison showed a bias of 30 µm corresponds to 15% error relative to a typical 200 µm bone change increment and limits of agreement between –307 µm to 369 µm, with a range of 676 µm.

### Inter-observer and cross-modal variability

S1 Table further provides crucial insights into variability through inter-observer and cross-modal comparisons. The inter-observer agreement for manual ultrasound measurements (Inter-Observer US) showed excellent consistency, with biases between examiners as low as 17 µm and 24 µm corresponds to 8.5% and 12% error relative to a typical 200 µm bone change increment. A particularly telling result is found in the cross-modal comparisons for each examiner (Ultrasound vs Camera (Manual blind examiners)). For Examiner 2, the bias was minimal of −60 µm corresponds to less than 30% error relative to a typical 200 µm bone change increment with the tightest limits of agreement between −530–410 µm among the three examiners. In contrast, the inter-observer agreement for the photographic ground truth itself (Inter-Observer CA) showed wider variability, with a bias of −127 µm between Examiners 2 and 3 corresponds to less than 63.5% error relative to a typical 200 µm bone change increment. This indicates that a portion of the disagreement between ultrasound and camera can be attributed to the inherent challenges of manual landmark identification in both modalities, rather than to the ultrasound technology alone.

An important observation from S1 Table is the comparison among the ultrasound methods themselves. For instance, the DTS-US vs Auto-SVD-US comparison shows a bias of 105 µm corresponds to 52.5% error relative to a typical 200 µm bone change increment, indicating a systematic difference between these two automated techniques. However, the limits of agreement for this comparison between –225 µm to 435 µm are notably tighter than those observed when comparing ultrasound to the camera ground truth. This suggests that while different ultrasound algorithms may produce offset values, they are consistent with each other, reinforcing the internal reliability of the HFUS modality.

### Detection of bone level changes and diagnostic accuracy

The diagnostic performance of the three ultrasound methods was evaluated by calculating sensitivity and specificity for detecting bone loss at each stage, with results summarized in Table 4.

**Table 4:** Diagnostic accuracy of ultrasound measurement methods.

Measurement Method	Sensitivity (%)	Specificity (%)
**Manual-BO-US**	79	92
**DTS-US**	100	70
**Auto-SVD-US**	82	70

Sensitivity was calculated for each tooth and bone loss stage, with values ranging from 33% to 100% across methods and stages. The DTS-US Frame method achieved the highest overall sensitivity (100%) for detecting bone loss at all stages across all teeth. The Manual-BO-US method showed more variable performance, with sensitivity ranging from 33% to 100%. The Auto-SVD-US method demonstrated high sensitivity (100%) for most stages, with slightly lower performance at smaller bone loss increments.

Specificity was calculated from pairwise comparisons between replicate frames at the baseline stage, representing the ability to correctly identify no bone loss. The Manual-BO-US method showed the highest specificity (92%), followed by DTS-US and Auto-SVD-US of (70%). The minimum detectable change (MDC) was calculated for each method and tooth, as detailed in Table 5. The minimum detectable change (MDC) was calculated based on measurement variability and represents the smallest change that can be detected beyond measurement noise with statistical confidence, typically calculated as 1.96 × √2 × SD.

**Table 5:** Detection sensitivity of ultrasound methods at different bone loss increments and determination of the empirical detection threshold of (138 µm).

Tooth/Stage Difference	The average amount of bone removal between stages determined by photography (µm)	Manual-BO-US (%)	DTS-US (%)	Auto-SVD-US (%)
M3/4^th^ −3^rd^	99	67	100	100
M3/1^st^ -No loss	**138**	**100**	**100**	**100**
PM4/4^th^ −3^rd^	166	69	100	100
M2/4^th^ −3^rd^	289	97	100	100
M3/6^th^ −5^th^	292	33	100	100
M3/5^th^ −4^th^	329	44	100	100
PM4/3^rd^ −2^nd^	330	61	100	100
M3/3^rd^ −2^nd^	403	86	100	100
M2/5^th^ −4^th^	432	97	100	100
PM4/1^st^ -No loss	441	100	100	100
PM4/5^th^ −4^th^	598	100	100	100
PM4/2^nd^ - 1^st^	606	100	100	100
M3/2^nd^ −1^st^	641	100	100	100
M2/3^rd^-2^nd^	784	97	100	100

**Note:** The empirical detection threshold (~138 µm) represents the smallest actual bone loss increment that was consistently detected above the system error threshold across measurement methods. This value differs from the theoretical minimum detectable change (MDC), which is calculated from measurement variability, and instead reflects experimental detection performance.

### Clinically reliable detection threshold

The system error, representing the inherent precision of each method, established a fundamental detection limit for alveolar bone loss, ranging from 77 to 113 µm for the ultrasound methods (Table 2), indicating that changes smaller than these thresholds cannot be reliably distinguished from measurement noise. This capability was experimentally confirmed by the consistent detection of a small but real bone loss increment. At the M3/1st – No loss stage, which involved an average bone loss of 138 µm as measured by the photographic ground truth, all three ultrasound methods successfully detected this change with 100% sensitivity (Table 5). Because this amount exceeded the system error of all methods, it represents a practical detection threshold for the HFUS system. The minimum detectable change (MDC) represents a theoretical statistical threshold derived from measurement variability, whereas the 138 µm value represents the empirically observed minimum bone loss increment that was consistently detectable above the system error threshold across methods.

## Discussion

### Novelty and Contribution to the field

While several previous studies have demonstrated the feasibility of using high-frequency ultrasound for periodontal and peri-implant imaging [13–17], most have focused on visualization and single-time-point measurements of bone levels or bone thickness. The novelty of this study does not lie in demonstrating that ultrasound can image the alveolar bone, but rather in establishing the quantitative measurement performance required for clinical monitoring of periodontal bone loss progression. Specifically, this study quantifies measurement precision, determines the minimum detectable bone loss change, and evaluates the capability of high-frequency ultrasound for longitudinal monitoring of small bone-level changes. This framework shifts the role of HFUS from a purely imaging modality to a quantitative monitoring tool for periodontal disease progression. In periodontal disease management, the detection of progression (i.e., changes over time) is more clinically relevant than the determination of a single absolute bone level. This explains why HFUS, despite limitations in absolute agreement, is particularly well suited as a monitoring tool for longitudinal assessment rather than a replacement for initial radiographic evaluation.

### Methodological novelty and experimental design

In addition, this study addresses an important experimental challenge in ex vivo validation by introducing fiducial steel ball markers to ensure that measurements were obtained from the exact same anatomical location across different bone loss stages, identified by their characteristic ring-down echo in ultrasound images (Figs 1–3). These markers, together with a voice control command on the Vevo system to eliminate vibrations and the use of a hydrogel pad to improve acoustic coupling and image clarity, enabled accurate determination of system error (77–113 µm) and the empirical detection threshold (138 µm), and provided a reliable spatial reference for validating automated measurement methods.

Importantly, the novel automated CEJ-based measurement methods particularly the SVD-based approach were subsequently evaluated without relying on the fiducial markers, demonstrating that automated measurements can achieve comparable precision while using clinically relevant anatomical landmarks. This distinction is important because the fiducial markers were used only for experimental validation, whereas clinical measurements would rely on anatomical landmarks such as the cementoenamel junction and the enamel surface.

Together, these contributions establish a quantitative framework for evaluating high-frequency ultrasound as a tool for longitudinal periodontal bone loss monitoring.

### Null hypothesis and study goal

The null hypothesis of this study was that high-frequency ultrasound measurements would not differ significantly from photographic reference measurements and would not be capable of detecting small incremental changes in alveolar bone loss. Based on the results demonstrating low system error, strong agreement with photographic measurements, and reliable detection of small bone loss increments, the null hypothesis was rejected.

The goal of this study was not to replace radiography for absolute bone level measurement, but to demonstrate that high-frequency ultrasound can detect small longitudinal changes in alveolar bone loss with high precision and a defined detection threshold.

### Key study questions: precision, accuracy, and detection capability

This study addressed three questions:

Q1. Does high-frequency ultrasound offer a precise and reliable alternative to radiography for monitoring bone loss?

Our systematic evaluation suggests that HFUS is a precise and reliable method for monitoring alveolar bone loss and may serve as a complementary tool to conventional radiographic methods for longitudinal monitoring. The DTS-US method demonstrated the lowest system error of 77 µm and the automated methods performed comparably or superior to manual measurements while being four times faster. The excellent reliability across all methods (ICC = 0.89–0.99) further establishes HFUS as a robust tool for quantitative periodontal assessment.

The reproducibility of measurements deserves further examination beyond the summary ICC values. As detailed in Table 3, inter-observer reliability for manual ultrasound measurements (ICC = 0.98, 95% CI [0.97, 0.98]) was notably higher than for manual photographic measurements (ICC = 0.92, 95% CI [0.88, 0.95]). This finding is noteworthy because it suggests that blinded examiners achieved greater consistency when identifying landmarks in ultrasound images than in photographs a result that may initially seem counterintuitive given the perceived complexity of ultrasound interpretation. One explanation is that the hyperechoic bone crest signal in ultrasound provides a more discrete and unambiguous target for caliper placement compared to the photographic view, where soft tissue remnants or surface irregularities may introduce variability in landmark identification. Inter-method reliability among all five measurement methods ranged from ICC = 0.89 to 0.99 across the three teeth (Table 3), with M2 showing the highest agreement (ICC = 0.99, 95% CI [0.991, 0.997]) and M3 the lowest (ICC = 0.89, 95% CI [0.82, 0.95]). The slightly lower agreement at M3 likely reflects the greater number of bone loss stages (six stages) at this tooth, which introduced more cumulative measurement opportunities for divergence. Among the three ultrasound methods alone, reliability ranged from ICC = 0.86 to 0.98, confirming that the manual, semi-automated, and fully automated approaches produce consistent and interchangeable results for tracking bone loss progression. The combination of low system error (Table 2) and consistently high ICC values across all observer and method comparisons provides strong evidence that the HFUS measurement framework is both precise and reproducible two prerequisites for any longitudinal monitoring tool.

Q2. How accurate are ultrasound measurements compared to the established ground truth?

Ultrasound measurements showed excellent agreement for tracking changes over time, with the Auto-SVD-US method showing the smallest bias of –18 µm corresponds to less than 10% error relative to a typical 200 µm bone change increment. However, a key finding is the distinct performance profile of HFUS: low system error (high precision) for detecting changes, coexisting with wide limits of agreement (lower accuracy) for single, absolute measurements. This discrepancy arises from challenges in manual probe repositioning, landmark identification subjectivity, and speckle noise. This wide range indicates that for any single measurement, the difference between this automated ultrasound method and the photographic standard could be substantial. While absolute measurements may vary due to reference selection, imaging angle, and anatomical variability, the ultrasound measurements demonstrated high precision and repeatability across stages. In periodontal diagnosis, tracking changes over time is often more clinically relevant than a single absolute measurement value. Therefore, the core clinical value of HFUS lies in its superior ability to safely and precisely monitor disease progression rather than providing a single, absolute baseline measurement, supporting its role as a longitudinal monitoring tool for precision periodontal care.

Q3. What is the minimum amount of bone loss that HFUS can reliably detect?

This study establishes that HFUS can reliably detect bone loss increments as small as 138 µm, a threshold that approaches the system’s lateral resolution limit of 90 µm. This threshold is substantially lower than the 870 µm limit reported for conventional radiography [5], highlighting the potential of HFUS for detecting earlier stages of periodontal disease. These findings demonstrate that high-frequency ultrasound can detect clinically relevant bone loss changes well below the detection limits of conventional radiography, supporting its potential for early periodontal disease monitoring. Conventional radiography was not directly evaluated in this study; therefore, comparisons with radiographic detection limits are based on values reported in the literature and should be interpreted cautiously.

### Clinical translation and detection performance

This performance profile directly informs its clinical application. The technology is exceptionally well-suited for longitudinal monitoring, where the change from a patient’s own baseline is the critical metric. Regarding clinical readiness, our automated methods are the clear candidates for translation. As summarized in Table 6, the DTS-US method, with its highest sensitivity and lowest system error, is the gold standard for longitudinal monitoring in research. The Auto-SVD-US method, with its fully automated workflow and low bias, is the strongest candidate for rapid, high-throughput clinical screening and monitoring.

**Table 6:** Comparative summary of alveolar bone loss measurement methods.

Method	Key advantage	Key limitation	Best use case	Clinical readiness & rationale
**Manual-BO-US**	High specificity (92%); establishes human performance baseline.	Time-consuming; high inter-observer variability; highest system error (113 µm).	Lab validation; not suitable for routine clinical use.	**Low:** Prohibitively slow and variable for clinical workflow.
**DTS-US**	**Highest sensitivity (100%); lowest system error (77 µm).** Best for detecting minute changes.	Requires sequential scans; measures displacement, not absolute level.	**Gold-standard for longitudinal monitoring** in research and high-precision tracking.	**High (for monitoring):** Excellent for tracking change, the core clinical task.
**Auto-SVD-US**	**Fully automated; fastest; excellent agreement (lowest bias: −18 µm).**	Slightly lower sensitivity than DTS-US; wide LoA for single measurements.	**Ideal for rapid, high-throughput clinical screening and monitoring.**	**High:** Automated and fast, enabling seamless integration into patient visits.
**Conventional Radiography**	Standard of care; provides absolute anatomical baseline.	Ionizing radiation; insensitive to early/minor bone-level changes.	Initial diagnosis and baseline assessment.	Current **Standard dental clinic care**

The sensitivity–specificity trade-off observed across methods (Table 4) has direct implications for clinical decision-making. The DTS-US method achieved 100% sensitivity but 70% specificity, while the Manual-BO-US method showed 79% sensitivity but the highest specificity of 92%. The Auto-SVD-US method occupied a middle ground with 82% sensitivity and 70% specificity. This trade-off must be interpreted in the context of the clinical consequences of each type of error. In periodontal monitoring, a false negative failing to detect real bone loss progression could result in delayed treatment and irreversible tissue destruction, particularly in patients with aggressive or rapidly progressing disease. A false positive incorrectly identifying bone loss where none has occurred would likely trigger additional follow-up imaging or a shorter recall interval, which, while inconvenient, carries no risk of tissue harm and no ionizing radiation burden when using ultrasound. Given this asymmetry, higher sensitivity should generally be prioritized over specificity in a periodontal monitoring context, supporting the use of DTS-US for research-grade precision and Auto-SVD-US for routine clinical screening. For patients where unnecessary intervention carries greater risk or cost, the higher specificity of manual measurement may be preferred. This performance profile, summarized in Table 6, allows clinicians and researchers to select the most appropriate method based on the specific monitoring scenario and the relative tolerance for false-positive versus false-negative outcomes. The detailed stage-by-stage sensitivity data in Table 5 further illustrate this trade-off: while DTS-US and Auto-SVD-US detected all bone loss stages including the smallest increment of 138 µm, Manual-BO-US sensitivity dropped to 33–44% at certain stages (M3/6th–5th and M3/5th–4th), indicating that manual measurement may miss subtle changes even when cumulative bone loss is substantial.

The empirical detection limit of 138 µm for our HFUS system has profound implications. This limit was slightly higher than the system error range (77–113 µm), which is expected because a true detectable change must exceed the inherent measurement noise of the system. This relationship confirms the internal consistency of the measurement performance analysis and supports the validity of the experimentally determined detection threshold. For longitudinal monitoring, the empirical detection threshold is more clinically meaningful because it reflects the real detection performance of the system rather than a purely statistical estimate. Due to the limited number of independent bone-loss events evaluated in this proof-of-concept study, confidence intervals for sensitivity and specificity would be wide and therefore less precise. Accordingly, these estimates should be interpreted with caution and considered preliminary indicators of detection performance rather than definitive measures of diagnostic accuracy.

### Agreement analysis and the role of precision versus accuracy

The regression analyses demonstrated moderate correlation coefficients (R² ≈ 0.41–0.55) between ultrasound measurements and the photographic ground truth. While these values are statistically significant, they indicate only moderate linear association and reflect variability in measurement dispersion rather than direct agreement between methods. Importantly, R² quantifies the strength of correlation but does not assess measurement agreement or interchangeability.

Despite the moderate R² values, the relatively low mean bias observed in the Bland–Altman analysis indicates that systematic error is limited and that the primary source of variability arises from random measurement scatter rather than consistent over- or underestimation. Ultrasound measurements did not consistently overestimate or underestimate bone levels but instead showed variability around the reference measurements. This distinction explains how moderate correlation can coexist with low bias.

The limits of agreement observed in the Bland–Altman analysis indicate that ultrasound measurements should not be considered interchangeable with photographic reference measurements for absolute bone level determination. However, periodontal disease management is primarily concerned with detecting changes in bone levels over time rather than determining a single absolute measurement. In this context, the low system error (77–113 µm) and the small empirical detection threshold (~138 µm) demonstrate high precision in detecting longitudinal changes above the measurement noise floor.

Therefore, the ability of high-frequency ultrasound to detect small changes relative to baseline with low systematic bias is more clinically relevant than achieving high absolute agreement with a reference method. These findings highlight an important distinction between accuracy and precision: while absolute measurements show variability, the system demonstrates high precision for monitoring change over time. Overall, high-frequency ultrasound is best positioned as a longitudinal monitoring tool for tracking small changes in alveolar bone levels, rather than serving as a direct replacement for radiographic methods used for absolute measurements or initial diagnosis.

The limits of agreement (approximately 700–800 µm) are substantially larger than the empirically determined detection threshold (~138 µm), indicating that while absolute measurements exhibit considerable variability, the system retains high sensitivity for detecting small longitudinal changes above the noise floor. Importantly, clinically meaningful changes in periodontal bone levels are typically on the order of 100–200 µm in longitudinal monitoring, indicating that the detection threshold of ~138 µm falls within this clinically relevant range.

This disparity highlights an important distinction between absolute accuracy and changes detection capability. The relatively wide limits of agreement reflect variability in single-time-point measurements and therefore limit the use of HFUS for precise absolute bone level determination. However, the much smaller detection threshold demonstrates that the system is capable of consistently identifying clinically relevant incremental changes over time.

From a clinical perspective, periodontal disease monitoring primarily relies on detecting progression rather than establishing a single absolute bone level measurement. Importantly, clinical decision-making relies on reproducible quantitative measurements rather than visual estimation alone. While photographic reference images may appear visually precise, clinicians cannot directly measure bone levels by visual inspection, and therefore consistent detection of changes over time is more clinically meaningful than strict agreement with a reference image. In this context, the ability to reliably detect changes exceeding the system error threshold is more meaningful than achieving tight agreement with a reference method. Therefore, despite the wide limits of agreement, the measurement characteristics of HFUS support its role as a longitudinal monitoring tool, where relative changes from baseline are of primary importance.

### Study strengths

A major strength of this study is the use of a controlled ex vivo model with incremental bone removal and precise photographic ground truth measurements, which allowed accurate quantification of system error, agreement, and minimum detectable change. In addition, multiple measurement approaches were evaluated, including manual, semi-automated, and fully automated ultrasound methods, providing a comprehensive comparison of performance, reliability, and measurement precision across different analysis strategies. The study design incorporated repeated measurements, blinded observers, and automated algorithms, enabling robust evaluation of reliability, agreement, and detection performance. Importantly, this study focused not only on measurement accuracy but also on system error, minimum detectable change, and empirical detection thresholds, which are clinically relevant parameters for longitudinal monitoring. This controlled experimental design enabled a systematic evaluation of the performance characteristics of high-frequency ultrasound for detecting and monitoring small longitudinal changes in alveolar bone loss.

### Anatomical considerations and prior work

Previous studies using high-frequency ultrasound for periodontal imaging have demonstrated that ultrasound can identify key anatomical landmarks such as the cementoenamel junction, gingival margin, and alveolar bone crest in both extracted jaws and human subjects [25]. These findings suggest that ultrasound imaging can be applied to periodontal structures beyond simple geometries. However, complex anatomical regions such as interdental spaces and furcation areas may present challenges related to probe positioning, beam angle, acoustic shadowing, and transducer access. Imaging in posterior teeth and complex periodontal defects may therefore require improved probe positioning strategies and the development of dedicated intraoral ultrasound transducers optimized for periodontal imaging. To date, emerging studies have begun to address these experimental challenges [41].

### Automated SVD-based measurement method

In this study, the Auto-SVD-US method was developed to measure alveolar bone loss by leveraging multi-frame compounding and noise suppression to generate a high-fidelity representative image for each examination time point. The core innovation of this method is the use of Singular Value Decomposition (SVD) [34,35] to create a single, enhanced image from multiple approximately acquired frames, thereby mitigating the confounding effects of micro-shaking and speckle noise inherent in clinical ultrasound scanning. This approach addresses a fundamental challenge in longitudinal ultrasound analysis: the inability to achieve perfect probe repositioning, which introduces measurement artifacts that render conventional pairwise frame comparisons noisy and unreliable. By shifting the paradigm from multiple comparisons of raw data to a single, precise comparison between SVD-enhanced consensus images, the method ensures that subtle yet critical changes in bone topography can be detected with superior accuracy and efficiency, providing a stable foundation for tracking periodontal disease progression. A pertinent question that arises in automated image analysis is how the algorithm ensures specificity in identifying true anatomical landmarks amidst ubiquitous image gradients and speckle noise. Our methodology addresses this through a multi-layered processing pipeline, not a simple peak-detection search. The core of this strategy is the application of Singular Value Decomposition (SVD) filtering, which acts as an anatomical clutter filter by preserving only the dominant signal components corresponding to coherent tissue interfaces while suppressing higher-order noise. Furthermore, the search for landmarks like the alveolar bone crest is constrained to anatomically plausible regions defined by the initial CEJ location and the traced enamel surface, effectively ignoring most confounding gradients. Regarding the geometrical definition of bone loss, the algorithm quantifies the CEJ-to-ABC distance based on clinical convention. We deliberately implemented a longitudinal measurement to align with the standard clinical attachment level recorded in periodontal practice, ensuring the output is directly interpretable and relevant for clinical decision-making. This focus on replicating established clinical metrics, combined with advanced signal processing to ensure specificity, was crucial for validating this proof-of-concept tool against the photographic ground truth.

The diagnostic performance analysis was based on a limited number of independent bone loss events, which may result in wide confidence intervals for sensitivity and specificity estimates. Therefore, these values should be interpreted as preliminary and require validation in larger experimental and clinical studies.

### Comparison with other imaging modalities

A fundamental consideration in any imaging modality is the trade-off between resolution and soft-tissue penetration. While laboratory tools like optical interferometers [42,43] and optical coherence tomography (OCT) [44–46] provide superior axial resolution (sub-micron and ~4 µm, respectively), they cannot image through the gingiva, rendering them unsuitable for non-invasive clinical bone assessment. Conversely, CBCT provides 3D volumetric data but at a lower resolution (±100–200 µm) and with the significant drawback of ionizing radiation. Within this landscape, high-frequency ultrasound (HFUS) was selected for its unique ability to balance sufficient resolution (axial precision ±50 µm) with the necessary penetration to image through soft tissue to the underlying bone. This specific capability is a prerequisite for a clinically viable, non-ionizing tool for periodontal monitoring. The demonstrated 138 µm detection threshold, which is remarkably close to the system’s lateral resolution limit of 90 µm, demonstrates the ability of HFUS to detect subtle bone-level changes and fills an important diagnostic gap for early, active disease monitoring.

### Clinical implications and workflow integration

In summary, the statistical analyses in this study follow a deliberate logical progression: from characterizing the measurement noise floor through system error (Table 2), to establishing detection capability through the empirical threshold of 138 µm and sensitivity/specificity analysis (Tables 4 and 5), to validating agreement and reliability through Bland–Altman analysis (Fig. 4, S1 Table) and ICC assessment (Table 3), and finally to confirming correlation through regression analysis (Fig. 4d). This progression demonstrates that the clinical value of HFUS for periodontal monitoring rests not on absolute measurement accuracy where the wide limits of agreement (676–844 µm range) indicate that single measurements should not be considered interchangeable with photographic references but on measurement precision, where the low system error (77–113 µm) and empirical detection threshold of (138 µm) enable detection of longitudinal changes approximately six times smaller than the 870 µm threshold reported for conventional radiography. The core clinical insight is that periodontal disease management depends on detecting changes from a patient’s own baseline over time, not on a single absolute bone-level value. The HFUS framework established in this study particularly through the automated DTS-US and Auto-SVD-US workflows provides the precision, reliability, and detection sensitivity required for this task, while eliminating ionizing radiation exposure and enabling more frequent monitoring intervals. This quantitative framework shifts the role of HFUS from a purely imaging modality to a validated measurement tool for longitudinal periodontal bone loss monitoring.

This study establishes that HFUS, particularly via automated algorithms, enables reliable, radiation-free quantification of alveolar bone loss with a sub-millimeter detection capability. Its optimal clinical application is in the safe, repetitive monitoring of disease progression, facilitating a shift from reactive to proactive periodontal care. In a potential clinical workflow, ultrasound acquisition at a single periodontal site requires approximately 10–20 seconds, and operator training primarily involves probe positioning and CEJ identification. With automated processing, the workflow could be integrated into routine periodontal monitoring visits.

Importantly, the detection threshold (~138 µm) lies well below the limits of agreement, reinforcing that the system can detect meaningful changes even in the presence of variability in absolute measurements.

### Limitations

This study has several limitations. First, the experiments were performed using an *ex vivo* swine jaw model without soft tissue inflammation or physiological conditions, which may differ from in vivo imaging conditions and may not fully represent inflammatory bone resorption seen in periodontitis. Specifically, we now state that the study was conducted on multiple teeth within a single porcine mandible, with measurements performed across controlled bone-loss stages. We further clarify that, although multiple teeth, stages, and repeated measurements were included, these observations do not represent independent biological samples and should be interpreted within a repeated-measurement framework. The potential for pseudoreplication is now explicitly acknowledged. While this design enables detailed evaluation of measurement performance, it does not capture biological variation between different animals. However, this study was designed as a proof-of-concept validation of measurement performance under controlled conditions, and the measurement framework and methodology remain applicable across conditions. The specimen was selected by convenience sampling based on availability, intact anatomy, and suitability for controlled defect creation. This limits the generalizability of the findings to broader biological variation, different tooth types, different ages, and clinical periodontal conditions. No experimental randomization was performed for specimen selection or defect creation. The randomization used in this study applied only to the blinded image-evaluation process, where images were anonymized and presented in random order to reduce observer bias. Formal normality testing was not performed for all statistical comparisons due to the limited number of independent experimental units. Baseline error distributions were visually inspected and appeared approximately normally distributed. Therefore, ANOVA- and ICC-based results should be interpreted as measures of internal consistency and reliability within this controlled repeated-measurement framework rather than as population-level inference. Confidence intervals for sensitivity and specificity are inherently limited by the small number of independent bone-loss events in this proof-of-concept study and may therefore be wide and less reliable. Accordingly, sensitivity and specificity values should be interpreted as preliminary estimates of detection performance rather than definitive diagnostic accuracy metrics. No acquired datasets, images, or measurements were excluded from the analysis, and no data loss occurred. Second, alveolar bone defects were created using controlled mechanical removal rather than inflammatory bone resorption, which may result in different bone surface morphology compared to clinical bone loss patterns.

Third, the study was performed under controlled experimental conditions with careful probe positioning and alignment, which may differ from clinical scanning conditions where operator variability, saliva, soft tissue thickness, and patient movement may influence image quality and measurement accuracy.

Fourth, steel ball fiducial markers were used only in the ex vivo model to establish precise ground truth reference measurements and frame alignment. These markers are not clinically applicable; however, the automated measurement method developed in this study uses the cementoenamel junction (CEJ) as a natural anatomical reference point, which is clinically applicable and eliminates the need for fiducial markers.

Finally, although this study demonstrates measurement precision and detection capability of high-frequency ultrasound, further in vivo studies and clinical trials are required to evaluate clinical performance, reproducibility, and workflow integration.

Despite these limitations, this study establishes a quantitative framework for evaluating measurement precision, system error, and minimum detectable change for longitudinal monitoring of alveolar bone loss using high-frequency ultrasound. In addition, the number of independent bone loss events used for diagnostic performance analysis was limited, and therefore sensitivity and specificity estimates should be interpreted as preliminary.

## Conclusion

This proof-of-concept study demonstrated that high-frequency ultrasound can quantify alveolar bone loss with low system error, strong agreement with photographic measurements, and the ability to detect small incremental changes of approximately 138 µm. These results indicate that high-frequency ultrasound is particularly suitable for longitudinal monitoring of alveolar bone loss progression rather than for single absolute measurements. The automated ultrasound methods showed high reliability and measurement consistency, supporting their potential for future clinical monitoring applications. It should be noted that conventional radiography was not directly evaluated in this study; therefore, comparisons with radiographic detection limits are based on previously published literature and should be interpreted with caution. The present study demonstrates the measurement precision and detection capability of HFUS in a controlled experimental model rather than direct clinical diagnostic superiority. High-frequency ultrasound should therefore be considered a tool for longitudinal monitoring of periodontal bone loss progression rather than a replacement for radiographic assessment of absolute bone levels.

## Supporting information

S1 Table Comprehensive Bland–Altman agreement statistics. (PDF)

S1 Code Alveolar Bone Loss Analysis MATLAB GUI Code. (PDF)

S1 Text Post hoc statistical power analysis. (PDF)
