# Supplementary material for: Sub-millimeter quantification of alveolar bone loss using automated 40 MHz high-frequency ultrasound: A proof-of-concept ex vivo validation study
Source: PLoS One. 2026 Jun 8;21(6):e0349815. doi: 10.1371/journal.pone.0349815 (PMC13245748; doi:10.1371/journal.pone.0349815)
Supplement: S1 Table — (PDF) [file pone.0349815.s001.pdf]

S1 Table. Comprehensive Bland-Altman agreement statistics

Comparison	Bias (µm) or %	Lower LOA (µm)	Upper LOA (µm)	Comparison	Bias (µm) or %	Lower LOA (µm)	Upper LOA (µm)
**Primary Method Comparisons (Ultrasound vs Camera)**				**Primary Ultrasound Method Comparisons**			
**Manual-BO-US vs Manual-BO-CA (All examiners)**	30/15%	-307	369	**DTS-US vs Manual-BO-US**	138/69%	-391	667
**DTS-US vs Manual-BO-CA**	86/43%	-336	508	**US-Auto vs Manual-BO-US**	34/17%	-430	497
**Auto-SVD-US vs Manual-BO-CA**	-18 / 9%	-418	381	**Auto-SVD-US vs DTS-US**	105/52.5%	-225	435
**Manual-BO-US vs Auto-CA**	67/33.5%	-360	495	**Primary Camera Method Comparisons**			
**DTS-US vs Auto-CA**	71/35.5%	-288	430	**Manual-BO-CA vs Auto-CA**	15/7.5%	-330	360
**Auto-SVD-US vs Auto-CA**	-33/16.5%	-379	312				
**Inter-Observer CA (Manual)**				**Ultrasound vs Camera (Manual blind examiners)**			
**Ex1 vs Ex2 Manual-BO-CA**	58/29%	-430	546	**Ex1 (Manual-BO-US Vs Manual-BO-CA)**	14/7%	-440	470
**Ex1 vs Ex3 Manual-BO-CA**	-69/34.5%	-875	736	**Ex2 (Manual-BO-US Vs Manual-BO-CA)**	-60/30%	-530	410
**Ex2 vs Ex3 Manual-BO-CA**	-127/63.5%	-943	689	**Ex3 (Manual-BO-US Vs Manual-BO-CA)**	107/53.5%	-563	778
**Inter-Observer US (Manual)**							
**Ex1 vs Ex2 Manual-BO-US**	17/8.5%	-410	443				
**Ex1 vs Ex3 Manual-BO-US**	-24/12%	-650	602				
**Ex2 vs Ex3 Manual-BO-US**	-40/20%	-531	451				
