# Supplementary material for: Sub-millimeter quantification of alveolar bone loss using automated 40 MHz high-frequency ultrasound: A proof-of-concept ex vivo validation study
Source: PLoS One. 2026 Jun 8;21(6):e0349815. doi: 10.1371/journal.pone.0349815 (PMC13245748; doi:10.1371/journal.pone.0349815)
Supplement: S1 Code — (PDF) [file pone.0349815.s002.pdf]

S2 Code. Alveolar Bone Loss Analysis MATLAB GUI Code:

The MATLAB code used for automated measurements is provided as Supporting Information (S1 Code).

Gethub link (for public)

https://github.com/DrTamer/Alveolar-Bone-Loss-GUI

This is a supplementary material of a manuscript for a homemade MATLAB - GUI software for Quantification of Alveolar Bone Loss Using High-Frequency Ultrasound (HFUS).
Access is open to the public after publication.

Please email: Tamer Abdelrehim for any inquiry regarding the GUI MATLAB code (AlveolarBoneLoss_Analysis) at tamerniskriss@gmail.com
