# Supplementary material for: Sub-millimeter quantification of alveolar bone loss using automated 40 MHz high-frequency ultrasound: A proof-of-concept ex vivo validation study
Source: PLoS One. 2026 Jun 8;21(6):e0349815. doi: 10.1371/journal.pone.0349815 (PMC13245748; doi:10.1371/journal.pone.0349815)
Supplement: S1 Text — (PDF) [file pone.0349815.s003.pdf]

S3 Text. Post hoc statistical power analysis

A post hoc statistical power analysis was performed to assess whether the experimental design provided sufficient statistical sensitivity to detect clinically relevant changes in alveolar bone levels. Because this study was designed as a methodological proof-of-concept and validation study rather than a conventional hypothesis-testing trial, the power analysis was conducted after completion of the experiment using the observed effect size and measurement variability.

1. Define the detectable effect

The empirically determined minimum detectable bone loss increment reported in this study was:

$$\Delta = 138\ \mu m$$

This value represents the smallest bone loss increment that was consistently detected above the method-specific system error threshold.

2. Define the measurement variability

The reported system error across ultrasound-based methods (Manual-BO-US, DTS-US, US-Auto, and Auto-SVD-US) ranged from:

$$77 \mu m \leq \sigma \leq 113\ \mu m$$

To obtain a representative variability value for post hoc effect-size estimation, the midpoint of this range was used:

$$\sigma_{\text{avg}} = \frac{77 + 113}{2} = 95\ \mu m$$

This average standard deviation provides a practical summary of the measurement noise floor across methods.

3. Calculate the standardized effect size

The standardized effect size was estimated using Cohen's d, defined as:

$$d = \frac{\Delta}{\sigma}$$

Substituting the observed values:

$$d = \frac{138}{95} = 1.45$$

Thus, the estimated effect size was:

$$d \approx 1.45$$

According to conventional interpretation, an effect size of $d = 1.45$ is considered large.

For completeness, the effect size range based on the minimum and maximum reported system error values can also be calculated:

Using the largest system error:

$$d_{\text{min}} = \frac{138}{113} = 1.22$$

Using the smallest system error:

$$d_{\text{max}} = \frac{138}{77} = 1.79$$

Therefore, the estimated effect size range was:

$$1.22 \leq d \leq 1.79$$

This confirms that the observed effect remained large across the full reported range of measurement variability.

4. Define the effective sample size

The study used repeated measurements at each stage for each modality. As described in method, each stage produced:

$$4 \text{ frames} \times 3 \text{ repetitions} \times 3 \text{ examiners} = 36 \text{ measurements}$$

Thus, the per-stage measurement count was:

$$n = 36$$

This value was used as a conservative effective sample size for the post hoc power estimate. It should be noted that the full study included multiple teeth and multiple bone loss stages, but to avoid overstating power due to repeated-measures structure, the per-stage value of $n = 36$ provides a cautious estimate.

5. Specify the statistical framework

Power was estimated under a one-sample / paired-difference detection framework using:

- significance level:

$$\alpha = 0.05$$

- two-sided testing assumption
- standardized effect size:

$$d = 1.45$$

- effective sample size:

$$n = 36$$

The non-centrality parameter for the t-based power approximation is:

$$\delta = d\sqrt{n}$$

Substituting the observed values:

$$\delta = 1.45\sqrt{36} = 1.45 \times 6 = 8.70$$

This is a very large non-centrality parameter, indicating strong separation between the detectable change and the measurement noise distribution.

6. Estimate statistical power

Using the above parameters, the statistical power is well above the conventional 80% threshold. With:

$$d = 1.45, n = 36, \alpha = 0.05$$

the estimated power exceeds 0.99 under a standard t-test approximation:

$$\text{Power} > 0.99$$

Even under the most conservative effect-size estimate based on the highest system error:

$$d = 1.22, n = 36, \alpha = 0.05$$

For the conservative effect-size estimate based on the highest observed system error, the standardized effect size was:

$d = 138 / 113 = 1.22$

Using the same effective sample size and significance level:

$d = 1.22$, $n = 36$, $\alpha = 0.05$

the non-centrality parameter for the t-test approximation was calculated as:

$\delta = d \times \sqrt{n} = 1.22 \times \sqrt{36} = 1.22 \times 6 = 7.32$

For a two-sided test with $\alpha = 0.05$ and degrees of freedom df = 35, the critical t value is approximately 2.03. Because the non-centrality parameter (7.32) is substantially larger than the critical value, the probability of detecting the observed effect is very high. Under this conservative scenario, the estimated statistical power remained above 0.95.

Thus, both the average-case estimate ($d = 1.45$) and the conservative estimate ($d = 1.22$) indicate that the study had sufficient statistical power to detect clinically relevant bone loss changes. These calculations support the robustness of the experimental design despite the methodological validation nature of the study and the use of a single ex vivo specimen.

the estimated power remains very high and still exceeds 0.95.

Therefore, across the plausible range of observed measurement variability, the study had sufficient statistical power to detect clinically relevant bone loss changes.

7. Interpretation

This analysis indicates that the study was adequately powered for its primary methodological objective: detection of small longitudinal changes in bone level relative to measurement variability. The high power is driven by two factors:

1. the large observed effect size, because the empirical detection threshold (138 µm) exceeded the system noise floor (77–113 µm), and
2. the repeated-measurement design, which provided a sufficiently large number of observations per stage.

Accordingly, although no a priori sample size calculation was performed, the post hoc analysis supports the statistical robustness of the validation framework and indicates that the study had more than adequate power to detect clinically relevant incremental bone changes under the reported experimental conditions.
