# Supplementary material for: Sub-millimeter quantification of alveolar bone loss using automated 40 MHz high-frequency ultrasound: A proof-of-concept ex vivo validation study
Source: PLoS One. 2026 Jun 8;21(6):e0349815. doi: 10.1371/journal.pone.0349815 (PMC13245748; doi:10.1371/journal.pone.0349815)
Supplement: Attachment — Submitted filename: Response to Reviewers_Final.docx [file pone.0349815.s005.docx]

**Response to Reviewers**

RESEARCH ARTICLE

**Sub-Millimeter Quantification of Alveolar Bone Loss Using Automated 40 MHz High-Frequency Ultrasound: A Proof-of-Concept Ex Vivo Validation Study**

Tamer Abdelrehim^1^, Baiyan Qi^1^, Bryant Tran^2^, Mirali Pandya^3^, Jesse V. Jokerst^*1,3,4^, Casey Chen^*2^

**1** Aiiso Yufeng Li Family Department of Chemical and NanoEngineering, University of California San Diego, La Jolla, California, United States of America, **2** Herman Ostrow School of Dentistry, University of Southern California, Los Angeles, California, United States of America, **3** Materials Science and Engineering Program, University of California San Diego, La Jolla, California, United States of America, **4** Department of Radiology, University of California San Diego, La Jolla, California, United States of America

* [ccchen@usc.edu](mailto:ccchen@usc.edu), [jjokerst@ucsd.edu](mailto:jjokerst@ucsd.edu)

We would like to thank the academic editor and the reviewers for their careful evaluation of our manuscript and for their constructive and insightful comments. We have revised the manuscript thoroughly according to the reviewers’ suggestions. We believe that these revisions have significantly improved the clarity, structure, and scientific presentation of the manuscript.

Below we provide a detailed, point-by-point response to each reviewer comment. All changes have been incorporated into the revised manuscript and are indicated in the tracked changes version.

Reviewer comment: **with bold black**.

Author Response: with regular black.

New text edited at the manuscript: with regular blue

**Reviewer #1:**

**Title
- "Quantification of Alveolar Bone Loss Using High-Frequency Ultrasonography for Precision Periodontal Monitoring" - please stick to Plos One's Guidelines for Authors at** [**https://journals.plos.org/plosone/s/submission-guidelines**](https://urldefense.com/v3/__https:/journals.plos.org/plosone/s/submission-guidelines__;!!Mih3wA!FLE0aotSpd4UP2c79Uc7740xERE9yvlh8OapMAj8aJKHbvuYeNbbH-bgOuOzSrUOlK0xYBcr-siNza_DsA$)**. No capital letters.
- Please provide type of study.**

Response:
We thank the reviewer for this helpful comment. The manuscript title has been revised to follow PLOS ONE style guidelines using sentence case formatting rather than title case.

We have revised the author affiliations to comply with PLOS ONE formatting requirements. Street addresses and postal codes were removed, and affiliations were reformatted to include department, institution, city, state, and country only. Corresponding author information was also reformatted according to journal guidelines. We have carefully revised the manuscript formatting to follow PLOS ONE guidelines. Section headings were reformatted using sentence case, section numbering was removed, figure citation format was corrected, and tables were placed within the main manuscript as required. The funding statement and supporting information sections were also reorganized according to journal formatting requirements.

Change in manuscript:

The title capitalization has been corrected on the title page.
The title page and affiliations section have been updated accordingly.
Formatting corrections were applied throughout the manuscript.

RESEARCH ARTICLE

**Sub-millimeter quantification of alveolar bone loss using automated 40 MHz high-frequency ultrasound: a proof-of-concept *ex vivo* validation study**

Tamer Abdelrehim^1^, Baiyan Qi^1^, Bryant Tran^2^, Mirali Pandya^3^, Jesse V. Jokerst^*1,3^, Casey Chen^*2^

**1** Aiiso Yufeng Li Family Department of Chemical and Nanoengineering, University of California San Diego, La Jolla, California, United States of America, **2** Herman Ostrow School of Dentistry, University of Southern California, Los Angeles, California, United States of America, **3** Materials Science and Engineering Program, University of California San Diego, La Jolla, California, United States of America

* [ccchen@usc.edu](mailto:ccchen@usc.edu), [jjokerst@ucsd.edu](mailto:jjokerst@ucsd.edu)

**Abstract
- With your current 174 words, you do not provide sufficient information. Please stick to the 300-word count allowed for with this section.
- Provide sound methodology, and stick to exact results, along with exact P values-
- With your Conclusions, please stick exclusively to your aims. Do not simply repeat your results here. Do not speculate. Do not provide well-accepted (but meaningless) phrases. Instead, provide a reasonable and generalizable extension of your outcome.**

Response:
We thank the reviewer for this important suggestion. The abstract has been substantially revised and expanded to provide additional methodological details, including the experimental model, statistical analysis methods, and measurement evaluation approach. Quantitative results and statistical findings, including system error, minimum detectable change, agreement analysis, and statistical comparisons, have been added to the abstract. The conclusion has been rewritten to focus specifically on the study aims and findings, avoiding speculative statements and emphasizing the generalizable outcome of the study.

The keywords have been revised to better reflect the focus and contribution of the study, including high-frequency ultrasound, alveolar bone loss, periodontal monitoring, and longitudinal monitoring concepts. The keywords were also reformatted according to journal guidelines.

**Change in manuscript:**
The abstract has been completely revised and expanded to include methodology, quantitative results, statistical analysis, and a revised conclusion aligned with the study objectives.

The keywords section has been updated accordingly.

### **Abstract**

This proof-of-concept study evaluated the performance of high-frequency ultrasound (HFUS, 40 MHz) for monitoring changes in alveolar bone loss and determining the detection limits and measurement precision of ultrasound at this center frequency. The study quantified the system error, minimum detectable change, and agreement with a photographic ground truth measurement. An *ex vivo* model was used in which controlled bone loss increments were created and measured using both manual and automated HFUS methods. System error was calculated using repeated baseline measurements, and detection capability was assessed using sensitivity analysis across incremental bone loss values. Agreement between methods was evaluated using Bland–Altman analysis and intraclass correlation coefficients (ICC). The system error ranged from 77 µm for automated image-registration measurements to 113 µm for manual measurements. The minimum detectable bone loss increment was experimentally determined to be approximately 138 µm. Detection sensitivity ranged from 79% to 100%, depending on the measurement method and increment size. Automated ultrasound measurements showed strong agreement for longitudinal change tracking, with a best-case bias of −18 µm corresponding to less than 10% error relative to a typical 200 µm bone change increment and excellent reliability (ICC = 0.89–0.99). No significant systematic bias was observed compared with photographic measurements (*P* > 0.05), indicating good agreement between methods. HFUS thus demonstrated detects small longitudinal changes in alveolar bone loss and can track bone loss progression over time with high measurement precision. The main contribution of this study is demonstrating that HFUS can be used as a radiation-free method for monitoring longitudinal changes in alveolar bone loss rather than relying solely on absolute measurement accuracy.

**Keywords**: high-frequency ultrasound; alveolar bone loss; periodontal monitoring; precision medicine; non-ionizing radiation; digital dentistry

**Intro
- Your "Clinical Relevance/Background:" does not stick to Journal style. Please consult some recently published Plos One papers, and revise carefully.
- Same with "Added Value of This Study:" and "Clinical Implications:".
- Same with your references. "(...) change for detection ¹." must read "(...) change for detection [1]."
- Same with "1. INTRODUCTION". Again, stick to the Journal's guidelines, and consult some recently published Plos One papers. Revise carefully, but thoroughly.
- Please provide a sound null hypothesis.**

Response – Introduction

We thank the reviewer for the helpful suggestions regarding the introduction structure and formatting. The Introduction section has been revised to follow PLOS ONE formatting guidelines. The sections previously labeled “Clinical Relevance,” “Added Value of This Study,” and “Clinical Implications” have been removed, and the introduction has been reorganized into a continuous narrative format. Reference formatting has been corrected to the journal’s numbered citation style. In addition, a clear null hypothesis has been added at the end of the Introduction section as requested.

**Meths
- Again, please compare "2. Materials and Methods", and revise for uniform Journal style.
- With ALL materials (including chemicals) and methodologies (including statistical software), please use general/non-proprietary names with your text, followed by (brand name; manufacturer, city, ST[ate - abbreviated, if US], country) in parentheses. Stick to semicolon. Ensure reproducibility, and revise thoroughly.
- "h (height) x w (width)" must read "h (height) × w (width)". Please use symbol × instead of letter x. Revise thoroughly throughout your text.**

Response – Method

We thank the reviewer for this important suggestion. The Materials and methods section have been revised throughout to use general or non-proprietary names followed by product and manufacturer details in parentheses in the format requested by the journal, including brand name; manufacturer; city, state, and country where applicable. We also standardized software and statistical tool descriptions using the same format to improve reproducibility.

**Results
- With your P values, please stick to Journal style.**

Response – Results

We thank the reviewer for this helpful comment. All P values throughout the manuscript have been revised to follow the journal style. Specifically, P values are now reported using italicized uppercase P with appropriate spacing (e.g., P = 0.03, P < 0.001), and the term “p-value” has been replaced with “P value” where appropriate. These changes have been applied consistently throughout the Abstract, Results section, tables, and figure captions.

All P values throughout the manuscript have been revised to follow the journal style. P values are now reported using uppercase italic *P* with appropriate spacing (e.g., *P* = 0.03, *P* < 0.001), and the term “p-value” has been replaced with “*P* value” where appropriate.

**Disc
- What about H0?
- "Our systematic evaluation confirms that HFUS is a precise and reliable alternative." Alternative to what?
- What about the strengths of your study?
- What about the limitations?
- Please provide sound responses to your teaching objectives.**

**Response – Discussion**

**Discussion – Null hypothesis**

A clear null hypothesis statement has been added to the Discussion section, and the results are now explicitly interpreted in relation to the null hypothesis.

**Introduction**

….

The null hypothesis of this study was that high-frequency ultrasound measurements would not differ significantly from photographic reference measurements for quantifying alveolar bone loss and would not be capable of detecting small incremental bone loss changes.

**Null hypothesis and study goal**

The null hypothesis of this study was that high-frequency ultrasound measurements would not differ significantly from photographic reference measurements and would not be capable of detecting small incremental changes in alveolar bone loss. Based on the results demonstrating low system error, strong agreement with photographic measurements, and reliable detection of small bone loss increments, the null hypothesis was rejected.

**Discussion – Alternative to what**

The sentence stating that HFUS is an alternative has been revised to clarify that HFUS is an alternative to conventional radiographic methods for monitoring alveolar bone loss. Then we are reformatted the sentences because in this high-frequency ultrasound is not intended to replace radiographic methods for absolute bone level measurement, but rather to provide a high-precision, radiation-free tool for detecting small longitudinal changes in alveolar bone loss.

**Discussion – Strengths and limitations**

New paragraphs describing the strengths and limitations of the study have been added to the Discussion section, including the controlled ex vivo model, precise ground truth measurements, and limitations related to the ex vivo experimental design and operator variability.

**Disc

- Please provide sound responses to your teaching objectives.**

**Discussion – Teaching objectives**

We thank the reviewer for this important comment. The Discussion section has been revised to provide clear and structured responses to the study objectives through a question-and-answer format (Q1–Q3).

For each objective, we explicitly linked the corresponding results to their interpretation and clinical implications, including measurement precision, agreement with reference methods, and detection capability. These revisions ensure that each study objective is addressed with clear, evidence-based conclusions and directly connected to the clinical relevance of high-frequency ultrasound for longitudinal periodontal monitoring.

**Concl
- Again, with your Conclusions, please stick exclusively to your aims. Do not simply repeat your results here. Do not speculate. Do not provide well-accepted (but meaningless) phrases. Instead, provide a reasonable and generalizable extension of your outcome.**

**Response – Conclusion**

The Conclusion section has been rewritten to focus strictly on the study aims and generalizable outcomes. Repetitive statements and speculative language were removed, and the conclusion now emphasizes the role of high-frequency ultrasound for longitudinal monitoring rather than absolute measurement.

**Refs
- Revise for sound and uniform Journal style. Again, stick to Authors' Guidelines.**

**Response – References**

The reference list has been revised to ensure uniform formatting according to the journal guidelines.

**In total, this draft would seem interesting, but would not seem ready to proceed. Sticking to the Journal's guidelines would seem mandatory. Please remember that Authors (but neither Reviewers nor Editors or Typesetters)) are responsible for sound drafts and note that one review is considered standard.**

Overall, the manuscript has been thoroughly revised to comply with the journal style and to improve clarity, structure, and scientific interpretation.

We thank the reviewer #1 for the detailed formatting and abstract suggestions. These revisions significantly improved the clarity, structure, and presentation of the manuscript and ensured full compliance with PLOS ONE formatting guidelines.

**Reviewer #2:**

**This study is fundamental research toward quantifying alveolar bone resorption using high-frequency ultrasound. As stated in the conclusion, it holds the potential to revolutionize future periodontal disease diagnosis. Specifically, conventional diagnosis using two-dimensional images from X-rays primarily detects alveolar bone resorption centered on the mesio-distal regions of teeth where the root and alveolar bone do not overlap. This study demonstrates results enabling effective detection of alveolar bone resorption in the buccal and lingual central regions, areas where early bone resorption is difficult to detect. However, considering actual clinical settings, challenges remain for the practical implementation of alveolar bone resorption detection using this system.**

Response to Reviewer #2

We sincerely thank the reviewer for the thoughtful and constructive comments. We appreciate the reviewer’s recognition that this work represents fundamental research toward quantifying alveolar bone loss using high-frequency ultrasound and its potential future clinical impact. We have carefully revised the manuscript to address all comments, particularly regarding marker placement, anatomical complexity, model limitations, and clinical applicability. Our detailed responses are provided below.

**1. Reference markers were fixed into drilled holes using cyanoacrylate adhesive (Gorilla Glue/Super Glue) and positioned as reference markers 3-4mm above the initial alveolar bone level on teeth M3, M2, and PM4. However, on M3, M2, and PM4, where exactly on the buccal side (mesial, central, or distal) were these markers placed? Furthermore, this reference 20 does not appear to be an appropriate citation.**

Response:

The reference markers were placed on the buccal surface at the central region of each tooth (M3, M2, and PM4), approximately 3–4 mm above the initial alveolar bone level and within the lateral FOV for ultrasound frame. The markers were positioned centrally to avoid mesial and distal anatomical variations and to provide a consistent vertical reference point for manual measurements.

We would like to clarify that the drilled holes and stainless-steel ball markers fixed using cyanoacrylate adhesive were used only in the ex vivo pig jaw model to establish fixed reference points for manual and photographic measurements. More important, under ultrasound scan, these reference steel balls are a clear ultrasound ring down marker that ensure the comparison of the same frame between different bone loss stages at different times. This method is not intended for clinical application.

For clinical application, our automatic ultrasound measurement method does not require drilled markers. Instead, the cementoenamel junction (CEJ) is used as a natural anatomical reference point, which can be reliably identified in clinical ultrasound images.

Regarding the use of old Reference [20] Now reference number changed [14], we agree that it does not describe the exact marker placement technique used in our study. However, it was cited because it demonstrates the use of a fixed reference structure (implant) to evaluate bone level changes using imaging techniques such as CT and radiography. The concept is like our study, where fixed reference points are required to measure bone level changes accurately. In our ex vivo model, stainless steel ball markers were used as fixed reference points, conceptually like using an implant as a reference structure in imaging-based bone level assessment. We now using another more suitable reference [Qi, B., Fu, L., Abdelrehim, T. et al. Ultrasound identification of the cementoenamel junction and clinical correlation through ex vivo analysis. Sci Rep 14, 27821 (2024). <https://doi.org/10.1038/s41598-024-79081-z>] of our previous study that using the similar steel balls to measure the position of CEJ using HFUS. We have clarified this explanation in the revised manuscript.

An intrasulcular incision was made from the distobuccal area of the third mandibular molar (M3) to the mesiobuccal area of the fourth mandibular premolar (PM4) using a #15 surgical blade (Henry Schein Inc., Melville, NY, USA). A full-thickness mucoperiosteal flap was elevated with a periosteal elevator (#9 Molt double-ended periosteal elevator; Henry Schein Inc., Melville, NY, USA) to expose the underlying alveolar bone. Small holes were drilled on the buccal surface at the central region of each tooth (M3, M2, and PM4), approximately 3–4 mm above the initial alveolar bone level and within the lateral FOV for ultrasound frame. Stainless steel ball markers (1 mm diameter) were fixed in the drilled holes using cyanoacrylate adhesive (Gorilla Super Glue; Gorilla Glue Company, Cincinnati, OH, USA) to serve as stable fiducial reference markers for calibration, motion tracking, and bone level measurements [14]. Two steel balls were positioned above and below the initial alveolar bone level to provide a two-point reference for pixel-to-micron calibration. Under ultrasound scan, these reference steel balls are a clear ultrasound ring marker that ensure the comparison of the same frame between different bone loss stages at different times. The mandibles were then secured on a C-clamp holder with articulating platforms (Thorlabs, Inc., Newton, NJ, USA) [26]. The distance between steel balls on each tooth was measured using a digital caliper with 0.01-mm resolution (Neiko Tools USA, CA, USA). The markers were placed in the central buccal region to minimize mesial and distal anatomical variations and to ensure consistent vertical reference measurements. This method was used only for the *ex vivo* experimental model and is not intended for clinical application. For clinical ultrasound measurements, the cementoenamel junction (CEJ) was used as the anatomical reference point. The use of fixed reference markers in this study is conceptually similar to our previous studies [14] in which use of fixed structures, such as the steel balls, were used as reference points for measuring the position of CEJ using imaging HFUS.

**2. Related to the above point, when the current analysis site has a relatively simple morphology, it raises the question of whether high-frequency ultrasound can detect bone resorption within complex three-dimensional periodontal structures, such as adjacent interdental spaces and furcations, which are common sites for periodontitis.**

Response:

Thank you for this important comment. We agree that the current ex vivo model used relatively simple bone morphology, and complex periodontal structures such as interdental spaces and furcation areas present additional challenges for ultrasound imaging.

However, previous studies, including our laboratory work using high-frequency ultrasound for periodontal imaging, have demonstrated that ultrasound can identify key periodontal anatomical landmarks such as the cementoenamel junction (CEJ), gingival margin, and alveolar bone crest in human subjects and extracted swine jaws. These studies show that ultrasound imaging can resolve periodontal structures and measuring alveolar bone levels in clinically relevant conditions, not only in simple geometries.

The ultrasound measurement principle is based on detecting strong acoustic reflections at the soft tissue–bone interface, which is independent of whether the bone surface is flat or part of a complex three-dimensional structure. However, complex anatomical regions such as interdental spaces and furcations may introduce challenges related to probe positioning, beam angle, acoustic shadowing, and physical access limitations of the ultrasound transducer.

Therefore, the purpose of this study was to first validate the quantitative bone level measurement method under controlled experimental conditions. Future studies will focus on evaluating the performance of this method in more complex periodontal defects, including interdental and furcation regions, as well as in in vivo clinical conditions and design of new transducers for these research targets like oral brush transducers. This limitation has been added to the discussion section of the revised manuscript.

**Limitations**

This study has several limitations. First, the experiments were performed using an ex vivo swine jaw model without soft tissue inflammation or physiological conditions, which may differ from in vivo imaging conditions and may not fully represent inflammatory bone resorption seen in periodontitis. Second, alveolar bone defects were created using controlled mechanical removal rather than inflammatory bone resorption, which may result in different bone surface morphology compared to clinical bone loss patterns.

Third, the study was performed under controlled experimental conditions with careful probe positioning and alignment, which may differ from clinical scanning conditions where operator variability, saliva, soft tissue thickness, and patient movement may influence image quality and measurement accuracy.

Fourth, steel ball fiducial markers were used only in the *ex vivo* model to establish precise ground truth reference measurements and frame alignment. These markers are not clinically applicable; however, the automated measurement method developed in this study uses the cementoenamel junction (CEJ) as a natural anatomical reference point, which is clinically applicable and eliminates the need for fiducial markers.

Finally, although this study demonstrates measurement precision and detection capability of high-frequency ultrasound, further in vivo studies and clinical trials are required to evaluate clinical performance, reproducibility, and workflow integration.

Despite these limitations, this study establishes a quantitative framework for evaluating measurement precision, system error, and minimum detectable change for longitudinal monitoring of alveolar bone loss using high-frequency ultrasound. In addition, the number of independent bone loss events used for diagnostic performance analysis was limited, and therefore sensitivity and specificity estimates should be interpreted as preliminary.

**3. Inflammatory bone resorption due to periodontitis dissolves the superficial cortical bone and causes complex changes in the structure of the cancellous bone. The alveolar bone resorption induced in this study resulted from artificial bone removal using a chisel. It is possible that this method may not adequately reflect the marginal bone loss caused by alveolar bone resorption associated with actual soft tissue inflammation, particularly at the bone margin. For instance, an analysis using experimental periodontitis in animal models would provide a more realistic analytical model reflecting actual alveolar bone resorption. Therefore, it would be ideal if the validity of the findings could be assessed in such a model.**

**Response**

Thank you for this insightful comment. We agree that inflammatory bone resorption associated with periodontitis results in more complex structural changes in both cortical and cancellous bone compared to mechanically created defects. In this study, bone defects were created using controlled mechanical removal to produce known and measurable bone level changes. This approach allowed us to validate the accuracy and repeatability of the ultrasound measurement method under controlled conditions.

The primary objective of this study was to validate a quantitative measurement method for detecting alveolar bone level changes using high-frequency ultrasound, rather than to replicate the biological process of periodontitis-related bone resorption. Using controlled mechanical defects allowed us to establish a ground truth for measurement accuracy and method validation.

We agree that experimental periodontitis models in animals would provide a more realistic model of inflammatory bone resorption and would be an important next step to evaluate the clinical applicability of this method. Future studies will investigate this method in animal models with experimentally induced periodontitis and in clinical in vivo conditions. This limitation has been added to the discussion section of the revised manuscript.

The main aim of this study was to develop a reliable ultrasound imaging and analysis method for alveolar bone loss that can be used to monitor longitudinal changes and be applied in clinical human studies without exposure to ionizing radiation. High-frequency ultrasound is not intended to replace radiographic methods for absolute bone level measurement, but rather to provide a high-precision, radiation-free tool for detecting small longitudinal changes in alveolar bone loss.

**4. The limitations of this study are not mentioned in the final section of the discussion. For this model to be effectively applied in clinical practice, there must be outstanding issues that must be overcome.**

**Response:**

Thank you for this important comment. We agree that the limitations of the study should be clearly discussed, especially regarding clinical translation of the proposed method. We have added a limitations paragraph in the Discussion section of the revised manuscript.

The main limitations include the use of an ex vivo pig jaw model, mechanically created bone defects rather than inflammatory bone resorption, relatively simple bone morphology compared to complex periodontal structures, and the use of reference markers only applicable to the ex vivo setup. We also note that probe positioning and access may be more challenging in posterior teeth and furcation areas. Future studies will evaluate this method in animal models with experimental periodontitis and in clinical in vivo conditions. Future studies will include the development of new designs for oral transducers, specifically optimized to provide clearer imaging in complex anatomical regions.

**Limitations**

This study has several limitations. First, the experiments were performed using an ex vivo swine jaw model without soft tissue inflammation or physiological conditions, which may differ from in vivo imaging conditions and may not fully represent inflammatory bone resorption seen in periodontitis. Second, alveolar bone defects were created using controlled mechanical removal rather than inflammatory bone resorption, which may result in different bone surface morphology compared to clinical bone loss patterns.

Third, the study was performed under controlled experimental conditions with careful probe positioning and alignment, which may differ from clinical scanning conditions where operator variability, saliva, soft tissue thickness, and patient movement may influence image quality and measurement accuracy.

Fourth, steel ball fiducial markers were used only in the *ex vivo* model to establish precise ground truth reference measurements and frame alignment. These markers are not clinically applicable; however, the automated measurement method developed in this study uses the cementoenamel junction (CEJ) as a natural anatomical reference point, which is clinically applicable and eliminates the need for fiducial markers.

Finally, although this study demonstrates measurement precision and detection capability of high-frequency ultrasound, further in vivo studies and clinical trials are required to evaluate clinical performance, reproducibility, and workflow integration.

Despite these limitations, this study establishes a quantitative framework for evaluating measurement precision, system error, and minimum detectable change for longitudinal monitoring of alveolar bone loss using high-frequency ultrasound. In addition, the number of independent bone loss events used for diagnostic performance analysis was limited, and therefore sensitivity and specificity estimates should be interpreted as preliminary.

We appreciate the reviewer #2 ’s constructive comments, which helped improve the clarity, discussion, and clinical context of this study.

**Reviewer #3:**

**Reviewer #3: Dear authors,
I evaluated the article titled “Quantification of Alveolar Bone Loss Using High-Frequency Ultrasonography for Precision Periodontal Monitoring.” The goal was "to establish its system error, detection capability, and agreement with a photographic ground truth."
This is an interesting study! Therefore, some concerns were raised.

A concern was raised: is there necessity/justification for this proof-of-concept study? The justification presented by the authors was robust. They have successfully addressed the "Goldilocks" zone of periodontal imaging: (1) resolution: it was demonstrated a detection threshold (~138 μm) that is nearly 6x more sensitive than traditional radiography (~870 μm); (2) safety: it removed the ionizing radiation barrier, allowing for the "repetitive monitoring" that is currently impossible with CBCT; and (3) automation: by using SVD-filtering and automated CEJ-tracking, you address the primary criticism of ultrasound: operator subjectivity.**

Response to Reviewer #3

We thank the reviewer for the thorough evaluation and very constructive suggestions. We appreciate the reviewer’s positive comments regarding the novelty, detection sensitivity, safety advantages, and automation of the proposed high-frequency ultrasound method. We have carefully revised the manuscript according to the reviewer’s suggestions to improve clarity, structure, and clinical positioning of this proof-of-concept study.

**Revised Article - per section
1. Title: I suggest a modification… recommended: Sub-Millimeter Quantification of Alveolar Bone Loss using Automated 40 MHz High-Frequency Ultrasound: A Proof-of-Concept Study. Please check it**

**Reviewer #3 – Title**

**Reviewer suggestion:**
Change title to include proof-of-concept and sub-millimeter.

**Response**

We thank the reviewer for the suggestion regarding the title. We agree that the suggested title better reflects the proof-of-concept nature of the study and the sub-millimeter detection capability. The title has been revised accordingly.

Suggested final title as we changed:

Sub-Millimeter Quantification of Alveolar Bone Loss Using Automated 40 MHz High-Frequency Ultrasound: A Proof-of-Concept Ex Vivo Validation Study

**2. Abstract: it was condensed and with high-impact**

**Response:** Thanks, Please see the revised abstract.

### **Abstract**

This proof-of-concept study evaluated the performance of high-frequency ultrasound (HFUS, 40 MHz) for monitoring changes in alveolar bone loss and determining its detection capability and measurement precision. The study aimed to quantify system error, minimum detectable change, and agreement with a photographic ground truth measurement. An *ex vivo* model was used in which controlled bone loss increments were created and measured using both manual and automated HFUS methods. Measurements were compared to calibrated photographic measurements as the reference standard. System error was calculated using repeated baseline measurements, and detection capability was assessed using sensitivity analysis across incremental bone loss values. Agreement between methods was evaluated using Bland–Altman analysis and intraclass correlation coefficients (ICC). The system error ranged from 77 µm for automated image-registration measurements to 113 µm for manual measurements. The minimum detectable bone loss increment was experimentally determined to be approximately 138 µm. Detection sensitivity ranged from 79% to 100%, depending on the measurement method and increment size. Automated ultrasound measurements showed strong agreement for longitudinal change tracking, with a best-case bias of −18 µm corresponds to less than 10% error relative to a typical 200 µm bone change increment and excellent reliability (ICC = 0.89–0.99). No significant systematic bias was observed compared with photographic measurements (*P* > 0.05), indicating good agreement between methods. High-frequency ultrasound demonstrated the ability to detect small longitudinal changes in alveolar bone loss and to track bone loss progression over time with high measurement precision. The main contribution of this study is demonstrating that HFUS can be used as a radiation-free method for monitoring longitudinal changes in alveolar bone loss rather than relying solely on absolute measurement accuracy.

**3. Keywords: I suggest to include at least one of the following: "Precision Medicine," "Non-ionizing Radiation," or "Digital Dentistry"**

**Response – Keywords**

**Suggestion:** Add Precision Medicine / Non-ionizing Radiation / Digital Dentistry.

We thank the reviewer for this suggestion. We have updated the keywords to include additional relevant terms such as “Precision Medicine,” “Non-ionizing Radiation,” and “Digital Dentistry” to better reflect the scope and clinical relevance of the study.

**4. Introduction:
- Ensure you emphasize that periodontitis is a dynamic disease, but our current tools (radiography) are static and insensitive.
- "The 'diagnostic window' for early intervention is frequently missed because conventional radiographs require a 30-50% mineral loss before a defect becomes visible. This study introduces a 40 MHz HFUS workflow that bridges this gap by offering a theoretical axial resolution of 40 μm."

- “Periodontitis is one of the two leading diseases in dentistry, along with dental caries 2,3 resulting in pain, tooth loss 4, diminished quality of life5, and possible systemic effects such as Type II diabetes 6 and cardiovascular disease7. In patients suffering from periodontitis, bacteria-induced inflammatory responses destroy soft and hard tissues supporting the teeth (i.e., the periodontium)8.” WHY 8 references in the first paragraph? Reduce it for 3.**

**Reviewer #3 – Introduction Suggestions**

**Response:**

We thank the reviewer for this insightful suggestion. We revised the Introduction to better emphasize that periodontitis is a dynamic disease characterized by progressive tissue destruction over time, whereas most current diagnostic tools, particularly radiography, provide static assessments and are relatively insensitive to small longitudinal changes. We also added discussion regarding the diagnostic window for early intervention and the sensitivity limitations of conventional radiography, including the requirement for substantial mineral loss before defects become visible.

Furthermore, we clarified the role of high-frequency ultrasound as a tool that may bridge this diagnostic gap. Specifically, we added text describing the use of a 40 MHz transducer with an axial resolution of approximately 40 μm, highlighting its potential to detect small longitudinal bone changes beyond the detection capability of conventional radiography. We also added a transition statement emphasizing the need for a high-precision, radiation-free method for monitoring small bone-level changes over time. These revisions are included in the Introduction section of the revised manuscript. We also reduce the number of references for the first part of the introduction as suggested.

**5. MM: it is necessary a structural refinement:
- The SVD innovation: the authors must explicitly state why SVD was used. It wasn't just for "noise reduction" – it was to extract the coherent topographic signal from the "clutter" of speckle noise.

- Mathematical notation: Ensure your SVD equation A=UΣVT is clearly defined as the "economy-size" decomposition to show computational efficiency.**

**Response**

We thank the reviewer for this important suggestion. The Materials and Methods section has been revised to clarify the role of Singular Value Decomposition (SVD) in the ultrasound image processing workflow. Specifically, SVD was used not merely for noise reduction, but to separate coherent structural signals (such as the alveolar bone surface and enamel interfaces) from incoherent speckle noise and frame-to-frame variability. By decomposing the image stack into spatial and temporal components, high-energy singular values corresponding to coherent anatomical structures were retained, while lower-energy components associated with random noise and speckle clutter were removed using a low-rank approximation.

We also expanded the mathematical description of the decomposition and explicitly defined the SVD formulation $A=U\Sigma V^{T}$, where the matrix $A$represents the vectorized image stack. Additionally, we clarified that the economy-size SVD decomposition was used to improve computational efficiency and reduce memory usage while preserving the dominant spatial components required for image compounding and noise suppression.

These clarifications have been added to the Materials and Methods section.

The method as shown in (COED #1) has the following processing steps for (Auto-SVD-US):

**Multi-frame acquisition and preprocessing.**

For each examination stage, four sequential ultrasound frames were acquired from an approximately identical region of interest (ROI) centered on the target tooth, acknowledging the practical constraint of perfect repositioning. All frames underwent manual ROI selection to ensure consistent anatomical coverage. The system standardized frame dimensions through an automated center-cropping and zero-padding algorithm to maintain spatial consistency. Each frame was converted to double-precision format, normalized, and any non-finite values were handled through zero-replacement to ensure mathematical integrity for subsequent processing.

**Rigid registration and frame alignment**.

To establish a common spatial framework, an intensity-based rigid registration was performed. The first frame served as the reference. Using a mean squares metric and a regular step gradient descent optimizer (300 maximum iterations), each subsequent frame was transformed to align with the reference. The selection of 300 iterations was empirically determined to balance computational efficiency and processing time with registration accuracy, ensuring robust convergence across diverse image qualities. This step corrected for global translational and rotational shifts, ensuring that the alveolar bone structures were spatially congruent across all four frames before compounding.

**SVD filter for noise suppression and image compounding**.

To suppress uncorrelated noise and enhance the coherent topographic signal, the stack of four registered frames was processed using Singular Value Decomposition (SVD) [31, 32]. The procedure was as follows: First, the image stack was reshaped into a two-dimensional matrix $A$ of dimensions (pixels) × (frames). Specifically, with an image resolution of h (height) × w (width) and four frames, the matrix $A$ was constructed by vectorizing each frame, resulting in dimensions of (h × w) × 4. Each column of $A$ represented a single frame vectorized into a one-dimensional array.

This matrix was then decomposed using an economy-size SVD, factorizing it into three constituent matrices:

$$A=U \Sigma V^{T}$$

Where: $U$ is an (h × w) × 4 orthogonal matrix whose columns represent spatial components, or eigen images, of the input data. $\Sigma$ is a 4×4 diagonal matrix containing the singular values ($\sigma_{1}$ to $\sigma_{4}$) in descending order. These values quantify the energy or significance of each corresponding spatial component in $U$. $V^{T}$ is a 4×4 orthogonal matrix whose rows represent the temporal components, describing how the spatial modes are weighted across the different frames.

A low-rank approximation was applied to filter the data. This was achieved by retaining only the most significant singular values, which correspond to the coherent bone topography, while discarding lower-value components associated with random noise and residual artifacts. **For this analysis, the singular value matrix was truncated to retain only the first two components, preserving the primary spatial signal of the anatomy while effectively suppressing noise-dominated higher-order components.**

The filtered matrix $A^{'}$ was reconstructed via $A^{'}=U*\Sigma^{'}*V^{T}$ . where $\Sigma^{'}$represents the truncated singular value matrix. Finally, this matrix was reshaped back into its original spatial dimensions (h × w). The final, SVD-compounded image for the stage was extracted as the first spatial component (the first column of $U$), which provides the purest representation of the bone topography with effectively suppressed noise.

In ultrasound imaging, speckle noise is largely uncorrelated between frames, whereas structural anatomical features such as bone and enamel interfaces remain spatially coherent; therefore, SVD allows separation of coherent anatomical structure from incoherent speckle clutter through low-rank approximation.

**6. Results & Discussion:
- In your discussion, you must address the "Precision vs. Accuracy" paradox you found: Precision (high): excellent for tracking change over time; Accuracy (variable): Challenging for single absolute measurements.

- The pitch: argue that in periodontology, progression (delta) is more clinically significant than a single baseline value. This justifies why HFUS is the perfect "monitoring" tool even if it doesn't replace the initial radiographic survey.

Key Revisions Checklist for Top-Tier Submission

Statistical rigor: Ensure Table A1 (Bland-Altman) includes Proportion of Bias. If the bias is only -18 μm on a 200 μm change, that is a <10% error – a huge selling point.
- Explicitly mention that Method 2 (Auto-SVD) removes the need for fiducial markers (steel balls), making it the "clinically viable" path.**

**Response** Precision vs. Accuracy

We thank the reviewer for this insightful and important comment. We have revised the Discussion to explicitly address the “precision versus accuracy” distinction and its clinical implications.

Specifically, we clarified that while absolute accuracy of ultrasound measurements may be limited due to variability and reference differences, the system demonstrates high precision, low system error (77–113 µm), and a small detection threshold (~138 µm), enabling reliable detection of small incremental changes.

We strongly thank the reviewer for his helpful high lighting of a very important “Pitch argument”. We further emphasized that, in periodontal disease management, the detection of progression (i.e., longitudinal changes over time) is more clinically meaningful than the determination of a single absolute baseline measurement. This explains why moderate agreement with reference methods does not limit the clinical value of the technique.

Accordingly, we clarified that high-frequency ultrasound (HFUS) is not intended to replace radiographic assessment for initial absolute bone level evaluation, but rather to complement it as a radiation-free tool for monitoring disease progression over time. These revisions strengthen the interpretation of HFUS as a precise longitudinal monitoring tool, consistent with the reviewer’s suggestion.

We thank the reviewer for this important comment. We have revised Table A1 (Bland–Altman analysis) to include the proportion of bias relative to a typical bone loss increment. Specifically, the best-case bias of −18 µm corresponds to approximately 9% of a 200 µm change, highlighting the low relative error and strengthening the clinical relevance of the method for detecting small longitudinal changes.

In addition, we have explicitly clarified in the Methods and Discussion that the automated SVD-based approach (Method 2) eliminates the need for fiducial markers (steel balls). This represents a key step toward clinical translation, as it enables measurement using anatomical landmarks (e.g., CEJ) without reliance on external markers, thereby supporting its viability as a clinically applicable workflow.

**Agreement between ultrasound and photographic ground truth**

The agreement between ultrasound-based measurements and the photographic ground truth was comprehensively assessed using Bland-Altman analysis across all measurement scenarios and examiner pairs.

**Primary method comparisons**

Across all modalities, ultrasound-based methods showed agreement with camera-based measurements (Table A1), as illustrated in Fig 4. Panels (e-g) show Bland-Altman plots for the three primary ultrasound methods (Manual-BO-US, DTS-US, Auto-SVD-US) compared to the manual camera ground truth, while panels (h-j) show the same comparison broken down by individual examiner for the Manual-BO-US method. The Auto-SVD-US vs Manual-BO-CA comparison demonstrated the smallest bias was –18 µm, indicating no systematic over- or under-estimation. The best-case bias of −18 µm corresponds to less than 10% error relative to a typical 200 µm bone change increment, indicating clinically acceptable agreement for longitudinal monitoring applications. However, the limits of agreement were -418 µm to 381 µm, representing a range of 799 µm**.** This wide range indicates that for any single measurement, the difference between this automated ultrasound method and the photographic standard could be substantial. The DTS-US vs Manual-BO-CA comparison showed a slightly larger bias of 86 µm, with limits of agreement between –336 µm to 508 µm, with a range of 844 µm. The Manual-BO-US vs Manual-BO-CA comparison showed a bias of 30 µm corresponds to 15% error relative to a typical 200 µm bone change increment and limits of agreement between –307 µm to 369 µm, with a range of 676 µm.

**S1. Table A1. Comprehensive Bland-Altman agreement statistics**

| **Comparison** | **Bias (µm) or %** | **Lower LOA (µm)** | **Upper LOA (µm)** | **Comparison** | **Bias (µm) or %** | **Lower LOA (µm)** | **Upper LOA (µm)** |
| --- | --- | --- | --- | --- | --- | --- | --- |
| **Primary Method Comparisons (Ultrasound vs Camera)** | | | | **Primary Ultrasound Method Comparisons** | | | |
| **Manual-BO-US vs Manual-BO-CA (All examiners)** | 30/15% | -307 | 369 | **DTS-US vs Manual-BO-US** | 138/69% | -391 | 667 |
| **DTS-US vs Manual-BO-CA** | 86/43% | -336 | 508 | **US-Auto vs Manual-BO-US** | 34/17% | -430 | 497 |
| **Auto-SVD-US** **vs Manual-BO-CA** | -18 / 9% | -418 | 381 | **Auto-SVD-US** **vs DTS-US** | 105/52.5% | -225 | 435 |
| **Manual-BO-US vs Auto-CA** | 67/33.5% | -360 | 495 | **Primary Camera Method Comparisons** | | | |
| **DTS-US vs Auto-CA** | 71/35.5% | -288 | 430 | **Manual-BO-CA vs Auto-CA** | 15/7.5% | -330 | 360 |
| **Auto-SVD-US** **vs Auto-CA** | -33/16.5% | -379 | 312 |  |  |  |  |
| **Inter-Observer CA (Manual)** | | | | **Ultrasound vs Camera (Manual blind examiners)** | | | |
| **Ex1 vs Ex2 Manual-BO-CA** | 58/29% | -430 | 546 | **Ex1 (Manual-BO-US Vs Manual-BO-CA)** | 14/7% | -440 | 470 |
| **Ex1 vs Ex3 Manual-BO-CA** | -69/34.5% | -875 | 736 | **Ex2 (Manual-BO-US Vs Manual-BO-CA)** | -60/30% | -530 | 410 |
| **Ex2 vs Ex3 Manual-BO-CA** | -127/63.5% | -943 | 689 | **Ex3 (Manual-BO-US Vs Manual-BO-CA)** | 107/53.5% | -563 | 778 |
| **Inter-Observer US (Manual)** | | | |  | | | |
| **Ex1 vs Ex2 Manual-BO-US** | 17/8.5% | -410 | 443 |  |  |  |  |
| **Ex1 vs Ex3 Manual-BO-US** | -24/12% | -650 | 602 |  |  |  |  |
| **Ex2 vs Ex3 Manual-BO-US** | -40/20% | -531 | 451 |  |  |  |  |

**Discussion**

**Novelty and Contribution to the field**

While several previous studies have demonstrated the feasibility of using high-frequency ultrasound for periodontal and peri-implant imaging [13-17], most have focused on visualization and single-time-point measurements of bone levels or bone thickness. The novelty of this study does not lie in demonstrating that ultrasound can image the alveolar bone, but rather in establishing the quantitative measurement performance required for clinical monitoring of periodontal bone loss progression. Specifically, this study quantifies measurement precision, determines the minimum detectable bone loss change, and evaluates the capability of high-frequency ultrasound for longitudinal monitoring of small bone-level changes. This framework shifts the role of HFUS from a purely imaging modality to a quantitative monitoring tool for periodontal disease progression. In periodontal disease management, the detection of progression (i.e., changes over time) is more clinically relevant than the determination of a single absolute bone level. This explains why HFUS, despite limitations in absolute agreement, is particularly well suited as a monitoring tool for longitudinal assessment rather than a replacement for initial radiographic evaluation.

**Methodological novelty and experimental design**

In addition, this study addresses an important experimental challenge in ex vivo validation by introducing fiducial steel ball markers to ensure that measurements were obtained from the exact same anatomical location across different bone loss stages, identified by their characteristic ring-down echo in ultrasound images (Figs 1–3). These markers, together with a voice control command on the Vevo system to eliminate vibrations and the use of a hydrogel pad to improve acoustic coupling and image clarity, enabled accurate determination of system error (77–113 µm) and the empirical detection threshold (138 µm), and provided a reliable spatial reference for validating automated measurement methods.

Importantly, the novel automated CEJ-based measurement methods particularly the SVD-based approach were subsequently evaluated without relying on the fiducial markers, demonstrating that automated measurements can achieve comparable precision while using clinically relevant anatomical landmarks. This distinction is important because the fiducial markers were used only for experimental validation, whereas clinical measurements would rely on anatomical landmarks such as the cementoenamel junction and the enamel surface.

Together, these contributions establish a quantitative framework for evaluating high-frequency ultrasound as a tool for longitudinal periodontal bone loss monitoring.

**Reviewer #3 – Statistics (Bias percentage)**

**Response**

We thank the reviewer for this suggestion regarding statistical interpretation. We added additional explanation of the bias magnitude relative to the measured change and clarified the proportional error interpretation in the Results/Appendix section at **“Primary Method Comparisons” section.**

**Reviewer #3 – Auto-SVD removes markers**

**Response**

We thank the reviewer for highlighting this important point. We clarified in the manuscript that the automatic SVD-based method eliminates the need for fiducial markers, which were only used for manual validation in the ex vivo model. The automatic CEJ-based method represents the clinically applicable workflow and is therefore the primary pathway for clinical translation. We added sentences in discussion.

Importantly, the novel automated CEJ-based measurement methods particularly the SVD-based approach were subsequently evaluated without relying on the fiducial markers, demonstrating that automated measurements can achieve comparable precision while using clinically relevant anatomical landmarks. This distinction is important because the fiducial markers were used only for experimental validation, whereas clinical measurements would rely on anatomical landmarks such as the cementoenamel junction and the enamel surface.

Together, these contributions establish a quantitative framework for evaluating high-frequency ultrasound as a tool for longitudinal periodontal bone loss monitoring.

**7. References: due to the topic studied, ensure you cite recent (2023-2025) AI-segmentation papers to show how your SVD approach complements current trends.**

**Reviewer #3 – References (AI segmentation papers)**

**Response**

We thank the reviewer for this suggestion. We have added recent references related to AI-based segmentation and automated ultrasound image analysis to better position our SVD-based approach within current digital dentistry and automated imaging analysis research trends.

We thank the reviewer #3 again for the insightful comments and constructive suggestions, which helped improve the clarity, structure, and clinical positioning of this proof-of-concept study.

8. Cui Z, Fang Y, Mei L, Zhang B, Yu B, Liu J, et al. A fully automatic AI system for tooth and alveolar bone segmentation from cone-beam CT images. Nat Commun. 2022;13:2096. https://doi.org/10.1038/s41467-022-29637-2

9. Wu D, Jiang J, Wang J, Bi Z, Yu G. Segmentation algorithms of dental CT images: A comprehensive review from classical to deep learning trend. Expert Syst Appl. 2025;275:126853. https://doi.org/10.1016/j.eswa.2025.126853

10. Kazimierczak N, Sultani N, Chwarścianek N, Krzykowski S, Serafin Z, Ciszewska A, et al. AI-based detection of dental features on CBCT: Dual-layer reliability analysis. Diagnostics (Basel). 2025;15(24):3207. https://doi.org/10.3390/diagnostics15243207

11. Ismail IN, Subramaniam PK, Chi Adam KB, Ghazali AB. Application of artificial intelligence in cone-beam computed tomography for airway analysis: A narrative review. Diagnostics (Basel). 2024;14(17):1917. https://doi.org/10.3390/diagnostics14171917

12. Kazimierczak N, Sultani N, Chwarścianek N, et al. Detection accuracy of an AI platform for dental treatment features on panoramic radiographs—tooth- and patient-level analyses. Sci Rep. 2026;16:2436. https://doi.org/10.1038/s41598-025-32226

**Reviewer #4:**

**High frequency ultrasonography (HFUS) for alveolar bone loss is an emerging, non-invasive imaging technique being explored as an alternative or adjunct to radiographs in periodontal diagnosis. It allows real-time visualization of the alveolar crest, buccal bone thickness, and soft tissues without radiation. HFUS for periodontal and peri-implant bone assessment has been actively researched by several ex vivo and clinical studies. The preclinical diagnostic accuracy studies have already showed good agreement between HFUS and imaging for crestal bone thickness and alveolar bone distance measurements repeatedly. While this study is well executed, it does not provide sufficient novelty or significance to the diagnosis of periodontal or peri-implant diseases. Larger, standardized clinical trials are needed to fully validate diagnostic thresholds and automated measurement software.**

**Response**

We thank the reviewer for the thoughtful comments and for highlighting the growing body of research on high-frequency ultrasound (HFUS) for periodontal and peri-implant imaging. We agree that HFUS has been investigated in several ex vivo and clinical studies for visualizing periodontal structures and measuring bone thickness and bone levels. However, the primary goal and novelty of this study differ from previous imaging-focused studies.

The novelty of this study does not lie in demonstrating that ultrasound can image alveolar bone, which has been shown in previous studies. Instead, the novelty of this study lies in establishing the quantitative measurement performance and longitudinal monitoring capability required for clinical monitoring of periodontal bone loss progression. The main contributions of this study can be summarized as follows:

1. **This study is not focused on imaging bone**, but on evaluating the measurement performance of ultrasound for bone loss monitoring.
2. **Quantification of measurement precision (system error)** for multiple ultrasound measurement methods under unchanged conditions.
3. **Experimental determination of the minimum detectable bone loss change of (138 µm)** based on detection performance rather than only theoretical statistical estimates.
4. **Evaluation of longitudinal monitoring capability**, which is essential for detecting small bone changes over time rather than large static defects.
5. **Development of an ex vivo fiducial marker validation method** using steel ball markers to ensure measurements were obtained from the exact same anatomical location across bone loss stages, enabling accurate system error determination and ground truth comparison.
6. **Validation of automated CEJ-based measurement methods without fiducial markers**, demonstrating that clinically applicable automated measurements can achieve comparable precision without external markers.

To our knowledge, most previous HFUS periodontal studies have focused primarily on visualization, anatomical measurements, or single-time-point assessments, whereas this study focuses on measurement precision, detection capability, validation methodology, and longitudinal monitoring performance. These aspects are essential for determining whether HFUS can be used for monitoring periodontal disease progression over time rather than only for static measurements.

The significance of this study lies in its contribution toward enabling future clinical periodontal research by establishing a reliable and reproducible reference methodology. This approach facilitates the translation of findings into more challenging in vivo studies.

We agree with the reviewer that larger, standardized clinical trials will be necessary to fully validate diagnostic thresholds and automated measurement workflows. Therefore, the importance of this study is positioned as a methodological validation and proof-of-concept study designed to establish measurement precision, detection capability, and longitudinal monitoring performance prior to clinical translation. We have clarified this positioning and the novelty of the study in the revised Introduction and Discussion sections.

**Discussion**

**Novelty and Contribution to the field**

While several previous studies have demonstrated the feasibility of using high-frequency ultrasound for periodontal and peri-implant imaging [13-17], most have focused on visualization and single-time-point measurements of bone levels or bone thickness. The novelty of this study does not lie in demonstrating that ultrasound can image the alveolar bone, but rather in establishing the quantitative measurement performance required for clinical monitoring of periodontal bone loss progression. Specifically, this study quantifies measurement precision, determines the minimum detectable bone loss change, and evaluates the capability of high-frequency ultrasound for longitudinal monitoring of small bone-level changes. This framework shifts the role of HFUS from a purely imaging modality to a quantitative monitoring tool for periodontal disease progression. In periodontal disease management, the detection of progression (i.e., changes over time) is more clinically relevant than the determination of a single absolute bone level. This explains why HFUS, despite limitations in absolute agreement, is particularly well suited as a monitoring tool for longitudinal assessment rather than a replacement for initial radiographic evaluation.

**Methodological novelty and experimental design**

In addition, this study addresses an important experimental challenge in ex vivo validation by introducing fiducial steel ball markers to ensure that measurements were obtained from the exact same anatomical location across different bone loss stages, identified by their characteristic ring-down echo in ultrasound images (Figs 1–3). These markers, together with a voice control command on the Vevo system to eliminate vibrations and the use of a hydrogel pad to improve acoustic coupling and image clarity, enabled accurate determination of system error (77–113 µm) and the empirical detection threshold (138 µm), and provided a reliable spatial reference for validating automated measurement methods.

Importantly, the novel automated CEJ-based measurement methods particularly the SVD-based approach were subsequently evaluated without relying on the fiducial markers, demonstrating that automated measurements can achieve comparable precision while using clinically relevant anatomical landmarks. This distinction is important because the fiducial markers were used only for experimental validation, whereas clinical measurements would rely on anatomical landmarks such as the cementoenamel junction and the enamel surface.

Together, these contributions establish a quantitative framework for evaluating high-frequency ultrasound as a tool for longitudinal periodontal bone loss monitoring.

**Fig 1. The alveolar bone crest is visualized with photography vs ultrasound.** (a) A normal view of the Canon camera captures a fixed pig jaw. The alveolar bone was exposed by reflecting the overlying gingiva. Two steel balls were fixed on each tooth site as fiducial markers. (b) A cropped image section frame of tooth PM4 under photographic camera showing the yellow double arrow between the 1^st^ steel ball and the bone crest aligned with the dashed red line which is the tested comparable plane between the two fixed balls, (c) The corresponding ultrasound frame at B-mode section frame scanned by Vevo F2 with a 40 MHz ultrasound transducer, the distance of yellow double arrow between the 1^st^ steel ball and the bone crest ABC was measured. The distance of the red double arrow in (c) is corresponding with the distance of the yellow double arrow in (b). The fixed steel ball can be distinguished by a ring down echo as shown clearly at the ultrasound scan. (d) is a schematic diagram of a cross-section profile with no bone loss. Panels (e) and (f) represent the alveolar bone crest of tooth M3 following six stages of alveolar bone mechanical removal. (e) Photographic frames captured by a Canon camera. (f) Ultrasound scanning using a 40 MHz ultrasound array transducer, where the green dashed line is the alveolar bone crest level before bone removal. ABC (No loss): alveolar bone crest level with no bone loss, T: tooth enamel, B: alveolar bone, gingiva margin GM, and G: gingiva. The length of the yellow lines at panels (e) and (f) is the change in the amount of bone level detected at different alveolar bone loss stages. The fixed steel balls can be distinguished in panel (f) by a ring down echo as shown clearly at the ultrasound scan.

#### **Fig 2. Ultrasound-based assessment of alveolar bone loss using DTS-US method (at molar M2).** Panels (**a)** and (**b)** represent the raw ultrasound frames at the 2nd and 3rd loss stages, the fixed steel ball can be distinguished by a ring down echo as shown clearly at the ultrasound scan. Panels (**c)** and (**d)** represent the frame superposition method. Consecutive frames are overlaid to measure the displacement of the alveolar bone crest (ABC) between both frames. In **(c)**, red/green overlays demonstrate frame registration and displacement tracking across stages (2nd and 3rd loss stages). The tooth enamel surface and cemento-enamel junction “CEJ” coincide, while the shift between ABCs directly quantifies bone loss measurement. In **(d)**, the raw intensity difference map between consecutive frames confirms optimal overlay adjustment and highlights the ABC displacement. The gingiva surface shows high intensity due to different separated **gingiva** positions at different stages and the pressure force difference of the handheld transducer on the gingiva surface. **Panel (e)** shows a flowchart of the two quantification methods for bone loss measurement: DTS-US and Auto-SVD-US. Anatomical landmarks: gingiva “G”, gingiva margin tooth “GM”, enamel “T”, alveolar bone “B”, cemento-enamel junction “CEJ”, in red at raw images (**a)** and (**b)**, and in white at superposition images (**c)** and (**d**), alveolar bone crest “ABC” in yellow.

…….

**Study strengths**

A major strength of this study is the use of a controlled ex vivo model with incremental bone removal and precise photographic ground truth measurements, which allowed accurate quantification of system error, agreement, and minimum detectable change. In addition, multiple measurement approaches were evaluated, including manual, semi-automated, and fully automated ultrasound methods, providing a comprehensive comparison of performance, reliability, and measurement precision across different analysis strategies. The study design incorporated repeated measurements, blinded observers, and automated algorithms, enabling robust evaluation of reliability, agreement, and detection performance. Importantly, this study focused not only on measurement accuracy but also on system error, minimum detectable change, and empirical detection thresholds, which are clinically relevant parameters for longitudinal monitoring. This controlled experimental design enabled a systematic evaluation of the performance characteristics of high-frequency ultrasound for detecting and monitoring small longitudinal changes in alveolar bone loss.

We thank the reviewer #4 for the insightful comments, which helped us better clarify the novelty and positioning of this work.

**Reviewer #5:**

**The manuscript is scientifically sound and methodologically robust. The ex vivo model is appropriate for a proof-of-concept study, and the authors apply multiple complementary measurement strategies, which strengthens internal validity.
My main concern relates to interpretative balance. The detection capability is promising. However, the limits of agreement reported in the Bland–Altman analyses are relatively wide, suggesting that the technology may be better suited for longitudinal monitoring of changes over time rather than direct substitution of photographic reference methods for absolute measurements. This distinction should be stated more explicitly in the Discussion.
Overall, the manuscript meets the journal’s standards for methodological rigor. The requested revisions aim to improve clarity and transparency rather than address fundamental flaws.**

Response:

We thank the reviewer for the positive evaluation of the manuscript and for recognizing the methodological rigor and the appropriateness of the ex vivo proof-of-concept model. We appreciate the reviewer’s insightful comments regarding interpretative balance, statistical clarity, and clinical translation. We have revised the manuscript accordingly to improve clarity, transparency, and clinical interpretation of the results.

**1. Clear differentiation of statistical concepts: system error, minimum detectable change, and empirical detection threshold.
In the Methods (pp. 16–17) and Results (p. 20), the manuscript introduces multiple related statistical metrics:
System error (Mean + 2SD of baseline), minimum detectable change, empirical detection threshold (~138 µm).
While each appears methodologically justified, the distinctions between these concepts are not fully explicit. This may lead to interpretative ambiguity.
Please:
Provide clear definitions of each metric.
Explain how they relate conceptually.
Clarify which metric should be interpreted as the clinically meaningful threshold for longitudinal monitoring.
Explicitly state whether the minimum detectable change was calculated using the conventional formula (e.g., 1.96 × √2 × SD) or whether the threshold reported is purely empirical.
A clearer statistical framework will substantially improve transparency.**

## Response— Statistical Concepts (System error, MDC, detection threshold)

We thank the reviewer for this important comment regarding clarification of statistical terminology. We have revised the manuscript to clearly define and differentiate between system error, minimum detectable change (MDC), and the empirical detection threshold. System error was defined as the measurement variability under unchanged conditions and was calculated as Mean + 2SD of repeated baseline measurements. MDC represents a theoretical statistical threshold derived from measurement variability, whereas the empirical detection threshold of (138 µm) was determined experimentally as the smallest bone loss increment that was consistently detected above the system error threshold across measurement methods. We clarified the relationship between these parameters in the Methods, Results, and Discussion sections and added explanatory notes to Table 5 to improve clarity and interpretability.

In this study:

- **System error** was defined as the measurement variability under unchanged conditions at baseline and was calculated as Mean + 2SD of repeated baseline measurements. This represents the noise threshold of the measurement system.
- **Minimum detectable change (MDC)** represents the smallest change that can be confidently distinguished from measurement error and is conceptually related to system error.
- The reported **empirical detection threshold (138 µm)** was determined experimentally as the smallest bone loss increment that was consistently detected above the system error threshold across measurement methods.

We clarified that the reported detection threshold is primarily empirical and based on experimental detection performance rather than solely derived from the theoretical MDC formula. The relationship between these statistical concepts has now been clarified in the revised manuscript to improve interpretability and transparency (added under “Definition of System Error” and clarified in the Results and Discussion sections).

**Data and statistical analysis**

**Definition of system error**

The inherent precision of each measurement system, defined as the system error, was determined from repeated measurements under unchanged conditions at the baseline (“no loss”) stage. For each method, the apparent “bone loss” was calculated between replicate measurements where no anatomical changes occurred. The system error was calculated as the mean of these apparent changes plus two standard deviations (Mean + 2SD), establishing a one-sided upper threshold for measurement noise corresponding to the 97.5th percentile under a normal distribution. Baseline measurement error distributions were visually inspected using histograms and were found to be approximately normally distributed; therefore, the Mean + 2SD threshold criterion was considered appropriate. Since bone loss is a unidirectional process, this one-sided threshold was used as the statistical criterion, and any measured change in subsequent stages exceeding this threshold was considered a true-positive detection of bone loss.

For clarity, three related statistical concepts were defined in this study. **System error** represents the inherent variability of a measurement method under unchanged conditions and defines the noise floor of the measurement system. The **minimum detectable change (MDC)** represents the smallest change that can be distinguished from measurement variability with statistical confidence and is a theoretical statistical threshold derived from system variability. In contrast, the **empirical detection threshold** was determined experimentally as the smallest actual bone loss increment that was consistently detected above the system error threshold across incremental bone loss stages. Any measured change in subsequent stages exceeding this threshold was considered a true-positive detection of bone loss.

**2. Interpretation of regression analyses (R² values)
In the regression analyses comparing ultrasound-based measurements with photographic ground truth (p. 18), R² values range approximately between 0.41 and 0.55.
Although statistically significant, these values indicate moderate correlation.
Please expand the discussion to address:
The methodological meaning of these coefficients.
How moderate R² values should be interpreted in light of low mean bias.
Whether these correlation levels support clinical interchangeability or rather reinforce the role of HFUS as a longitudinal monitoring tool.
The manuscript would benefit from a more explicit and balanced interpretation of these findings.**

We thank the reviewer for this important and insightful comment. The Discussion has been revised to provide a more explicit and balanced interpretation of the regression results.

Specifically, we clarified the methodological meaning of the reported R² values (0.41–0.55), emphasizing that these coefficients reflect the strength of linear association and measurement dispersion rather than agreement between methods. We explicitly stated that R² does not assess interchangeability, which is more appropriately evaluated using Bland–Altman analysis.

We further expanded the discussion to explain how moderate R² values can coexist with low mean bias. We clarified that the relatively low bias observed indicates limited systematic error, while the moderate R² values primarily reflect random measurement scatter rather than consistent over- or underestimation.

In addition, we explicitly addressed the clinical interpretation of these findings. We clarified that the observed correlation levels do not support direct clinical interchangeability with photographic reference measurements for absolute bone level assessment. Instead, the combination of low systematic bias, system error (77–113 µm), and a small empirical detection threshold (~138 µm) supports the role of high-frequency ultrasound as a tool for longitudinal monitoring of alveolar bone changes.

These revisions provide a clearer distinction between correlation and agreement and strengthen the interpretation of HFUS as a precise method for detecting relative changes over time rather than a replacement for absolute measurement techniques.

We clarify that at new subtitle in our manuscript**/ Discussion/**

**: Agreement analysis and the role of precision versus accuracy**

**Agreement analysis and the role of precision versus accuracy**

The regression analyses demonstrated moderate correlation coefficients (R² ≈ 0.41–0.55) between ultrasound measurements and the photographic ground truth. While these values are statistically significant, they indicate only moderate linear association and reflect variability in measurement dispersion rather than direct agreement between methods. Importantly, R² quantifies the strength of correlation but does not assess measurement agreement or interchangeability.

Despite the moderate R² values, the relatively low mean bias observed in the Bland–Altman analysis indicates that systematic error is limited and that the primary source of variability arises from random measurement scatter rather than consistent over- or underestimation. Ultrasound measurements did not consistently overestimate or underestimate bone levels but instead showed variability around the reference measurements. This distinction explains how moderate correlation can coexist with low bias.

The limits of agreement observed in the Bland–Altman analysis indicate that ultrasound measurements should not be considered interchangeable with photographic reference measurements for absolute bone level determination. However, periodontal disease management is primarily concerned with detecting changes in bone levels over time rather than determining a single absolute measurement. In this context, the low system error (77–113 µm) and the small empirical detection threshold (~138 µm) demonstrate high precision in detecting longitudinal changes above the measurement noise floor.

Therefore, the ability of high-frequency ultrasound to detect small changes relative to baseline with low systematic bias is more clinically relevant than achieving high absolute agreement with a reference method. These findings highlight an important distinction between accuracy and precision: while absolute measurements show variability, the system demonstrates high precision for monitoring change over time. Overall, high-frequency ultrasound is best positioned as a longitudinal monitoring tool for tracking small changes in alveolar bone levels, rather than serving as a direct replacement for radiographic methods used for absolute measurements or initial diagnosis.

The limits of agreement (approximately 700–800 µm) are substantially larger than the empirically determined detection threshold (~138 µm), indicating that while absolute measurements exhibit considerable variability, the system retains high sensitivity for detecting small longitudinal changes above the noise floor. Importantly, clinically meaningful changes in periodontal bone levels are typically on the order of 100–200 µm in longitudinal monitoring, indicating that the detection threshold of ~138 µm falls within this clinically relevant range.

This disparity highlights an important distinction between absolute accuracy and changes detection capability. The relatively wide limits of agreement reflect variability in single-time-point measurements and therefore limit the use of HFUS for precise absolute bone level determination. However, the much smaller detection threshold demonstrates that the system is capable of consistently identifying clinically relevant incremental changes over time.

From a clinical perspective, periodontal disease monitoring primarily relies on detecting progression rather than establishing a single absolute bone level measurement. In this context, the ability to reliably detect changes exceeding the system error threshold is more meaningful than achieving tight agreement with a reference method. Therefore, despite the wide limits of agreement, the measurement characteristics of HFUS support its role as a longitudinal monitoring tool, where relative changes from baseline are of primary importance.

**3. Clinical implications of Bland–Altman limits of agreement
The Bland–Altman analyses (p. 19) demonstrate limits of agreement approaching approximately 700–800 µm in certain comparisons.
While acknowledged, the clinical implications of these intervals require clearer contextualization.
Please:
Discuss whether these intervals allow true interchangeability with photographic reference measurements.
Explicitly differentiate between suitability for absolute measurement and suitability for change detection over time.
Clarify how these limits compare to clinically meaningful thresholds in periodontal assessment.
This distinction is essential for appropriate interpretation.**

# Comment 3 — Bland–Altman Limits of Agreement

## Response

We thank the reviewer for this important comment regarding the clinical interpretation of the Bland–Altman limits of agreement. We have expanded the Discussion to clarify that the relatively wide limits of agreement indicate that HFUS measurements may not be directly interchangeable with photographic reference measurements for single absolute bone level measurements. However, the low mean bias and high repeatability indicate that HFUS is well suited for detecting longitudinal alveolar bone changes over time. We clarified that HFUS should be interpreted as a monitoring tool for disease progression rather than a direct replacement for reference measurement methods.

We also expanded the Discussion to quantitatively compare the limits of agreement (~700–800 µm) with clinically meaningful thresholds, including the empirically determined detection threshold (~138 µm). This comparison clarifies that although absolute measurements exhibit substantial variability, the system maintains high sensitivity for detecting small longitudinal changes, which is the clinically relevant objective in periodontal monitoring.

In addition, we clarified that clinical decision-making relies on reproducible quantitative measurements rather than visual estimation. While photographic reference images may appear visually precise, clinicians cannot rely on visual assessment alone. Therefore, the ability of HFUS to provide consistent and precise measurements for detecting changes over time is more clinically meaningful than achieving strict absolute agreement with reference images.

**Agreement analysis and the role of precision versus accuracy**

From a clinical perspective, periodontal disease monitoring primarily relies on detecting progression rather than establishing a single absolute bone level measurement. Importantly, clinical decision-making relies on reproducible quantitative measurements rather than visual estimation alone. While photographic reference images may appear visually precise, clinicians cannot directly measure bone levels by visual inspection, and therefore consistent detection of changes over time is more clinically meaningful than strict agreement with a reference image. In this context, the ability to reliably detect changes exceeding the system error threshold is more meaningful than achieving tight agreement with a reference method. Therefore, despite the wide limits of agreement, the measurement characteristics of HFUS support its role as a longitudinal monitoring tool, where relative changes from baseline are of primary importance.

**4. Algorithm transparency and reproducibility
The Auto-SVD-US method (pp. 13–16) is described in detail, which is commendable.
To enhance reproducibility:
Clearly specify which algorithm parameters are fixed and which are user-defined.
Indicate whether the implementation code (e.g., MATLAB scripts) or pseudocode will be made available.
If parameter tuning was performed, describe the selection process.
This will strengthen methodological transparency.**

# Comment 4 — Algorithm Transparency

## Response

We thank the reviewer for highlighting the importance of reproducibility and algorithm transparency. We clarified in the Methods section which algorithm parameters were fixed, and which were user-defined. The SVD rank selection, registration parameters, and ROI selection procedures have been described more explicitly. We also clarified that the algorithms were implemented in MATLAB, and we will make the code or pseudocode available upon reasonable request as it is available for the reviewers at Gethub (Please check the note bellow) to improve reproducibility and transparency. More clear sentences added under **Materials and methods / Automated measurement algorithms and** (COED #1 at supporting Information)

**Important note:**

We have uploaded a copy of our MATLAB GUI code used for alveolar bone loss ultrasound image analysis entitled
**“Quantitative-Alveolar-bone-Assessment-using-HFUS-GUI-AlveolarBoneLoss_Analysis_GUI”** to **GitHub** as part of the supplementary materials.

A detailed user manual has also been uploaded as
**“User Manual – Alveolar Bone Loss GUI.pptx.”**

**Access link: (please copy and paste in any browser)**

<https://github.com/DrTamer/Alveolar-Bone-Loss-GUI>

**access credentials:**

Open for public or available as a supporting document at Plos One.

**Automated measurement algorithms**

Two complementary automated algorithms were developed using technical computing software (MATLAB R2023a; MathWorks, Natick, MA, USA) [30] to provide objective and precise quantification of alveolar bone loss (COED #1). Both methods utilize the CEJ as a stable anatomical reference point, offering a more clinically relevant framework than external markers such as the steel balls used in this study for the blinded observers’ method**. Rather than relying on a deep learning-based approach, which requires many** annotated image datasets**, we developed a distinct methodology by optimizing traditional image processing techniques for 40 MHz HFUS data. This approach offers transparency and ease of use, making it suitable for our proof-of-concept study. This included the application of Singular Value Decomposition (SVD) filtering for noise reduction [31,32] and intensity-based registration for alignment, which are particularly suited to address the challenges of speckle noise and subtle hard-tissue interfaces characteristic of this modality.** All algorithms were implemented using technical computing software and its image processing toolbox (MATLAB R2023a with Image Processing Toolbox; MathWorks, Natick, MA, USA) [30] and its Image Processing Toolbox. Fig 2 e shows a flowchart of the two algorithms for measuring the alveolar bone by HFUS, the methods are described in detail in the following sections.

**Data availability**

All relevant data are within the manuscript and its Supporting Information files. The raw measurement data, Bland–Altman analysis data, and statistical analysis datasets are provided as Supporting Information files. The MATLAB code used for automated measurement and image processing is publicly available at the following repository:
<https://github.com/DrTamer/Quantitative-Alveolar-bone-Assessment-using>
These materials are provided to facilitate reproducibility and reuse in accordance with PLOS ONE data and code sharing policies.

**Supporting information**

**S1. Table A1. Comprehensive Bland-Altman agreement statistics**

S2. CODE #1. Alveolar Bone Loss Analysis MATLAB GUI Code:

**5. Justification of the Mean + 2SD threshold
Please indicate whether baseline data distribution was tested for normality before defining system error as Mean + 2SD. If tested, briefly describe the method used.**

# Comment 5 — Mean + 2SD Normality

## Response

We thank the reviewer for this comment regarding the justification of the Mean + 2SD threshold. The baseline measurement error distributions were inspected for approximate normality using histogram inspection prior to applying the Mean + 2SD threshold criterion. The distributions were found to be approximately normally distributed; therefore, the Mean + 2SD approach was considered appropriate for defining the system error threshold. This clarification has been added to the Materials and Methods section under “Definition of System Error.”

**Data and statistical analysis**

**Definition of system error**

The inherent precision of each measurement system, defined as the system error, was determined from repeated measurements under unchanged conditions at the baseline (“no loss”) stage. For each method, the apparent “bone loss” was calculated between replicate measurements where no anatomical changes occurred. The system error was calculated as the mean of these apparent changes plus two standard deviations (Mean + 2SD), establishing a one-sided upper threshold for measurement noise corresponding to the 97.5th percentile under a normal distribution. Baseline measurement error distributions were visually inspected using histograms and were found to be approximately normally distributed; therefore, the Mean + 2SD threshold criterion was considered appropriate. Since bone loss is a unidirectional process, this one-sided threshold was used as the statistical criterion, and any measured change in subsequent stages exceeding this threshold was considered a true-positive detection of bone loss.

For clarity, three related statistical concepts were defined in this study. **System error** represents the inherent variability of a measurement method under unchanged conditions and defines the noise floor of the measurement system. The **minimum detectable change (MDC)** represents the smallest change that can be distinguished from measurement variability with statistical confidence and is a theoretical statistical threshold derived from system variability. In contrast, the **empirical detection threshold** was determined experimentally as the smallest actual bone loss increment that was consistently detected above the system error threshold across incremental bone loss stages. Any measured change in subsequent stages exceeding this threshold was considered a true-positive detection of bone loss.

**6. Expansion of limitations regarding the ex vivo model
Although the experimental model is well described (pp. 10–12), please expand the Discussion to briefly address additional in vivo considerations such as:
Soft tissue variability
Saliva presence
Probe angulation variability
Differences in acoustic impedance in clinical conditions.**

# Comment 6 — Expand Limitations (In Vivo Conditions)

## Response

We thank the reviewer for this suggestion. We expanded the limitations section to include additional in vivo considerations such as soft tissue variability, saliva presence, probe angulation variability, and differences in acoustic impedance in clinical conditions. These factors may affect image quality, landmark visibility, and measurement variability in clinical applications and should be investigated in future in vivo studies.

**Limitations**

This study has several limitations. First, the experiments were performed using an ex vivo swine jaw model without soft tissue inflammation or physiological conditions, which may differ from in vivo imaging conditions and may not fully represent inflammatory bone resorption seen in periodontitis. Second, alveolar bone defects were created using controlled mechanical removal rather than inflammatory bone resorption, which may result in different bone surface morphology compared to clinical bone loss patterns.

Third, the study was performed under controlled experimental conditions with careful probe positioning and alignment, which may differ from clinical scanning conditions where operator variability, saliva, soft tissue thickness, and patient movement may influence image quality and measurement accuracy.

Fourth, steel ball fiducial markers were used only in the *ex vivo* model to establish precise ground truth reference measurements and frame alignment. These markers are not clinically applicable; however, the automated measurement method developed in this study uses the cementoenamel junction (CEJ) as a natural anatomical reference point, which is clinically applicable and eliminates the need for fiducial markers.

Finally, although this study demonstrates measurement precision and detection capability of high-frequency ultrasound, further in vivo studies and clinical trials are required to evaluate clinical performance, reproducibility, and workflow integration.

Despite these limitations, this study establishes a quantitative framework for evaluating measurement precision, system error, and minimum detectable change for longitudinal monitoring of alveolar bone loss using high-frequency ultrasound. In addition, the number of independent bone loss events used for diagnostic performance analysis was limited, and therefore sensitivity and specificity estimates should be interpreted as preliminary.

**7. Translational integration into clinical workflow
Consider adding a brief statement regarding:
Estimated acquisition time per site
Operator training requirements
Practical considerations for clinical implementation**

Comment 7 — Clinical Workflow Integration

## Response

We thank the reviewer for this suggestion regarding clinical translation. We added a brief discussion regarding practical considerations for clinical implementation, including acquisition time per site, operator training requirements, and workflow integration. Ultrasound acquisition for a single site typically requires only a few seconds once probe positioning is achieved, and operator training would primarily involve probe positioning and landmark identification. These practical considerations have been briefly discussed in the revised manuscript.

**Clinical implications and workflow integration**

In summary, the statistical analyses in this study follow a deliberate logical progression: from characterizing the measurement noise floor through system error (Table 2), to establishing detection capability through the empirical threshold of 138 µm and sensitivity/specificity analysis (Tables 4 and 5), to validating agreement and reliability through Bland–Altman analysis (Fig. 4, Table A1) and ICC assessment (Table 3), and finally to confirming correlation through regression analysis (Fig. 4d). This progression demonstrates that the clinical value of HFUS for periodontal monitoring rests not on absolute measurement accuracy where the wide limits of agreement (676–844 µm range) indicate that single measurements should not be considered interchangeable with photographic references but on measurement precision, where the low system error (77–113 µm) and empirical detection threshold of (138 µm) enable detection of longitudinal changes approximately six times smaller than the 870 µm threshold reported for conventional radiography. The core clinical insight is that periodontal disease management depends on detecting changes from a patient's own baseline over time, not on a single absolute bone-level value. The HFUS framework established in this study particularly through the automated DTS-US and Auto-SVD-US workflows provides the precision, reliability, and detection sensitivity required for this task, while eliminating ionizing radiation exposure and enabling more frequent monitoring intervals. This quantitative framework shifts the role of HFUS from a purely imaging modality to a validated measurement tool for longitudinal periodontal bone loss monitoring.

This study establishes that HFUS, particularly via automated algorithms, enables reliable, radiation-free quantification of alveolar bone loss with a sub-millimeter detection capability. Its optimal clinical application is in the safe, repetitive monitoring of disease progression, facilitating a shift from reactive to proactive periodontal care. In a potential clinical workflow, ultrasound acquisition at a single periodontal site requires approximately 10–20 seconds, and operator training primarily involves probe positioning and CEJ identification. With automated processing, the workflow could be integrated into routine periodontal monitoring visits.

Importantly, the detection threshold (~138 µm) lies well below the limits of agreement, reinforcing that the system can detect meaningful changes even in the presence of variability in absolute measurements.

We thank the reviewer #5 for the thorough and thoughtful evaluation of our manuscript and for the constructive recommendations regarding study design description, statistical reporting, methodological transparency, and interpretation of results. We have carefully revised the manuscript to address all points raised. The title, introduction, methods description, statistical justification, results reporting, discussion tone, and conclusions have been revised to improve clarity, methodological transparency, and to better emphasize the experimental proof-of-concept nature of the study. Below we address each point individually.

**Reviewer #6:**

**Thank you for the opportunity to review the manuscript submitted under the title “Quantification of Alveolar Bone Loss Using High-Frequency Ultrasonography for Precision Periodontal Monitoring.” Below, I present my impressions derived from a careful reading of the text, outlining my interpretations and, where appropriate, offering recommendations for the authors’ consideration.

Regarding the title
The submitted article title employs clear, elegant, and technically accurate language, appropriately highlighting the central technology (HFUS) and the clinical context of periodontal monitoring with increased precision, while remaining concise.
However, some limitations warrant reflection. The title may be overly generic for an experimental validation study, omitting essential information for experienced readers and methodological reviewers (such as the sample, comparator, and study design). As a result, it does not fully convey the originality or experimental rigor of the work.**

**Response:**
We agree with the reviewer and revised the title to better reflect the experimental validation and proof-of-concept nature of the study. The revised title now specifies the high-frequency ultrasound system, automated measurement approach, and the proof-of-concept ex vivo validation design.

**Revised title:**

RESEARCH ARTICLE

**Sub-millimeter quantification of alveolar bone loss using automated 40 MHz high-frequency ultrasound: a proof-of-concept *ex vivo* validation study**

Tamer Abdelrehim^1^, Baiyan Qi^1^, Bryant Tran^2^, Mirali Pandya^3^, Jesse V. Jokerst^*1,3,4^, Casey Chen^*2^

**1** Aiiso Yufeng Li Family Department of Chemical and Nanoengineering, University of California San Diego, La Jolla, California, United States of America, **2** Herman Ostrow School of Dentistry, University of Southern California, Los Angeles, California, United States of America, **3** Materials Science and Engineering Program, University of California San Diego, La Jolla, California, United States of America, **4** Department of Radiology, University of California San Diego, La Jolla, California, United States of America

* [ccchen@usc.edu](mailto:ccchen@usc.edu), [jjokerst@ucsd.edu](mailto:jjokerst@ucsd.edu)

The revised title explicitly includes the study design (“proof-of-concept ex vivo validation study”) to reflect the experimental nature and methodological rigor of the work.

**Regarding the introduction and objectives
Overall, the research rationale and literature exploration are adequate and well substantiated, with a clear logical progression and consistent use of references to support the scientific problem.
Suggestion for improvement:
Although the literature is well explored, the introduction could benefit from an explicit sentence comparing HFUS with previous attempts at periodontal ultrasonography, even if only to highlight their limitations (e.g., insufficient frequency, low resolution, lack of quantitative validation). This would further strengthen the originality of the study.
The relevance and contribution of the study are, in general, very well-articulated, both from clinical and technological perspectives, clearly justifying the need for publication.
The formulation and adequacy of the objectives are clear and methodologically aligned with the stated rationale.**

# Comment Group 2 — Introduction (Comparison with previous ultrasound studies)

**Reviewer comment:** The introduction should include a comparison between HFUS and previous periodontal ultrasound studies.

**Response:**
We agree with the reviewer and added a paragraph in the Introduction comparing high-frequency ultrasound used in this study with previous periodontal ultrasound approaches. Specifically, we clarified that earlier ultrasound studies often used lower-frequency systems with limited spatial resolution and focused primarily on qualitative imaging or single time-point measurements. In contrast, the present study uses a 40 MHz high-frequency ultrasound system and focuses on quantitative validation of measurement precision, system error, agreement analysis, and detection capability for small incremental bone loss. This addition helps clarify the novelty and methodological contribution of this study.

We explicitly stated that previous periodontal ultrasound studies often relied on lower-frequency systems with limited spatial resolution and primarily focused on qualitative or single time-point assessments, whereas the present study uses a 40 MHz system and provides quantitative validation of measurement precision and detection capability.

Previous ultrasound studies for periodontal assessment have demonstrated the feasibility of ultrasonography for imaging periodontal and peri-implant structures [16-23]. Early studies using lower-frequency ultrasound systems (e.g., around 20 MHz) were able to visualize periodontal tissues but were limited by insufficient spatial resolution for detecting small bone-level changes [17]. More recent intraoral ultrasound studies have evaluated periodontal structures, crestal bone dimensions, and peri-implant tissues and have demonstrated good repeatability and clinical feasibility [18-23]. Photoacoustic imaging has also been explored for periodontal assessment [24]. However, many of these studies focused primarily on qualitative visualization, anatomical imaging, or single time-point measurements rather than quantitative validation of measurement precision, system error, and minimum detectable bone loss. In addition, earlier ultrasound systems often operated at lower frequencies with lower spatial resolution compared to modern high-frequency ultrasound systems [15, 16, 25]. Therefore, the present study differs from previous work by using a 40 MHz high-frequency ultrasound system and by focusing on quantitative validation of measurement performance, including system error, agreement analysis, and detection capability for small incremental bone loss.

Periodontitis is a dynamic disease characterized by progressive tissue destruction over time, whereas most current diagnostic tools, particularly radiography, provide static assessments and are relatively insensitive to small longitudinal changes. The diagnostic window for early intervention is frequently missed because conventional radiographs require substantial mineral loss before defects become visible. Therefore, a measurement method capable of detecting small longitudinal changes with high precision may be more clinically valuable for monitoring disease progression than methods focused solely on absolute bone level measurements.

# **Regarding the methods A high level of coherence was observed between the introduction, objectives, and the methods presented, with no misalignment between the research question, experimental design, and statistical strategy. A formal sample size calculation is lacking. It should be noted that the use of a single mandible, in which three teeth were evaluated across ten stages of bone loss, does not increase the sample size, although the large number of measurements enhances result precision, improves estimation of systematic error, and increases intra- and interobserver confidence. In light of this, I recommend explicitly stating that no sample size calculation was performed, justifying this decision based on the methodological nature of the study and its emphasis on system error and agreement. It would also be advisable to report inherent study limitations, including convenience sampling and the absence of randomization. I further suggest explicitly structuring eligibility criteria (inclusion/exclusion) in a dedicated subsection, even in an ex vivo study. The identification of variables was appropriate and consistent with the objectives. The explicit use of the cementoenamel junction as the anatomical reference for measurements is commendable, as it enhances clinical relevance.** Comment Group 3 — Methods (Sample size, inclusion criteria, study design)

**Reviewer comment:** The manuscript should address sample size calculation, inclusion/exclusion criteria, convenience sampling, and lack of randomization.

**Response:**
We agree with the reviewer and added a new subsection titled **“Study Design and Sample Considerations”** in the Materials and Methods section. In this subsection, we clarified that this study was designed as a methodological proof-of-concept and system validation study rather than a clinical hypothesis-testing study; therefore, a formal sample size calculation was not performed. We also clarified that the use of a single mandible specimen represents convenience sampling, that no randomization was performed, and that the study design relied on repeated measurements across controlled bone loss stages to quantify system error, agreement, and detection capability. These clarifications improve transparency regarding the experimental design.

**Study design and sample considerations**

This study was designed as a methodological proof-of-concept and system validation study rather than a clinical hypothesis-testing study; therefore, **a** formal priori sample size calculation was not performed. A post hoc power analysis was conducted (see Supporting Information S3) to assess the statistical sensitivity of the experimental design.

The experimental design focused on repeated measurements across controlled bone loss stages to quantify system error, agreement, and detection capability, which were the primary outcomes of this validation study. The use of a single mandible specimen represents convenience sampling, and no randomization was performed. However, the large number of repeated measurements across stages and independent observers allowed robust estimation of measurement variability, system error, and agreement statistics, which were the main objectives of this methodological validation study.

The post hoc power analysis, based on the observed detection threshold (138 µm), system error range (77–113 µm), and repeated-measurement design, indicated a large, standardized effect size (Cohen’s d ≈ 1.22–1.79) and an estimated statistical power exceeding 95%, supporting the adequacy of the study design for detecting clinically relevant bone loss changes.

**Specimen selection criteria**

**A single porcine mandible specimen with intact teeth and surrounding alveolar bone was selected for the *ex vivo* model. Teeth with visible fractures, severe anatomical defects, or existing bone loss were excluded. The specimen was selected to provide stable anatomical landmarks and intact alveolar bone for controlled defect creation and measurement validation.**

**Regarding statistics after completion of the experiment
I recommend justifying the absence of normality tests or, if applicable, stating that normality was assumed based on graphical inspection (e.g., histograms, Q–Q plots).
The hypothesis testing was based on system error, agreement (Bland–Altman), reliability (ICC), and diagnostic testing (accuracy/sensitivity/specificity), which is consistent with method validation studies.
With respect to diagnostic testing, I noted the following limitations that should be acknowledged: a small number of independent events and the absence of confidence intervals for sensitivity and specificity. In this context, I recommend reporting 95% confidence intervals, even if they are wide.
These statistical limitations do not invalidate the results; however, readers should be reminded that external clinical validation has not yet been performed (even if this is implicitly understood).**

# Comment Group 4 — Statistics

**Reviewer comment:** Clarify normality assumptions, confidence intervals for sensitivity/specificity, small number of independent events, and lack of external validation.

**Response:**
We addressed these points as follows:

- We added a statement in the Methods section clarifying that baseline measurement error distributions were inspected for approximate normality before applying the Mean + 2SD system error threshold. Establishing a one-sided upper threshold for measurement noise corresponding to the 97.5th percentile under a normal distribution.
- We clarified that confidence intervals for sensitivity and specificity were calculated where applicable and that these values should be interpreted cautiously due to the limited number of independent bone loss events.
- We added a statement in the Discussion noting that the diagnostic performance analysis was based on a limited number of independent events and that external clinical validation has not yet been performed.
  These additions improve statistical transparency and clarify the methodological limitations of the study.

We also expanded the Discussion to explicitly interpret the limits of agreement in relation to clinically meaningful thresholds, clarifying that although absolute measurements show variability, the system maintains high sensitivity for detecting small longitudinal changes above the system error threshold, which is the clinically relevant objective in periodontal monitoring.

**Agreement analysis and the role of precision versus accuracy**

The regression analyses demonstrated moderate correlation coefficients (R² ≈ 0.41–0.55) between ultrasound measurements and the photographic ground truth. While these values are statistically significant, they indicate only moderate linear association and reflect variability in measurement dispersion rather than direct agreement between methods. Importantly, R² quantifies the strength of correlation but does not assess measurement agreement or interchangeability.

Despite the moderate R² values, the relatively low mean bias observed in the Bland–Altman analysis indicates that systematic error is limited and that the primary source of variability arises from random measurement scatter rather than consistent over- or underestimation. Ultrasound measurements did not consistently overestimate or underestimate bone levels but instead showed variability around the reference measurements. This distinction explains how moderate correlation can coexist with low bias.

The limits of agreement observed in the Bland–Altman analysis indicate that ultrasound measurements should not be considered interchangeable with photographic reference measurements for absolute bone level determination. However, periodontal disease management is primarily concerned with detecting changes in bone levels over time rather than determining a single absolute measurement. In this context, the low system error (77–113 µm) and the small empirical detection threshold (~138 µm) demonstrate high precision in detecting longitudinal changes above the measurement noise floor.

Therefore, the ability of high-frequency ultrasound to detect small changes relative to baseline with low systematic bias is more clinically relevant than achieving high absolute agreement with a reference method. These findings highlight an important distinction between accuracy and precision: while absolute measurements show variability, the system demonstrates high precision for monitoring change over time. Overall, high-frequency ultrasound is best positioned as a longitudinal monitoring tool for tracking small changes in alveolar bone levels, rather than serving as a direct replacement for radiographic methods used for absolute measurements or initial diagnosis.

The limits of agreement (approximately 700–800 µm) are substantially larger than the empirically determined detection threshold (~138 µm), indicating that while absolute measurements exhibit considerable variability, the system retains high sensitivity for detecting small longitudinal changes above the noise floor.

This disparity highlights an important distinction between absolute accuracy and changes detection capability. The relatively wide limits of agreement reflect variability in single-time-point measurements and therefore limit the use of HFUS for precise absolute bone level determination. However, the much smaller detection threshold demonstrates that the system is capable of consistently identifying clinically relevant incremental changes over time.

From a clinical perspective, periodontal disease monitoring primarily relies on detecting progression rather than establishing a single absolute bone level measurement. In this context, the ability to reliably detect changes exceeding the system error threshold is more meaningful than achieving tight agreement with a reference method. Therefore, despite the wide limits of agreement, the measurement characteristics of HFUS support its role as a longitudinal monitoring tool, where relative changes from baseline are of primary importance.

**Regarding the results
There is coherence between the reported results and the variables previously defined in the methods, with no outcomes presented that are unrelated to the methodological framework or not anticipated in the analytical plan.
A clear consistency exists between the scientific question, variable types, statistical tests, and the reported results.
To improve methodological quality and reduce the risk of bias, I recommend explicitly stating whether any data losses or exclusions occurred during the study.
As a point for reflection, a degree of undue extrapolation was perceived in the reporting of results, introducing a mild rhetorical risk in comparisons with conventional radiography. This could likely be mitigated through more careful contextual qualification.**

# Comment Group 5 — Results (Data losses or exclusions)

**Reviewer comment:** Clarify whether any data were excluded.

**Response:**
We added a statement at the beginning of the Results section clarifying that no datasets were excluded from the analysis and that all acquired images and measurements were included in the statistical evaluation.

In addition, we have revised the Results and Discussion to avoid overinterpretation when comparing HFUS with conventional radiography. Specifically, we clarified that radiographic detection thresholds are based on values reported in the literature and that direct comparisons should be interpreted cautiously, as radiography was not evaluated experimentally in this study. This contextual qualification ensures that the conclusions remain appropriately bounded within the scope of the present validation study.

**Results**

The *ex vivo* bone loss model was generated in sequential stages, comprising six stages of bone loss plus one control stage for teeth M3 and PM4 and four stages plus two control stages for tooth M2. No datasets were excluded from the analysis, and all acquired images and measurements were included in the statistical evaluation. The precision of detecting changes was verified using photographic ground truth images, yielding a mean incremental bone loss of 215 ± 35 µm (range: 180-250 µm) per stage.

Statistical comparisons between measurement methods were performed using regression analysis, intraclass correlation coefficient (ICC) analysis, and Bland–Altman agreement analysis. A significance level of P < 0.05 was used.

**Regarding the discussion
The discussion is technically sound, coherent with the results, transparent regarding limitations, and well-grounded in physical, statistical, and clinical principles.
It could be improved by tempering indirect comparisons with radiography, moderating occasionally emphatic language, and reinforcing the experimental/ex vivo nature of the study.**

# Comment Group 6 — Radiography comparison tone

**Reviewer comment:** Comparisons with radiography should be moderated and contextualized.

**Response:**
We agree with the reviewer and revised the Discussion and Conclusion sections to moderate indirect comparisons with conventional radiography. We added a clarification stating that radiography was not directly evaluated in this study and that comparisons with radiographic detection limits are based on values reported in the literature and should be interpreted cautiously. The discussion now emphasizes that high-frequency ultrasound is intended as a complementary monitoring tool rather than a direct replacement for radiography.

The goal of this study was not to replace radiography for absolute bone level measurement, but to demonstrate that high-frequency ultrasound can detect small longitudinal changes in alveolar bone loss with high precision and a defined detection threshold.

**Key study questions: precision, accuracy, and detection capability**

This study addressed three questions:

**Q1. Does high-frequency ultrasound offer a precise and reliable alternative to radiography for monitoring bone loss?**
Our systematic evaluation suggests that HFUS is a precise and reliable method for monitoring alveolar bone loss and may serve as a complementary tool to conventional radiographic methods for longitudinal monitoring. The DTS-US method demonstrated the lowest system error of 77 µm and the automated methods performed comparably or superior to manual measurements while being four times faster. The excellent reliability across all methods (ICC = 0.89-0.99) further establishes HFUS as a robust tool for quantitative periodontal assessment.

**Regarding the conclusion
The conclusion is largely coherent with the research objectives and is based on results obtained using the proposed methodology.
However, conceptual issues arise from indirect comparisons based on the literature (radiography ≠ HFUS). Conventional radiography was not tested in the present study, and although this comparison is appropriately qualified in the main text, the conclusion does not reiterate this caution.**

Comment Group 7 — Conclusion

**Reviewer comment:** The conclusion should avoid overgeneralization regarding radiography.

**Response:**
We revised the Conclusion section to clarify that the study demonstrates measurement precision and detection capability of HFUS in a controlled experimental model and does not directly compare HFUS with radiographic methods within this study. We added a statement emphasizing that comparisons with radiography are based on literature values and that further clinical validation studies are required.

**Conclusion**

This proof-of-concept study demonstrated that high-frequency ultrasound can quantify alveolar bone loss with low system error, strong agreement with photographic measurements, and the ability to detect small incremental changes of approximately 138 µm. These results indicate that high-frequency ultrasound is particularly suitable for longitudinal monitoring of alveolar bone loss progression rather than for single absolute measurements. The automated ultrasound methods showed high reliability and measurement consistency, supporting their potential for future clinical monitoring applications. It should be noted that conventional radiography was not directly evaluated in this study; therefore, comparisons with radiographic detection limits are based on previously published literature and should be interpreted with caution. The present study demonstrates the measurement precision and detection capability of HFUS in a controlled experimental model rather than direct clinical diagnostic superiority. High-frequency ultrasound should therefore be considered a tool for longitudinal monitoring of periodontal bone loss progression rather than a replacement for radiographic assessment of absolute bone levels.

**Regarding the references
The references are well selected and appropriate to the development of the study. Nevertheless, I suggest updating them to include more recent articles, at least up to 2025, and revising their formatting and spelling in accordance with the journal’s reference style guidelines and reference type requirements.**

# Comment Group 8 — References

**Reviewer comment:** References should be updated.

**Response:**
We updated the reference list to include recent literature related to digital dentistry, AI-based segmentation, CBCT analysis, and periodontal ultrasound imaging to better reflect recent developments in the field.

In summary, we revised the manuscript to better reflect the experimental validation nature of the study, added methodological clarifications regarding study design and statistical assumptions, clarified that no data were excluded from analysis, moderated indirect comparisons with radiography, revised the conclusion to avoid overgeneralization, and updated the reference list to include recent literature. These revisions improve methodological transparency and strengthen the presentation of this proof-of-concept validation study.

We sincerely thank the reviewer for these thoughtful and constructive comments, which have significantly improved the clarity, methodological rigor, and clinical interpretation of the manuscript.
