# Supplementary material for: Sub-millimeter quantification of alveolar bone loss using automated 40 MHz high-frequency ultrasound: A proof-of-concept ex vivo validation study
Source: PLoS One. 2026 Jun 8;21(6):e0349815. doi: 10.1371/journal.pone.0349815 (PMC13245748; doi:10.1371/journal.pone.0349815)
Supplement: Attachment — Submitted filename: Response to Reviewers_2nd.docx [file pone.0349815.s006.docx]

**Response to Reviewers**

RESEARCH ARTICLE

**Sub-Millimeter Quantification of Alveolar Bone Loss Using Automated 40 MHz High-Frequency Ultrasound: A Proof-of-Concept Ex Vivo Validation Study**

Tamer Abdelrehim^1^, Baiyan Qi^1^, Bryant Tran^2^, Mirali Pandya^3^, Jesse V. Jokerst^*1,3,4^, Casey Chen^*2^

**1** Aiiso Yufeng Li Family Department of Chemical and NanoEngineering, University of California San Diego, La Jolla, California, United States of America, **2** Herman Ostrow School of Dentistry, University of Southern California, Los Angeles, California, United States of America, **3** Materials Science and Engineering Program, University of California San Diego, La Jolla, California, United States of America, **4** Department of Radiology, University of California San Diego, La Jolla, California, United States of America

* [ccchen@usc.edu](mailto:ccchen@usc.edu), [jjokerst@ucsd.edu](mailto:jjokerst@ucsd.edu)

We would like to thank the academic editor and the reviewers for their previous careful evaluation of our manuscript and for their constructive and insightful comments. We have revised the manuscript thoroughly according to the reviewers’ suggestions. We believe that these revisions have significantly more improved the clarity, structure, and scientific presentation of the manuscript.

Below we provide a detailed, point-by-point response to each reviewer comment. All changes have been incorporated into the revised manuscript and are indicated in the tracked changes version.

Reviewer comment: **with bold black**.

Author Response: with regular black.

New text edited at the manuscript: with regular blue

**Reviewer #1:**

**With the help of the previous Reviewers' comments and recommendations, this revised and re-submitted draft could be considerably improved. Should be ready to proceed now. Congrats, and stay healthy!**

Response:

We sincerely thank the reviewer for the positive evaluation of our revised manuscript and for recognizing the improvements made based on the previous round of comments. We appreciate the encouraging feedback and are pleased that the manuscript is now considered ready to proceed.

We are grateful for the reviewer’s constructive input throughout the review process, which has significantly strengthened the clarity, structure, and overall quality of the work.

Change in manuscript:

No Change.

**Reviewer #2:**

**(No Response).**

Response to Reviewer #2

We sincerely thank the reviewer for their time and for confirming that all previous comments have been adequately addressed. We greatly appreciate the constructive feedback provided in the earlier review rounds, which has helped improve the clarity and quality of the manuscript.

Change in manuscript:

No Change.

**Reviewer #3:**

**Dear authors,
Thank you so much for all the revision and new version of your article.
I have no further comments or questions.

Best regards.**

Response to Reviewer #3

We sincerely thank the reviewer for their thoughtful evaluation and kind feedback. We greatly appreciate the time and effort invested in reviewing our manuscript throughout the revision process. We are pleased that the revised version has addressed all concerns, and we are grateful for the positive assessment.

Change in manuscript:

No Change.

**Reviewer #5:**

**(No Response).**

Response:

We sincerely thank the reviewer for their time and for confirming that all previous comments have been adequately addressed. We greatly appreciate the constructive feedback provided in the earlier review rounds, which has helped improve the clarity and quality of the manuscript.

Change in manuscript:

No Change.

## **Reviewer #6:**

**I thank the authors for their careful review of the manuscript. The updated version presents clear improvements in structure, greater clarity, and alignment between objectives, methods, and results. The manuscript now presents a more balanced and technically sound discussion, and the overall scientific contribution is evident.

However, some important methodological limitations remain insufficiently addressed or not explicitly stated, compromising the transparency of the study. Given the proof-of-concept and ex vivo nature of the work, these issues do not invalidate the conclusions, but should be clearly acknowledged to ensure proper interpretation by clinical readers and researchers who wish to use this reference for further study.

I strongly recommend, in the interest of a more transparent scientific method, that the authors explicitly include the following points in the limitations section (within the Discussion section):

The use of a single specimen (n=1), clarifying that repeated measurements do not increase the number of independent samples (pseudoreplication).

The convenience sampling approach and its implications for generalization.

The absence of experimental randomization, differentiating it from the randomization used for blind image evaluation. A justification for the absence of normality tests or clarification of any assumptions made (especially relevant for ANOVA/ICC-based analyses).

The absence of confidence intervals (e.g., 95%) for sensitivity and specificity, especially considering the limited number of independent events.

A clear statement about exclusions or data loss, even if none occurred.

These additions are relatively small from a structural point of view, but are essential for methodological transparency and adherence to good reporting practices.

Final recommendation: Accept after minor revisions.**

### **Response:**

We thank the reviewer for this constructive recommendation. We have revised the Limitations section to clarify the structure of the dataset and its implications for statistical interpretation. Specifically, we now state that the study was conducted on a single porcine mandible specimen, including multiple teeth analyzed across controlled bone-loss stages.

## **Reviewer point: Single specimen and pseudoreplication**

Reviewer:

### **1. The use of a single specimen (n=1), clarifying that repeated measurements do not increase the number of independent samples (pseudoreplication).**

### **Response:**

We further clarify that, although multiple teeth, stages, and repeated measurements were included, these observations do not represent independent biological samples and should be interpreted within a repeated-measurement framework. The potential for pseudoreplication is now explicitly acknowledged.

Importantly, we emphasize that this study was designed as a proof-of-concept validation of measurement performance under controlled conditions. Biological factors such as tissue viability, inflammation, soft tissue composition, and in vivo scanning conditions may influence ultrasound signal interaction and image characteristics. For example, imaging in a living model may introduce additional variability compared to an ex vivo specimen. However, these factors are expected to affect measurement conditions rather than the underlying measurement principle. Therefore, while biological variability may influence quantitative performance metrics, the measurement framework and methodology remain applicable across conditions.

These clarifications have been incorporated in the Limitations section of the revised manuscript to ensure full methodological transparency.

### **Change in manuscript:**

**Section:** Discussion → Limitations

**Added paragraph:**

Specifically, we now state that the study was conducted on multiple teeth within a single porcine mandible, with measurements performed across controlled bone-loss stages. We further clarify that, although multiple teeth, stages, and repeated measurements were included, these observations do not represent independent biological samples and should be interpreted within a repeated-measurement framework. The potential for pseudoreplication is now explicitly acknowledged. While this design enables detailed evaluation of measurement performance, it does not capture biological variation between different animals. However, this study was designed as a proof-of-concept validation of measurement performance under controlled conditions, and the measurement framework and methodology remain applicable across conditions.

## **Reviewer point: Convenience sampling**

Reviewer:

**2. The convenience sampling approach and its implications for generalization.**

### **Response:**

We have added a statement clarifying that the specimen was selected by convenience sampling based on availability, intact anatomy, and suitability for controlled defect creation. The implications of this approach for generalizability are now explicitly discussed in the Limitations section.

### **Change in manuscript:**

**Section:** Discussion → Limitations

**Added (within same paragraph):**

The specimen was selected by convenience sampling based on availability, intact anatomy, and suitability for controlled defect creation. This limits the generalizability of the findings to broader biological variation, different tooth types, different ages, and clinical periodontal conditions.

## **Reviewer point: Absence of experimental randomization**

Reviewer:

**3. The absence of experimental randomization, differentiating it from the randomization used for blind image evaluation. A justification for the absence of normality tests or clarification of any assumptions made (especially relevant for ANOVA/ICC-based analyses).**

### **Response:**

We have clarified that no experimental randomization was performed for specimen selection or defect creation. We further distinguish this from the randomization applied during blinded image evaluation, where images were anonymized and presented in random order to reduce observer bias.

### **Change in manuscript:**

**Section:** Discussion → Limitations

**Added (within same paragraph):**

No experimental randomization was performed for specimen selection or defect creation. The randomization used in this study applied only to the blinded image-evaluation process, where images were anonymized and presented in random order to reduce observer bias.

## **Reviewer point: Normality assumptions**

### **Response:**

We thank the reviewer for this important comment. We have clarified the assumptions related to normality and the use of ANOVA/ICC analyses in two locations. First, a statement has been added in the Data and Statistical Analysis section to explain that formal normality testing was not systematically performed due to the limited number of independent experimental units, and that analyses were applied within a repeated-measurement reliability framework. Second, this point has been explicitly acknowledged in the Limitations section to ensure transparency regarding statistical assumptions and interpretation.

### **Change in manuscript:**

**Section:** Data and Statistical Analysis

**Added:**

Due to the limited number of independent experimental units inherent to this proof-of-concept design, formal normality testing was not systematically performed. Instead, baseline error distributions were visually inspected and found to be approximately normal. Accordingly, ANOVA- and ICC-based analyses were applied as measures of internal consistency and reliability within a repeated-measurement framework rather than for population-level inference.

**Section:** Discussion → Limitations

**Added (within main paragraph):**

Formal normality testing was not performed for all statistical comparisons due to the limited number of independent experimental units. Baseline error distributions were visually inspected and appeared approximately normally distributed. Therefore, ANOVA- and ICC-based results should be interpreted as measures of internal consistency and reliability within this controlled repeated-measurement framework rather than as population-level inference.

## **Reviewer point: Confidence intervals for sensitivity/specificity**

Reviewer:

**4. The absence of confidence intervals (e.g., 95%) for sensitivity and specificity, especially considering the limited number of independent events.**

### **Response:**

We thank the reviewer for this important comment. Confidence intervals are already reported for reliability metrics (e.g., ICC values, Table 3), where sufficient repeated measurements were available. For diagnostic performance metrics (sensitivity and specificity), these values are presented and interpreted in detail in the Results and Discussion sections (Tables 4–6). However, due to the limited number of independent bone-loss events inherent to this proof-of-concept design, confidence interval estimates for sensitivity, and specificity would be wide and less reliable. Therefore, rather than over-interpreting statistically unstable interval estimates, we have clarified this limitation in the manuscript and emphasized that the reported diagnostic accuracy metrics should be interpreted as preliminary.

### **Change in manuscript:**

**Section:** Clinical Translation and Detection Performance

**Added:**

Due to the limited number of independent bone-loss events evaluated in this proof-of-concept study, confidence intervals for sensitivity and specificity would be wide and therefore less precise. Accordingly, these estimates should be interpreted with caution and considered preliminary indicators of detection performance rather than definitive measures of diagnostic accuracy.

**Section:** Discussion → Limitations

**Added (within main paragraph):**

Confidence intervals for sensitivity and specificity are inherently limited by the small number of independent bone-loss events and may therefore be wide and less reliable.

## **Reviewer point: Exclusions or data loss**

Reviewer:

**5. A clear statement about exclusions or data loss, even if none occurred.**

### **Response:**

We have added an explicit statement confirming that no datasets, images, or measurements were excluded from the analysis and that no data loss occurred.

### **Change in manuscript:**

**Section:** Results

**Already present:**

No datasets were excluded from the analysis, and all acquired images and measurements were included in the statistical evaluation.

**Section:** Discussion → Limitations

**Added:**

No datasets, images, or measurements were excluded from the analysis, and no data loss occurred.

## **Final note**

**These additions are relatively small from a structural point of view but are essential for methodological transparency and adherence to good reporting practices.

Final recommendation: Accept after minor revisions.**

### **Response:**

We are grateful for the reviewer’s insightful feedback, which has significantly improved the transparency, clarity, and methodological rigor of the manuscript.

**Additional note:** We added new reference [41] into our reference list. And refer to it at section: “ Anatomical considerations and prior work”
