# Supplementary material for: Sub-millimeter quantification of alveolar bone loss using automated 40 MHz high-frequency ultrasound: A proof-of-concept ex vivo validation study
Source: PLoS One. 2026 Jun 8;21(6):e0349815. doi: 10.1371/journal.pone.0349815 (PMC13245748; doi:10.1371/journal.pone.0349815)
Supplement: Peer review history

# Peer review: Decision Letter 0

**DOI:** 10.1371/journal.pone.0349815.r001

**Authors:** Andrej Kielbassa

16 Feb 2026

PONE-D-26-02614

Quantification of Alveolar Bone Loss Using High-Frequency Ultrasonography for Precision Periodontal Monitoring

PLOS One

Dear Dr. Jokerst,

Thank you for submitting your manuscript to PLOS ONE. After careful consideration, we feel that it has merit but does not fully meet PLOS ONE’s publication criteria as it currently stands. Therefore, we invite you to submit a revised version of the manuscript that addresses the points raised during the review process.

Please find our external Reviewers' comments and recommendations forwarded below. You will see that the full spectrum of final decisions ranges from 'minor revisions' to 'rejection', so please revise your draft carefully but thoroughly, and re-submit an elaborated response sticking to each and every comment given below. Please remember that one review is considered standard, and that revised drafts not considered satisfying must lead to 'outright reject'.

Please submit your revised manuscript by Apr 02 2026 11:59PM. If you will need more time than this to complete your revisions, please reply to this message or contact the journal office at plosone@plos.org. When you're ready to submit your revision, log on to https://www.editorialmanager.com/pone/ and select the 'Submissions Needing Revision' folder to locate your manuscript file.

Please include the following items when submitting your revised manuscript:

- A letter that responds to each point raised by the academic editor and reviewer(s). You should upload this letter as a separate file labeled 'Response to Reviewers'.
- A marked-up copy of your manuscript that highlights changes made to the original version. You should upload this as a separate file labeled 'Revised Manuscript with Track Changes'.
- An unmarked version of your revised paper without tracked changes. You should upload this as a separate file labeled 'Manuscript'.

If you would like to make changes to your financial disclosure, please include your updated statement in your cover letter. Guidelines for resubmitting your figure files are available below the reviewer comments at the end of this letter.

If applicable, we recommend that you deposit your laboratory protocols in protocols.io to enhance the reproducibility of your results. Protocols.io assigns your protocol its own identifier (DOI) so that it can be cited independently in the future. For instructions see: https://journals.plos.org/plosone/s/submission-guidelines#loc-laboratory-protocols. Additionally, PLOS ONE offers an option for publishing peer-reviewed Lab Protocol articles, which describe protocols hosted on protocols.io. Read more information on sharing protocols at https://plos.org/protocols?utm_medium=editorial-email&utm_source=authorletters&utm_campaign=protocols.

We look forward to receiving your revised manuscript.

Kind regards,

Prof. Dr. Dr. h. c. Andrej M Kielbassa

Academic Editor

PLOS One

Journal Requirements:

When submitting your revision, we need you to address these additional requirements.

1. Please ensure that your manuscript meets PLOS ONE's style requirements, including those for file naming. The PLOS ONE style templates can be found at

https://journals.plos.org/plosone/s/file?id=wjVg/PLOSOne_formatting_sample_main_body.pdf and

https://journals.plos.org/plosone/s/file?id=ba62/PLOSOne_formatting_sample_title_authors_affiliations.pdf

2. Please note that PLOS One has specific guidelines on code sharing for submissions in which author-generated code underpins the findings in the manuscript. In these cases, we expect all author-generated code to be made available without restrictions upon publication of the work. Please review our guidelines at https://journals.plos.org/plosone/s/materials-and-software-sharing#loc-sharing-code and ensure that your code is shared in a way that follows best practice and facilitates reproducibility and reuse.

3. Please update your submission to use the PLOS LaTeX template. The template and more information on our requirements for LaTeX submissions can be found at http://journals.plos.org/plosone/s/latex.

4. Thank you for stating the following in the Acknowledgments Section of your manuscript:

“This research was funded by the National Institute of Dental and Craniofacial Research (NIDCR) grant number R01DE031561.”

We note that you have provided funding information that is not currently declared in your Funding Statement. However, funding information should not appear in the Acknowledgments section or other areas of your manuscript. We will only publish funding information present in the Funding Statement section of the online submission form.

Please remove any funding-related text from the manuscript and let us know how you would like to update your Funding Statement. Currently, your Funding Statement reads as follows:

“This work was supported by the National Institute of Dental and Craniofacial Research (NIDCR), National Institutes of Health (NIH), grant R01DE031561, awarded to J.V.J. and C.C. (https://www.nidcr.nih.gov). The funders had no role in study design, data collection and analysis, decision to publish, or preparation of the manuscript.”

Please include your amended statements within your cover letter; we will change the online submission form on your behalf.

5. When completing the data availability statement of the submission form, you indicated that you will make your data available on acceptance. We strongly recommend all authors decide on a data sharing plan before acceptance, as the process can be lengthy and hold up publication timelines. Please note that, though access restrictions are acceptable now, your entire data will need to be made freely accessible if your manuscript is accepted for publication. This policy applies to all data except where public deposition would breach compliance with the protocol approved by your research ethics board. If you are unable to adhere to our open data policy, please kindly revise your statement to explain your reasoning and we will seek the editor's input on an exemption. Please be assured that, once you have provided your new statement, the assessment of your exemption will not hold up the peer review process.

6. Please include a separate caption for each figure in your manuscript.

7. Please include your tables as part of your main manuscript and remove the individual files. Please note that supplementary tables (should remain/ be uploaded) as separate "supporting information" files

8. Please include captions for your Supporting Information files at the end of your manuscript, and update any in-text citations to match accordingly. Please see our Supporting Information guidelines for more information: http://journals.plos.org/plosone/s/supporting-information.

9. If the reviewer comments include a recommendation to cite specific previously published works, please review and evaluate these publications to determine whether they are relevant and should be cited. There is no requirement to cite these works unless the editor has indicated otherwise.

[Note: HTML markup is below. Please do not edit.]

Reviewers' comments:

Reviewer's Responses to Questions

**Comments to the Author**

1. Is the manuscript technically sound, and do the data support the conclusions?

The manuscript must describe a technically sound piece of scientific research with data that supports the conclusions. Experiments must have been conducted rigorously, with appropriate controls, replication, and sample sizes. The conclusions must be drawn appropriately based on the data presented.

Reviewer #1: No

Reviewer #2: Yes

Reviewer #3: Yes

Reviewer #4: Partly

Reviewer #5: Yes

Reviewer #6: Yes

**********

2. Has the statistical analysis been performed appropriately and rigorously?

Reviewer #1: No

Reviewer #2: I Don't Know

Reviewer #3: No

Reviewer #4: Yes

Reviewer #5: Yes

Reviewer #6: Yes

**********

3. Have the authors made all data underlying the findings in their manuscript fully available?

The PLOS Data policy requires authors to make all data underlying the findings described in their manuscript fully available without restriction, with rare exception (please refer to the Data Availability Statement in the manuscript PDF file). The data should be provided as part of the manuscript or its supporting information, or deposited to a public repository. For example, in addition to summary statistics, the data points behind means, medians and variance measures should be available. If there are restrictions on publicly sharing data—e.g. participant privacy or use of data from a third party—those must be specified.

Reviewer #1: No

Reviewer #2: Yes

Reviewer #3: Yes

Reviewer #4: Yes

Reviewer #5: Yes

Reviewer #6: Yes

**********

4. Is the manuscript presented in an intelligible fashion and written in standard English?

PLOS ONE does not copyedit accepted manuscripts, so the language in submitted articles must be clear, correct, and unambiguous. Any typographical or grammatical errors should be corrected at revision, so please note any specific errors here.

Reviewer #1: Yes

Reviewer #2: Yes

Reviewer #3: Yes

Reviewer #4: Yes

Reviewer #5: Yes

Reviewer #6: Yes

**********

5. Review Comments to the Author

Please use the space provided to explain your answers to the questions above. You may also include additional comments for the author, including concerns about dual publication, research ethics, or publication ethics. (Please upload your review as an attachment if it exceeds 20,000 characters)

Reviewer #1: Title

- "Quantification of Alveolar Bone Loss Using High-Frequency Ultrasonography for Precision Periodontal Monitoring" - please stick to Plos One's Guidelines for Authors at https://journals.plos.org/plosone/s/submission-guidelines. No capital letters.

- Please provide type of study.

Abstract

- With your current 174 words, you do not provide sufficient information. Please stick to the 300-word count allowed for with this section.

- Provide sound methodology, and stick to exact results, along with exact P values-

- With your Conclusions, please stick exclusively to your aims. Do not simply repeat your results here. Do not speculate. Do not provide well-accepted (but meaningless) phrases. Instead, provide a reasonable and generalizable extension of your outcome.

Intro

- Your "Clinical Relevance/Background:" does not stick to Journal style. Please consult some recently published Plos One papers, and revise carefully.

- Same with "Added Value of This Study:" and "Clinical Implications:".

- Same with your references. "(...) change for detection ¹." must read "(...) change for detection [1]."

- Same with "1. INTRODUCTION". Again, stick to the Journal's guidelines, and consult some recently published Plos One papers. Revise carefully, but thoroughly.

- Please provide a sound null hypothesis.

Meths

- Again, please compare "2. Materials and Methods", and revise for uniform Journal style.

- With ALL materials (including chemicals) and methodologies (including statistical software), please use general/non-proprietary names with your text, followed by (brand name; manufacturer, city, ST[ate - abbreviated, if US], country) in parentheses. Stick to semicolon. Ensure reproducibility, and revise thoroughly.

- "h (height) x w (width)" must read "h (height) × w (width)". Please use symbol × instead of letter x. Revise thoroughly throughout your text.

Results

- With your P values, please stick to Journal style.

Disc

- What about H0?

- "Our systematic evaluation confirms that HFUS is a precise and reliable alternative." Alternative to what?

- What about the strengths of your study?

- What about the limitations?

- Please provide sound responses to your teaching objectives.

Concl

- Again, with your Conclusions, please stick exclusively to your aims. Do not simply repeat your results here. Do not speculate. Do not provide well-accepted (but meaningless) phrases. Instead, provide a reasonable and generalizable extension of your outcome.

Refs

- Revise for sound and uniform Journal style. Again, stick to Authors' Guidelines.

In total, this draft would seem interesting, but would not seem ready to proceed. Sticking to the Journal's guidelines would seem mandatory. Please remember that Authors (but neither Reviewers nor Editors or Typesetters)) are responsible for sound drafts, and note that one review is considered standard.

Reviewer #2: This study is fundamental research toward quantifying alveolar bone resorption using high-frequency ultrasound. As stated in the conclusion, it holds the potential to revolutionize future periodontal disease diagnosis. Specifically, conventional diagnosis using two-dimensional images from X-rays primarily detects alveolar bone resorption centered on the mesio-distal regions of teeth where the root and alveolar bone do not overlap. This study demonstrates results enabling effective detection of alveolar bone resorption in the buccal and lingual central regions, areas where early bone resorption is difficult to detect. However, considering actual clinical settings, challenges remain for the practical implementation of alveolar bone resorption detection using this system.

1. Reference markers were fixed into drilled holes using cyanoacrylate adhesive (Gorilla Glue/Super Glue) and positioned as reference markers 3-4mm above the initial alveolar bone level on teeth M3, M2, and PM4. However, on M3, M2, and PM4, where exactly on the buccal side (mesial, central, or distal) were these markers placed? Furthermore, this reference 20 does not appear to be an appropriate citation.

2. Related to the above point, when the current analysis site has a relatively simple morphology, it raises the question of whether high-frequency ultrasound can detect bone resorption within complex three-dimensional periodontal structures, such as adjacent interdental spaces and furcations, which are common sites for periodontitis.

3. Inflammatory bone resorption due to periodontitis dissolves the superficial cortical bone and causes complex changes in the structure of the cancellous bone. The alveolar bone resorption induced in this study resulted from artificial bone removal using a chisel. It is possible that this method may not adequately reflect the marginal bone loss caused by alveolar bone resorption associated with actual soft tissue inflammation, particularly at the bone margin. For instance, an analysis using experimental periodontitis in animal models would provide a more realistic analytical model reflecting actual alveolar bone resorption. Therefore, it would be ideal if the validity of the findings could be assessed in such a model.

4. The limitations of this study are not mentioned in the final section of the discussion. For this model to be effectively applied in clinical practice, there must be outstanding issues that must be overcome.

Reviewer #3: Dear authors,

I evaluated the article titled “Quantification of Alveolar Bone Loss Using High-Frequency Ultrasonography for Precision Periodontal Monitoring.” The goal was "to establish its system error, detection capability, and agreement with a photographic ground truth."

This is an interesting study! Therefore, some concerns were raised.

A concern was raised: is there necessity/justification for this proof of concept study? The justification presented by the authors was robust. They have successfully addressed the "Goldilocks" zone of periodontal imaging: (1) resolution: it was demonstrated a detection threshold (~138 μm) that is nearly 6x more sensitive than traditional radiography (~870 μm); (2) safety: it removed the ionizing radiation barrier, allowing for the "repetitive monitoring" that is currently impossible with CBCT; and (3) automation: by using SVD-filtering and automated CEJ-tracking, you address the primary criticism of ultrasound: operator subjectivity.

Revised Article - per section

1. Title: I suggest a modification… recommended: Sub-Millimeter Quantification of Alveolar Bone Loss using Automated 40 MHz High-Frequency Ultrasound: A Proof-of-Concept Study. Please check it

2. Abstract: it was condensed and with high-impact

3. Keywords: I suggest to include at least one of the following: "Precision Medicine," "Non-ionizing Radiation," or "Digital Dentistry"

4. Introduction:

- Ensure you emphasize that periodontitis is a dynamic disease, but our current tools (radiography) are static and insensitive.

- "The 'diagnostic window' for early intervention is frequently missed because conventional radiographs require a 30-50% mineral loss before a defect becomes visible. This study introduces a 40 MHz HFUS workflow that bridges this gap by offering a theoretical axial resolution of 40 μm."

- “Periodontitis is one of the two leading diseases in dentistry, along with dental caries 2,3 resulting in pain, tooth loss 4, diminished quality of life5, and possible systemic effects such as Type II diabetes 6 and cardiovascular disease7. In patients suffering from periodontitis, bacteria-induced inflammatory responses destroy soft and hard tissues supporting the teeth (i.e., the periodontium)8.” WHY 8 references in the first paragraph? Reduce it for 3.

5. MM: it is necessary a structural refinement:

- The SVD innovation: the authors must explicitly state why SVD was used. It wasn't just for "noise reduction" – it was to extract the coherent topographic signal from the "clutter" of speckle noise.

- Mathematical notation: Ensure your SVD equation A=UΣVT is clearly defined as the "economy-size" decomposition to show computational efficiency.

6. Results & Discussion:

- In your discussion, you must address the "Precision vs. Accuracy" paradox you found: Precision (high): excellent for tracking change over time; Accuracy (variable): Challenging for single absolute measurements.

- The pitch: argue that in periodontology, progression (delta) is more clinically significant than a single baseline value. This justifies why HFUS is the perfect "monitoring" tool even if it doesn't replace the initial radiographic survey.

Key Revisions Checklist for Top-Tier Submission

Statistical rigor: Ensure Table A1 (Bland-Altman) includes Proportion of Bias. If the bias is only -18 μm on a 200 μm change, that is a <10% error – a huge selling point.

- Explicitly mention that Method 2 (Auto-SVD) removes the need for fiducial markers (steel balls), making it the "clinically viable" path.

7. References: due to the topic studied, ensure you cite recent (2023-2025) AI-segmentation papers to show how your SVD approach complements current trends.

Reviewer #4: High frequency ultrasonography (HFUS) for alveolar bone loss is an emerging, non-invasive imaging technique being explored as an alternative or adjunct to radiographs in periodontal diagnosis. It allows real-time visualization of the alveolar crest, buccal bone thickness, and soft tissues without radiation. HFUS for periodontal and peri-implant bone assessment has been actively researched by several ex vivo and clinical studies. The preclinical diagnostic accuracy studies have already showed good agreement between HFUS and imaging for crestal bone thickness and alveolar bone distance measurements repeatedly. While this study is well executed, it does not provide sufficient novelty or significance to the diagnosis of periodontal or peri-implant diseases. Larger, standardized clinical trials are needed to fully validate diagnostic thresholds and automated measurement software.

Reviewer #5: The manuscript is scientifically sound and methodologically robust. The ex vivo model is appropriate for a proof-of-concept study, and the authors apply multiple complementary measurement strategies, which strengthens internal validity.

My main concern relates to interpretative balance. The detection capability is promising. However, the limits of agreement reported in the Bland–Altman analyses are relatively wide, suggesting that the technology may be better suited for longitudinal monitoring of changes over time rather than direct substitution of photographic reference methods for absolute measurements. This distinction should be stated more explicitly in the Discussion.

Overall, the manuscript meets the journal’s standards for methodological rigor. The requested revisions aim to improve clarity and transparency rather than address fundamental flaws.

1. Clear differentiation of statistical concepts: system error, minimum detectable change, and empirical detection threshold.

In the Methods (pp. 16–17) and Results (p. 20), the manuscript introduces multiple related statistical metrics:

System error (Mean + 2SD of baseline), minimum detectable change, empirical detection threshold (~138 µm).

While each appears methodologically justified, the distinctions between these concepts are not fully explicit. This may lead to interpretative ambiguity.

Please:

Provide clear definitions of each metric.

Explain how they relate conceptually.

Clarify which metric should be interpreted as the clinically meaningful threshold for longitudinal monitoring.

Explicitly state whether the minimum detectable change was calculated using the conventional formula (e.g., 1.96 × √2 × SD) or whether the threshold reported is purely empirical.

A clearer statistical framework will substantially improve transparency.

2. Interpretation of regression analyses (R² values)

In the regression analyses comparing ultrasound-based measurements with photographic ground truth (p. 18), R² values range approximately between 0.41 and 0.55.

Although statistically significant, these values indicate moderate correlation.

Please expand the discussion to address:

The methodological meaning of these coefficients.

How moderate R² values should be interpreted in light of low mean bias.

Whether these correlation levels support clinical interchangeability or rather reinforce the role of HFUS as a longitudinal monitoring tool.

The manuscript would benefit from a more explicit and balanced interpretation of these findings.

3. Clinical implications of Bland–Altman limits of agreement

The Bland–Altman analyses (p. 19) demonstrate limits of agreement approaching approximately 700–800 µm in certain comparisons.

While acknowledged, the clinical implications of these intervals require clearer contextualization.

Please:

Discuss whether these intervals allow true interchangeability with photographic reference measurements.

Explicitly differentiate between suitability for absolute measurement and suitability for change detection over time.

Clarify how these limits compare to clinically meaningful thresholds in periodontal assessment.

This distinction is essential for appropriate interpretation.

4. Algorithm transparency and reproducibility

The Auto-SVD-US method (pp. 13–16) is described in detail, which is commendable.

To enhance reproducibility:

Clearly specify which algorithm parameters are fixed and which are user-defined.

Indicate whether the implementation code (e.g., MATLAB scripts) or pseudocode will be made available.

If parameter tuning was performed, describe the selection process.

This will strengthen methodological transparency.

5. Justification of the Mean + 2SD threshold

Please indicate whether baseline data distribution was tested for normality before defining system error as Mean + 2SD. If tested, briefly describe the method used.

6. Expansion of limitations regarding the ex vivo model

Although the experimental model is well described (pp. 10–12), please expand the Discussion to briefly address additional in vivo considerations such as:

Soft tissue variability

Saliva presence

Probe angulation variability

Differences in acoustic impedance in clinical conditions.

7. Translational integration into clinical workflow

Consider adding a brief statement regarding:

Estimated acquisition time per site

Operator training requirements

Practical considerations for clinical implementation

Reviewer #6: Thank you for the opportunity to review the manuscript submitted under the title “Quantification of Alveolar Bone Loss Using High-Frequency Ultrasonography for Precision Periodontal Monitoring.” Below, I present my impressions derived from a careful reading of the text, outlining my interpretations and, where appropriate, offering recommendations for the authors’ consideration.

Regarding the title

The submitted article title employs clear, elegant, and technically accurate language, appropriately highlighting the central technology (HFUS) and the clinical context of periodontal monitoring with increased precision, while remaining concise.

However, some limitations warrant reflection. The title may be overly generic for an experimental validation study, omitting essential information for experienced readers and methodological reviewers (such as the sample, comparator, and study design). As a result, it does not fully convey the originality or experimental rigor of the work.

Regarding the introduction and objectives

Overall, the research rationale and literature exploration are adequate and well substantiated, with a clear logical progression and consistent use of references to support the scientific problem.

Suggestion for improvement:

Although the literature is well explored, the introduction could benefit from an explicit sentence comparing HFUS with previous attempts at periodontal ultrasonography, even if only to highlight their limitations (e.g., insufficient frequency, low resolution, lack of quantitative validation). This would further strengthen the originality of the study.

The relevance and contribution of the study are, in general, very well-articulated, both from clinical and technological perspectives, clearly justifying the need for publication.

The formulation and adequacy of the objectives are clear and methodologically aligned with the stated rationale.

Regarding the methods

A high level of coherence was observed between the introduction, objectives, and the methods presented, with no misalignment between the research question, experimental design, and statistical strategy.

A formal sample size calculation is lacking. It should be noted that the use of a single mandible, in which three teeth were evaluated across ten stages of bone loss, does not increase the sample size, although the large number of measurements enhances result precision, improves estimation of systematic error, and increases intra- and interobserver confidence.

In light of this, I recommend explicitly stating that no sample size calculation was performed, justifying this decision based on the methodological nature of the study and its emphasis on system error and agreement.

It would also be advisable to report inherent study limitations, including convenience sampling and the absence of randomization.

I further suggest explicitly structuring eligibility criteria (inclusion/exclusion) in a dedicated subsection, even in an ex vivo study.

The identification of variables was appropriate and consistent with the objectives. The explicit use of the cementoenamel junction as the anatomical reference for measurements is commendable, as it enhances clinical relevance.

Regarding statistics after completion of the experiment

I recommend justifying the absence of normality tests or, if applicable, stating that normality was assumed based on graphical inspection (e.g., histograms, Q–Q plots).

The hypothesis testing was based on system error, agreement (Bland–Altman), reliability (ICC), and diagnostic testing (accuracy/sensitivity/specificity), which is consistent with method validation studies.

With respect to diagnostic testing, I noted the following limitations that should be acknowledged: a small number of independent events and the absence of confidence intervals for sensitivity and specificity. In this context, I recommend reporting 95% confidence intervals, even if they are wide.

These statistical limitations do not invalidate the results; however, readers should be reminded that external clinical validation has not yet been performed (even if this is implicitly understood).

Regarding the results

There is coherence between the reported results and the variables previously defined in the methods, with no outcomes presented that are unrelated to the methodological framework or not anticipated in the analytical plan.

A clear consistency exists between the scientific question, variable types, statistical tests, and the reported results.

To improve methodological quality and reduce the risk of bias, I recommend explicitly stating whether any data losses or exclusions occurred during the study.

As a point for reflection, a degree of undue extrapolation was perceived in the reporting of results, introducing a mild rhetorical risk in comparisons with conventional radiography. This could likely be mitigated through more careful contextual qualification.

Regarding the discussion

The discussion is technically sound, coherent with the results, transparent regarding limitations, and well-grounded in physical, statistical, and clinical principles.

It could be improved by tempering indirect comparisons with radiography, moderating occasionally emphatic language, and reinforcing the experimental/ex vivo nature of the study.

Regarding the conclusion

The conclusion is largely coherent with the research objectives and is based on results obtained using the proposed methodology.

However, conceptual issues arise from indirect comparisons based on the literature (radiography ≠ HFUS). Conventional radiography was not tested in the present study, and although this comparison is appropriately qualified in the main text, the conclusion does not reiterate this caution.

Regarding the references

The references are well selected and appropriate to the development of the study. Nevertheless, I suggest updating them to include more recent articles, at least up to 2025, and revising their formatting and spelling in accordance with the journal’s reference style guidelines and reference type requirements.

**********

6. PLOS authors have the option to publish the peer review history of their article (what does this mean?). If published, this will include your full peer review and any attached files.

If you choose “no”, your identity will remain anonymous but your review may still be made public.

**Do you want your identity to be public for this peer review?** For information about this choice, including consent withdrawal, please see our Privacy Policy.

Reviewer #1: No

Reviewer #2: No

Reviewer #3: No

Reviewer #4: No

Reviewer #5: No

Reviewer #6: No

**********

[NOTE: If reviewer comments were submitted as an attachment file, they will be attached to this email and accessible via the submission site. Please log into your account, locate the manuscript record, and check for the action link "View Attachments". If this link does not appear, there are no attachment files.]

To ensure your figures meet our technical requirements, please review our figure guidelines: https://journals.plos.org/plosone/s/figures

You may also use PLOS’s free figure tool, NAAS, to help you prepare publication quality figures: https://journals.plos.org/plosone/s/figures#loc-tools-for-figure-preparation.

NAAS will assess whether your figures meet our technical requirements by comparing each figure against our figure specifications.

# Author comment: Author response to Decision Letter 1

**DOI:** 10.1371/journal.pone.0349815.r002

13 Apr 2026

Subject: Revised Manuscript Submission – PONE-D-26-02614

Dear Prof. Kielbassa,

Thank you for the opportunity to revise and resubmit our manuscript (PONE-D-26-02614). We have carefully revised the manuscript according to the reviewers’ and editor’s comments and have addressed each comment in detail in the response-to-reviewers document.

The revised submission includes:

1. Response to Reviewers

2. Revised Manuscript with Track Changes

3. Clean Manuscript

4. Updated Figures and Captions

5. Supporting Information files

6. Updated Funding Statement and Data Availability information

We appreciate the reviewers’ constructive feedback, which has significantly improved the manuscript. We hope the revised version is now suitable for publication in PLOS ONE.

Sincerely,

Jesse V. Jokerst

Attachment Submitted filename: Response to Reviewers_Final.docx

# Peer review: Decision Letter 1

**DOI:** 10.1371/journal.pone.0349815.r003

**Authors:** Andrej Kielbassa

20 Apr 2026

PONE-D-26-02614R1

Sub-millimeter quantification of alveolar bone loss using automated 40 MHz high-frequency ultrasound: a proof-of-concept ex vivo validation study

PLOS One

Dear Dr. Jokerst,

Thank you for submitting your manuscript to PLOS ONE. After careful consideration, we feel that it has merit but still does not fully meet PLOS ONE’s publication criteria as it currently stands. Therefore, we invite you to submit a revised version of the manuscript that addresses the points raised during the review process.

- Please note that (while several Referees have been satisfied with your re-submitted draft) one of our external Reviewers has indicated that still some minor aspects would seem perfectible.
- I just have double checked those comments, and your surely will agree that the latter are justified.

Please submit your revised manuscript by Jun 04 2026 11:59PM. If you will need more time than this to complete your revisions, please reply to this message or contact the journal office at plosone@plos.org. When you're ready to submit your revision, log on to https://www.editorialmanager.com/pone/ and select the 'Submissions Needing Revision' folder to locate your manuscript file.

Please include the following items when submitting your revised manuscript:

- A letter that responds to each point raised by the academic editor and reviewer(s). You should upload this letter as a separate file labeled 'Response to Reviewers'.
- A marked-up copy of your manuscript that highlights changes made to the original version. You should upload this as a separate file labeled 'Revised Manuscript with Track Changes'.
- An unmarked version of your revised paper without tracked changes. You should upload this as a separate file labeled 'Manuscript'.

If you would like to make changes to your financial disclosure, please include your updated statement in your cover letter. Guidelines for resubmitting your figure files are available below the reviewer comments at the end of this letter.

If applicable, we recommend that you deposit your laboratory protocols in protocols.io to enhance the reproducibility of your results. Protocols.io assigns your protocol its own identifier (DOI) so that it can be cited independently in the future. For instructions see: https://journals.plos.org/plosone/s/submission-guidelines#loc-laboratory-protocols. Additionally, PLOS ONE offers an option for publishing peer-reviewed Lab Protocol articles, which describe protocols hosted on protocols.io. Read more information on sharing protocols at https://plos.org/protocols?utm_medium=editorial-email&utm_source=authorletters&utm_campaign=protocols.

As the corresponding author, your ORCID iD is verified in the submission system and will appear in the published article. PLOS supports the use of ORCID, and we encourage all coauthors to register for an ORCID iD and use it as well. Please encourage your coauthors to verify their ORCID iD within the submission system before final acceptance, as unverified ORCID iDs will not appear in the published article. *Only* the individual author can complete the verification step; PLOS staff *cannot* verify ORCID iDs on behalf of authors.

We look forward to receiving your revised manuscript.

Kind regards,

Andrej M Kielbassa

Academic Editor

PLOS One

Journal Requirements:

1. If the reviewer comments include a recommendation to cite specific previously published works, please review and evaluate these publications to determine whether they are relevant and should be cited. There is no requirement to cite these works unless the editor has indicated otherwise.

2. Please review your reference list to ensure that it is complete and correct. If you have cited papers that have been retracted, please include the rationale for doing so in the manuscript text, or remove these references and replace them with relevant current references. Any changes to the reference list should be mentioned in the rebuttal letter that accompanies your revised manuscript. If you need to cite a retracted article, indicate the article’s retracted status in the References list and also include a citation and full reference for the retraction notice.

[Note: HTML markup is below. Please do not edit.]

Reviewers' comments:

Reviewer's Responses to Questions

**Comments to the Author**

1. If the authors have adequately addressed your comments raised in a previous round of review and you feel that this manuscript is now acceptable for publication, you may indicate that here to bypass the “Comments to the Author” section, enter your conflict of interest statement in the “Confidential to Editor” section, and submit your "Accept" recommendation.

Reviewer #1: All comments have been addressed

Reviewer #2: All comments have been addressed

Reviewer #3: All comments have been addressed

Reviewer #5: All comments have been addressed

Reviewer #6: (No Response)

**********

2. Is the manuscript technically sound, and do the data support the conclusions?

The manuscript must describe a technically sound piece of scientific research with data that supports the conclusions. Experiments must have been conducted rigorously, with appropriate controls, replication, and sample sizes. The conclusions must be drawn appropriately based on the data presented.

Reviewer #1: Yes

Reviewer #2: (No Response)

Reviewer #3: Yes

Reviewer #5: Yes

Reviewer #6: Partly

**********

3. Has the statistical analysis been performed appropriately and rigorously?

Reviewer #1: Yes

Reviewer #2: (No Response)

Reviewer #3: Yes

Reviewer #5: Yes

Reviewer #6: No

**********

4. Have the authors made all data underlying the findings in their manuscript fully available?

The PLOS Data policy requires authors to make all data underlying the findings described in their manuscript fully available without restriction, with rare exception (please refer to the Data Availability Statement in the manuscript PDF file). The data should be provided as part of the manuscript or its supporting information, or deposited to a public repository. For example, in addition to summary statistics, the data points behind means, medians and variance measures should be available. If there are restrictions on publicly sharing data—e.g. participant privacy or use of data from a third party—those must be specified.

Reviewer #1: Yes

Reviewer #2: (No Response)

Reviewer #3: Yes

Reviewer #5: Yes

Reviewer #6: Yes

**********

5. Is the manuscript presented in an intelligible fashion and written in standard English?

PLOS ONE does not copyedit accepted manuscripts, so the language in submitted articles must be clear, correct, and unambiguous. Any typographical or grammatical errors should be corrected at revision, so please note any specific errors here.

Reviewer #1: Yes

Reviewer #2: (No Response)

Reviewer #3: Yes

Reviewer #5: Yes

Reviewer #6: Yes

**********

6. Review Comments to the Author

Please use the space provided to explain your answers to the questions above. You may also include additional comments for the author, including concerns about dual publication, research ethics, or publication ethics. (Please upload your review as an attachment if it exceeds 20,000 characters)

Reviewer #1: With the help of the previous Reviewers' comments and recommendations, this revised and re-submitted draft could be considerably improved. Should be ready to proceed now. Congrats, and stay healthy!

Reviewer #2: (No Response)

Reviewer #3: Dear authors,

Thank you so much for all the revision and new version of your article.

I have no further comments or questions.

Best regards.

Reviewer #5: (No Response)

Reviewer #6: I thank the authors for their careful review of the manuscript. The updated version presents clear improvements in structure, greater clarity, and alignment between objectives, methods, and results. The manuscript now presents a more balanced and technically sound discussion, and the overall scientific contribution is evident.

However, some important methodological limitations remain insufficiently addressed or not explicitly stated, compromising the transparency of the study. Given the proof-of-concept and ex vivo nature of the work, these issues do not invalidate the conclusions, but should be clearly acknowledged to ensure proper interpretation by clinical readers and researchers who wish to use this reference for further study.

I strongly recommend, in the interest of a more transparent scientific method, that the authors explicitly include the following points in the limitations section (within the Discussion section):

The use of a single specimen (n=1), clarifying that repeated measurements do not increase the number of independent samples (pseudoreplication).

The convenience sampling approach and its implications for generalization.

The absence of experimental randomization, differentiating it from the randomization used for blind image evaluation. A justification for the absence of normality tests or clarification of any assumptions made (especially relevant for ANOVA/ICC-based analyses).

The absence of confidence intervals (e.g., 95%) for sensitivity and specificity, especially considering the limited number of independent events.

A clear statement about exclusions or data loss, even if none occurred.

These additions are relatively small from a structural point of view, but are essential for methodological transparency and adherence to good reporting practices.

Final recommendation: Accept after minor revisions.

**********

7. PLOS authors have the option to publish the peer review history of their article (what does this mean?). If published, this will include your full peer review and any attached files.

If you choose “no”, your identity will remain anonymous but your review may still be made public.

**Do you want your identity to be public for this peer review?** For information about this choice, including consent withdrawal, please see our Privacy Policy.

Reviewer #1: No

Reviewer #2: No

Reviewer #3: **Yes:** Gustavo Fernandes

Reviewer #5: No

Reviewer #6: No

**********

[NOTE: If reviewer comments were submitted as an attachment file, they will be attached to this email and accessible via the submission site. Please log into your account, locate the manuscript record, and check for the action link "View Attachments". If this link does not appear, there are no attachment files.]

To ensure your figures meet our technical requirements, please review our figure guidelines: https://journals.plos.org/plosone/s/figures

You may also use PLOS’s free figure tool, NAAS, to help you prepare publication quality figures: https://journals.plos.org/plosone/s/figures#loc-tools-for-figure-preparation.

NAAS will assess whether your figures meet our technical requirements by comparing each figure against our figure specifications.

# Author comment: Author response to Decision Letter 2

**DOI:** 10.1371/journal.pone.0349815.r004

2 May 2026

(Note: this section will be repeated again at this combined PDF file with a font color as the attachment "Response to Reviewers" , but it is mandatory to be pasted here as a required information at the journal submission "Comments" )

Response to Reviewers

RESEARCH ARTICLE

Sub-Millimeter Quantification of Alveolar Bone Loss Using Automated 40 MHz High-Frequency Ultrasound: A Proof-of-Concept Ex Vivo Validation Study

Tamer Abdelrehim1, Baiyan Qi1, Bryant Tran2, Mirali Pandya3, Jesse V. Jokerst*1,3,4, Casey Chen*2

1 Aiiso Yufeng Li Family Department of Chemical and NanoEngineering, University of California San Diego, La Jolla, California, United States of America, 2 Herman Ostrow School of Dentistry, University of Southern California, Los Angeles, California, United States of America, 3 Materials Science and Engineering Program, University of California San Diego, La Jolla, California, United States of America, 4 Department of Radiology, University of California San Diego, La Jolla, California, United States of America

* ccchen@usc.edu, jjokerst@ucsd.edu

We would like to thank the academic editor and the reviewers for their previous careful evaluation of our manuscript and for their constructive and insightful comments. We have revised the manuscript thoroughly according to the reviewers’ suggestions. We believe that these revisions have significantly more improved the clarity, structure, and scientific presentation of the manuscript.

Below we provide a detailed, point-by-point response to each reviewer comment. All changes have been incorporated into the revised manuscript and are indicated in the tracked changes version.

Reviewer comment: with bold black.

Author Response: with regular black.

New text edited at the manuscript: with regular blue

Reviewer #1:

With the help of the previous Reviewers' comments and recommendations, this revised and re-submitted draft could be considerably improved. Should be ready to proceed now. Congrats, and stay healthy!

Response:

We sincerely thank the reviewer for the positive evaluation of our revised manuscript and for recognizing the improvements made based on the previous round of comments. We appreciate the encouraging feedback and are pleased that the manuscript is now considered ready to proceed.

We are grateful for the reviewer’s constructive input throughout the review process, which has significantly strengthened the clarity, structure, and overall quality of the work.

Change in manuscript:

No Change.

Reviewer #2:

(No Response).

Response to Reviewer #2

We sincerely thank the reviewer for their time and for confirming that all previous comments have been adequately addressed. We greatly appreciate the constructive feedback provided in the earlier review rounds, which has helped improve the clarity and quality of the manuscript.

Change in manuscript:

No Change.

Reviewer #3:

Dear authors,

Thank you so much for all the revision and new version of your article.

I have no further comments or questions.

Best regards.

Response to Reviewer #3

We sincerely thank the reviewer for their thoughtful evaluation and kind feedback. We greatly appreciate the time and effort invested in reviewing our manuscript throughout the revision process. We are pleased that the revised version has addressed all concerns, and we are grateful for the positive assessment.

Change in manuscript:

No Change.

Reviewer #5:

(No Response).

Response:

We sincerely thank the reviewer for their time and for confirming that all previous comments have been adequately addressed. We greatly appreciate the constructive feedback provided in the earlier review rounds, which has helped improve the clarity and quality of the manuscript.

Change in manuscript:

No Change.

Reviewer #6:

I thank the authors for their careful review of the manuscript. The updated version presents clear improvements in structure, greater clarity, and alignment between objectives, methods, and results. The manuscript now presents a more balanced and technically sound discussion, and the overall scientific contribution is evident.

However, some important methodological limitations remain insufficiently addressed or not explicitly stated, compromising the transparency of the study. Given the proof-of-concept and ex vivo nature of the work, these issues do not invalidate the conclusions, but should be clearly acknowledged to ensure proper interpretation by clinical readers and researchers who wish to use this reference for further study.

I strongly recommend, in the interest of a more transparent scientific method, that the authors explicitly include the following points in the limitations section (within the Discussion section):

The use of a single specimen (n=1), clarifying that repeated measurements do not increase the number of independent samples (pseudoreplication).

The convenience sampling approach and its implications for generalization.

The absence of experimental randomization, differentiating it from the randomization used for blind image evaluation. A justification for the absence of normality tests or clarification of any assumptions made (especially relevant for ANOVA/ICC-based analyses).

The absence of confidence intervals (e.g., 95%) for sensitivity and specificity, especially considering the limited number of independent events.

A clear statement about exclusions or data loss, even if none occurred.

These additions are relatively small from a structural point of view, but are essential for methodological transparency and adherence to good reporting practices.

Final recommendation: Accept after minor revisions.

Response:

We thank the reviewer for this constructive recommendation. We have revised the Limitations section to clarify the structure of the dataset and its implications for statistical interpretation. Specifically, we now state that the study was conducted on a single porcine mandible specimen, including multiple teeth analyzed across controlled bone-loss stages.

Reviewer point: Single specimen and pseudoreplication

Reviewer:

1. The use of a single specimen (n=1), clarifying that repeated measurements do not increase the number of independent samples (pseudoreplication).

Response:

We further clarify that, although multiple teeth, stages, and repeated measurements were included, these observations do not represent independent biological samples and should be interpreted within a repeated-measurement framework. The potential for pseudoreplication is now explicitly acknowledged.

Importantly, we emphasize that this study was designed as a proof-of-concept validation of measurement performance under controlled conditions. Biological factors such as tissue viability, inflammation, soft tissue composition, and in vivo scanning conditions may influence ultrasound signal interaction and image characteristics. For example, imaging in a living model may introduce additional variability compared to an ex vivo specimen. However, these factors are expected to affect measurement conditions rather than the underlying measurement principle. Therefore, while biological variability may influence quantitative performance metrics, the measurement framework and methodology remain applicable across conditions.

These clarifications have been incorporated in the Limitations section of the revised manuscript to ensure full methodological transparency.

Change in manuscript:

Section: Discussion → Limitations

Added paragraph:

Specifically, we now state that the study was conducted on multiple teeth within a single porcine mandible, with measurements performed across controlled bone-loss stages. We further clarify that, although multiple teeth, stages, and repeated measurements were included, these observations do not represent independent biological samples and should be interpreted within a repeated-measurement framework. The potential for pseudoreplication is now explicitly acknowledged. While this design enables detailed evaluation of measurement performance, it does not capture biological variation between different animals. However, this study was designed as a proof-of-concept validation of measurement performance under controlled conditions, and the measurement framework and methodology remain applicable across conditions.

Reviewer point: Convenience sampling

Reviewer:

2. The convenience sampling approach and its implications for generalization.

Response:

We have added a statement clarifying that the specimen was selected by convenience sampling based on availability, intact anatomy, and suitability for controlled defect creation. The implications of this approach for generalizability are now explicitly discussed in the Limitations section.

Change in manuscript:

Section: Discussion → Limitations

Added (within same paragraph):

The specimen was selected by convenience sampling based on availability, intact anatomy, and suitability for controlled defect creation. This limits the generalizability of the findings to broader biological variation, different tooth types, different ages, and clinical periodontal conditions.

Reviewer point: Absence of experimental randomization

Reviewer:

3. The absence of experimental randomization, differentiating it from the randomization used for blind image evaluation. A justification for the absence of normality tests or clarification of any assumptions made (especially relevant for ANOVA/ICC-based analyses).

Response:

We have clarified that no experimental randomization was performed for specimen selection or defect creation. We further distinguish this from the randomization applied during blinded image evaluation, where images were anonymized and presented in random order to reduce observer bias.

Change in manuscript:

Section: Discussion → Limitations

Added (within same paragraph):

No experimental randomization was performed for specimen selection or defect creation. The randomization used in this study applied only to the blinded image-evaluation process, where images were anonymized and presented in random order to reduce observer bias.

Reviewer point: Normality assumptions

Response:

We thank the reviewer for this important comment. We have clarified the assumptions related to normality and the use of ANOVA/ICC analyses in two locations. First, a statement has been added in the Data and Statistical Analysis section to explain that formal normality testing was not systematically performed due to the limited number of independent experimental units, and that analyses were applied within a repeated-measurement reliability framework. Second, this point has been explicitly acknowledged in the Limitations section to ensure transparency regarding statistical assumptions and interpretation.

Change in manuscript:

Section: Data and Statistical Analysis

Added:

Due to the limited number of independent experimental units inherent to this proof-of-concept design, formal normality testing was not systematically performed. Instead, baseline error distributions were visually inspected and found to be approximately normal. Accordingly, ANOVA- and ICC-based analyses were applied as measures of internal consistency and reliability within a repeated-measurement framework rather than for population-level inference.

Section: Discussion → Limitations

Added (within main paragraph):

Formal normality testing was not performed for all statistical comparisons due to the limited number of independent experimental units. Baseline error distributions were visually inspected and appeared approximately normally distributed. Therefore, ANOVA- and ICC-based results should be interpreted as measures of internal consistency and reliability within this controlled repeated-measurement framework rather than as population-level inference.

Reviewer point: Confidence intervals for sensitivity/specificity

Reviewer:

4. The absence of confidence intervals (e.g., 95%) for sensitivity and specificity, especially considering the limited number of independent events.

Response:

We thank the reviewer for this important comment. Confidence intervals are already reported for reliability metrics (e.g., ICC values, Table 3), where sufficient repeated measurements were available. For diagnostic performance metrics (sensitivity and specificity), these values are presented and interpreted in detail in the Results and Discussion sections (Tables 4–6). However, due to the limited number of independent bone-loss events inherent to this proof-of-concept design, confidence interval estimates for sensitivity, and specificity would be wide and less reliable. Therefore, rather than over-interpreting statistically unstable interval estimates, we have clarified this limitation in the manuscript and emphasized that the reported diagnostic accuracy metrics should be interpreted as preliminary.

Change in manuscript:

Section: Clinical Translation and Detection Performance

Added:

Due to the limited number of independent bone-loss events evaluated in this proof-of-concept study, confidence intervals for sensitivity and specificity would be wide and therefore less precise. Accordingly, these estimates should be interpreted with caution and considered preliminary indicators of detection performance rather than definitive measures of diagnostic accuracy.

Section: Discussion → Limitations

Added (within main paragraph):

Confidence intervals for sensitivity and specificity are inherently limited by the small number of independent bone-loss events and may therefore be wide and less reliable.

Reviewer point: Exclusions or data loss

Reviewer:

5. A clear statement about exclusions or data loss, even if none occurred.

Response:

We have added an explicit statement confirming that no datasets, images, or measurements were excluded from the analysis and that no data loss occurred.

Change in manuscript:

Section: Results

Already present:

No datasets were excluded from the analysis, and all acquired images and measurements were included in the statistical evaluation.

Section: Discussion → Limitations

Added:

No datasets, images, or measurements were excluded from the analysis, and no data loss occurred.

Final note

These additions are relatively small from a structural point of view but are essential for methodological transparency and adherence to good reporting practices.

Final recommendation: Accept after minor revisions.

Response:

We are grateful for the reviewer’s insightful feedback, which has significantly improved the transparency, clarity, and methodological rigor of the manuscript.

Additional note: We added new reference [41] into our reference list. And refer to it at section: “ Anatomical considerations and prior work”

Attachment Submitted filename: Response to Reviewers_2nd.docx

# Peer review: Decision Letter 2

**DOI:** 10.1371/journal.pone.0349815.r005

**Authors:** Andrej Kielbassa

5 May 2026

Sub-millimeter quantification of alveolar bone loss using automated 40 MHz high-frequency ultrasound: a proof-of-concept ex vivo validation study

PONE-D-26-02614R2

Dear Dr. Jokerst,

We’re pleased to inform you that your manuscript has been judged scientifically suitable for publication and will be formally accepted for publication once it meets all outstanding technical requirements.

Within one week, you’ll receive an e-mail detailing the required amendments. When these have been addressed, you’ll receive a formal acceptance letter and your manuscript will be scheduled for publication.

An invoice will be generated when your article is formally accepted. Please note, if your institution has a publishing partnership with PLOS and your article meets the relevant criteria, all or part of your publication costs will be covered. Please make sure your user information is up-to-date by logging into Editorial Manager at Editorial Manager® and clicking the ‘Update My Information' link at the top of the page. For questions related to billing, please contact billing support.

If your institution or institutions have a press office, please notify them about your upcoming paper to help maximize its impact. If they’ll be preparing press materials, please inform our press team as soon as possible -- no later than 48 hours after receiving the formal acceptance. Your manuscript will remain under strict press embargo until 2 pm Eastern Time on the date of publication. For more information, please contact onepress@plos.org.

Kind regards,

Andrej M Kielbassa

Academic Editor

PLOS One

Additional Editor Comments (optional):

Reviewers' comments:

Reviewer's Responses to Questions

**Comments to the Author**

1. If the authors have adequately addressed your comments raised in a previous round of review and you feel that this manuscript is now acceptable for publication, you may indicate that here to bypass the “Comments to the Author” section, enter your conflict of interest statement in the “Confidential to Editor” section, and submit your "Accept" recommendation.

Reviewer #1: All comments have been addressed

Reviewer #6: All comments have been addressed

**********

2. Is the manuscript technically sound, and do the data support the conclusions?

The manuscript must describe a technically sound piece of scientific research with data that supports the conclusions. Experiments must have been conducted rigorously, with appropriate controls, replication, and sample sizes. The conclusions must be drawn appropriately based on the data presented.

Reviewer #1: Yes

Reviewer #6: Yes

**********

3. Has the statistical analysis been performed appropriately and rigorously?

Reviewer #1: Yes

Reviewer #6: Yes

**********

4. Have the authors made all data underlying the findings in their manuscript fully available?

The PLOS Data policy requires authors to make all data underlying the findings described in their manuscript fully available without restriction, with rare exception (please refer to the Data Availability Statement in the manuscript PDF file). The data should be provided as part of the manuscript or its supporting information, or deposited to a public repository. For example, in addition to summary statistics, the data points behind means, medians and variance measures should be available. If there are restrictions on publicly sharing data—e.g. participant privacy or use of data from a third party—those must be specified.

Reviewer #1: Yes

Reviewer #6: Yes

**********

5. Is the manuscript presented in an intelligible fashion and written in standard English?

PLOS ONE does not copyedit accepted manuscripts, so the language in submitted articles must be clear, correct, and unambiguous. Any typographical or grammatical errors should be corrected at revision, so please note any specific errors here.

Reviewer #1: Yes

Reviewer #6: Yes

**********

6. Review Comments to the Author

Please use the space provided to explain your answers to the questions above. You may also include additional comments for the author, including concerns about dual publication, research ethics, or publication ethics. (Please upload your review as an attachment if it exceeds 20,000 characters)

Reviewer #1: The Co-Authors have adequately responded to all previous and recent comments and recommendation. From my point of view, this revised and re-submitted manuscript could be considerably improved with the help of the Reviewers. In case Reviewer #2 will confirm this view, this piece of work should be worth following, and is considered ready to proceed.

Thanks, and good luck with your future work!

Reviewer #6: The manuscript has clearly improved compared to its earlier versions, particularly in terms of methodological transparency and the way the study’s limitations are now presented. The authors have made a consistent effort to address previous concerns, especially by explicitly discussing key issues such as the ex vivo nature of the model, the use of a convenience sample, the reliance on a single biological unit (n = 1) with the associated risk of pseudoreplication, the lack of full experimental randomization, the constraints related to the statistical assumptions, and the limited precision of the diagnostic estimates, particularly sensitivity and specificity. Altogether, these revisions strengthen the manuscript and bring it more in line with good scientific reporting practices, allowing for a more balanced interpretation of the findings.

From a scientific perspective, the study offers a meaningful, though preliminary, contribution by exploring the potential of an emerging diagnostic approach. The results are interesting and suggest possible applicability, but they should be interpreted with caution. Given the inherent limitations of the study design, the level of certainty of the evidence remains low, especially when considering its translation to real clinical settings.

Looking ahead, further research will be important to build on these findings. Studies that include biologically independent and more representative samples, are conducted under in vivo conditions, and use analytical methods that properly account for data dependency will be particularly valuable. In addition, efforts to validate the diagnostic accuracy of this method in real clinical environments, with more robust estimates and appropriate confidence intervals, will be essential to better understand its true clinical value.

In its current form, the manuscript is suitable for publication and represents a valid initial step in this line of research. At the same time, its conclusions should be viewed as preliminary, and future studies with stronger methodological designs and external validation will be necessary to consolidate the evidence in this area.

**********

7. PLOS authors have the option to publish the peer review history of their article (what does this mean?). If published, this will include your full peer review and any attached files.

If you choose “no”, your identity will remain anonymous but your review may still be made public.

**Do you want your identity to be public for this peer review?** For information about this choice, including consent withdrawal, please see our Privacy Policy.

Reviewer #1: No

Reviewer #6: No

**********

# Editor report: Acceptance letter

**DOI:** 10.1371/journal.pone.0349815.r006

**Authors:** Andrej Kielbassa

PONE-D-26-02614R2

PLOS One

Dear Dr. Jokerst,

I'm pleased to inform you that your manuscript has been deemed suitable for publication in PLOS One. Congratulations! Your manuscript is now being handed over to our production team.

At this stage, our production department will prepare your paper for publication. This includes ensuring the following:

* All references, tables, and figures are properly cited

* All relevant supporting information is included in the manuscript submission,

* There are no issues that prevent the paper from being properly typeset

You will receive further instructions from the production team, including instructions on how to review your proof when it is ready. Please keep in mind that we are working through a large volume of accepted articles, so please give us a few days to review your paper and let you know the next and final steps.

Lastly, if your institution or institutions have a press office, please let them know about your upcoming paper now to help maximize its impact. If they'll be preparing press materials, please inform our press team within the next 48 hours. Your manuscript will remain under strict press embargo until 2 pm Eastern Time on the date of publication. For more information, please contact onepress@plos.org.

You will receive an invoice from PLOS for your publication fee after your manuscript has reached the completed accept phase. If you receive an email requesting payment before acceptance or for any other service, this may be a phishing scheme. Learn how to identify phishing emails and protect your accounts at https://explore.plos.org/phishing.

If we can help with anything else, please email us at customercare@plos.org.

Thank you for submitting your work to PLOS ONE and supporting open access.

Kind regards,

PLOS ONE Editorial Office Staff

on behalf of

Prof. Dr. med. dent. Dr. h. c. Andrej M Kielbassa

Academic Editor

PLOS One
